# Adaptability, Scalability and Sustainability (ASaS) of complex health interventions: a systematic review of theories, models and frameworks

**DOI:** 10.1186/s13012-024-01375-7

**Published:** 2024-07-17

**Authors:** Lixin Sun, Andrew Booth, Katie Sworn

**Affiliations:** Sheffield Centre for Health and Related Research (SCHARR), University of Sheffield, Regent Court, 30 Regent Street, Sheffield, S1 4DA UK

**Keywords:** Adaptability, Scalability, Sustainability, Complex health interventions, Influencing factors

## Abstract

**Background:**

Complex health interventions (CHIs) are increasingly used in public health, clinical research and education to reduce the burden of disease worldwide. Numerous theories, models and frameworks (TMFs) have been developed to support implementation of CHIs.

This systematic review aims to identify and critique theoretical frameworks concerned with three features of implementation; adaptability, scalability and sustainability (ASaS). By dismantling the constituent theories, analysing their component concepts and then exploring factors that influence each theory the review team hopes to offer an enhanced understanding of considerations when implementing CHIs.

**Methods:**

This review searched PubMed MEDLINE, CINAHL, Web of Science, and Google Scholar for research investigating the TMFs of complex health interventions. Narrative synthesis was employed to examine factors that may influence the adaptability, scalability and sustainability of complex health interventions.

**Results:**

A total of 9763 studies were retrieved from the five databases (PubMed, MEDLINE, CINAHL, Web of Science, and Google Scholar). Following removal of duplicates and application of the eligibility criteria, 35 papers were eligible for inclusion. Influencing factors can be grouped within outer context (socio-political context; leadership funding, inter-organisational networks), inner context; (client advocacy; organisational characteristics), intervention characteristics (supervision, monitoring and evaluation), and bridging factors (individual adopter or provider characteristics).

**Conclusion:**

This review confirms that identified TMFS do not typically include the three components of adaptability, scalability, and sustainability. Current approaches focus on high income countries or generic “whole world” approaches with few frameworks specific to low- and middle-income countries. The review offers a starting point for further exploration of adaptability, scalability and sustainability, within a low- and middle-income context.

**Trial registration:**

Not registered.

**Supplementary Information:**

The online version contains supplementary material available at 10.1186/s13012-024-01375-7.

Contributions to the literature

- This study identified that current existing theories, models and frameworks (TMFs) focus on high income countries or generic “whole world” approaches with few frameworks specific to low- and middle-income countries.
- This study explored the factors influencing the adaptability, scalability and sustainability of complex health interventions within current TMFs.
- This study evaluated the applicability and feasibility of current TMF in low- and middle-income countries.

## Introduction

This systematic review examines the adaptability, scalability, and sustainability (ASaS) of complex health interventions (CHIs), which are increasingly used in public health, clinical research, and education to alleviate global disease burdens [1]. The effectiveness of CHIs depends on various factors, including health resources, education levels, and economic status [2, 3].

CHIs are interventions with multiple interacting components, posing unique evaluation challenges beyond the usual practical and methodological difficulties [4].

Adaptability, scalability, and sustainability are crucial concepts in implementing CHIs, addressed through stages of evidence efficacy, scaling-up, and long-term sustainability [5]. Initial research phases focus on adapting interventions to local contexts and needs [6]. Once effectiveness is proven, the goal shifts to broader implementation, aiming for sustainability in real-world settings [5].The definitions of the ASaS is shown in the Table 1.

**Table 1:** The definitions of key concepts in this study

Concepts	Definitions
Adaptability	"the degree to which an intervention can be adapted, modified, or tailored to meet the needs of various contexts and populations while retaining its essential elements" [7].
Scalability	Scalability is defined by the World Health Organisation and ExpandNet [8] as “deliberate efforts to increase the impact of health service innovations successfully tested in pilot or experimental projects so as to benefit more people and to foster policy and programme development on a lasting basis”.
Sustainability	Scheirer and Dearing [9] (2011) defined sustainability as the “Continued use of intervention components and activities for the continued achievement of desirable health outcomes within the population of interest.”

Theories, models and frameworks are used extensively to advance implementation science [10–13], to guide the design and implementation of complex interventions, and to help in assessing their quality. The resultant models can also be used to elucidate causal mechanisms between influencing factors and to identify contextual factors associated with changes in outcomes [10, 11, 14]. In turn, TMFs offer a lens for the exploration of the complex fields of public health, health policy and social care [10, 13].

Generally, a theory is:

“a set of inter‐related concepts, definitions and propositions that present a systematic view of events or situations by specifying relations among variables, to explain and predict the events or situations [15, 16].

Simply put, theories, are closely related to models. Specially, theories are characterized as combining the explanatory alongside the descriptive, and models are defined as theories with a narrowly defined scope of explanation [16]. A framework is:

“a structure, overview, outline, system or plan consisting of various descriptive categories including concepts, constructs or variables, and the relations between them that are presumed to account for a phenomenon” [16, 17].

Compared with theories and models, frameworks do not seek to be explanatory; rather than *describe* the internal relationships of concepts, they simply *present* the concepts [16]. However, despite some diverse characteristics, the terms “theory”, “model” and “framework” (TMF) are often used interchangeably.

However, most existing frameworks and measures for determining implementation factors and outcomes have originated in high-income nations [18]. A study indicated that although the number of frameworks related to program sustainability is increasing, these frameworks are rarely applied and evaluated in low- and middle-income countries or vulnerable communities within high-income countries. The primary reason for this phenomenon is the unique challenges faced by these countries and regions in terms of community and workplace capacities [19]. Therefore, it cannot be assumed that current TMFs are suitable for resource-limited settings [20].

Consequently, the aim of this review is to conduct a systematic review of theoretical frameworks concerned with at least one of the three ASaS concepts, to deconstruct the constituent theories, and to analyze the influencing factors within these frameworks. Another aim of this study is to assess the applicability and feasibility of these TMFs in diverse settings.

Specifically, the objectives of this review are:

1. To explore definitions of scalability, adaptability, and sustainability.
2. To identify published theoretical studies concerned with at least one of the concepts of sustainability, scalability and adaptability of complex health intervention-related frameworks and to assemble and explore relevant models and frameworks;
3. To explore inter-relationships between factors influencing scalability, adaptability, and sustainability of the complex health interventions;
4. To analyse the applicability and feasibility of these TMFs;
5. To appraise the methodological quality and reporting quality of the included literature.

## Methods

### Search strategy

Systematic review methods were employed to identify and select TMFs. Specifically, the BeHEMoTh procedure was used as a systematic approach by which to collect theoretical frameworks [10]. The BeHEMoTh procedure offers auditability and transparency when identifying published TMFs [21]. Specific features of the BeHEMoTh search process are outlined in Additional file 1. The search begins with a structured BeHEMoTh question. First, the researcher reviewed TMFs identified from a scoping review in order to construct a systematic search procedure for retrieving ASaS related TMFs via Google Scholar [Step 1a]. PubMed MEDLINE, CINAHL and Web of Science, were systematically searched using the same search strategy, in a process similar to a conventional systematic review search [step 1b]. Titles and abstracts were screened for TMFs using a spreadsheet with each additional instance being added to the list of TMFs previously identified via step 1 [step 2]. Named models retrieved from step 2, together with models found from scoping via Google Scholar, were then searched to retrieve additional *related* reports [step 3]. Searching of source references for these TMFs was used to reveal *cited* studies [step 4a and step 4b].

### Search terms

Search terms across all sources were organized within five search term groups including the three ASaS concepts (See Additional file 1). By searching for the three ASaS concepts individually rather than for their intersect, the search strategy recognises that few models involve all three factors of ASaS with many involving one or two factors.

### Inclusion and exclusion criteria

Identified publications were imported to Endnote 9 software and duplicates were deleted. Specific inclusion criteria for factors that influence ASaS of CHIs are shown in Table 2.

**Table 2:** Eligibility criteria

	Inclusion Criteria	Exclusion criteria
Intervention type	∙ Studies relate to implementation or implementation science or complex interventions including at least one model or framework	∙ Studies do not relate to implementation or implementation science or complex interventions
Publication type	∙ Reviews ∙ Books ∙ Journal articles ∙ Gray literature	∙ editorials ∙ commentaries ∙ poster presentations ∙ protocol papers
Study type	∙ conceptual papers ∙ theoretical papers ∙ reviews ∙ concept analysis ∙ Case study ∙ Qualitative study evaluation with conceptual elements ∙ Quantitative study evaluation with conceptual elements ∙ Method papers	∙ Qualitative study or evaluation without conceptual elements ∙ Quantitative study or evaluation without conceptual elements
Types of models and frameworks	∙ significantly modified or updated an older framework; ∙ inductive formulation of a new framework from two or more older frameworks;	∙ statistical models ∙ disease models ∙ reports of an existing framework without modification
Discipline	∙ Health or education or international development or social services	∙ None human science
Study outcomes	∙ No limitation	
Publication dates	∙ Subsequent to first MRC guideline on complex interventions (2000)	
Publication language	∙ English	∙ Not English

### Data extraction and appraisal

The titles and abstracts were screened, and the full papers of potentially relevant studies were obtained. Two authors independently assessed 10% of all titles and abstracts with a single reviewer then selecting full text papers for eligibility. An initial data extraction form was modified and adopted after revision. A single researcher independently extracted: (1) Study identification: year of publication, authors, name of study and name of the theories, models and frameworks; (2) Methods: study design, and study context; (3) any TMFs used; (4) Purpose of the theories, models, and framework; (5) Theories, models, and frameworks: definition, conceptual model, framework; (6) factors influencing ASaS of CHIs and inter-relationships between these concepts. These tables are shown in Additional file 3.

### Quality assessment

Quality assessment criteria for assessing reports of TMFs are not common. Three papers were identified that either develop or utilize criteria for assessing theories [22–24] and these papers were used to compile the following quality assessment criteria:

1. Is the methodology identified and justified?
2. Was a theoretical lens or perspective used to guide the study, with a reference provided?
3. Is the theoretical framework described?
4. Is the theoretical framework easily linked with the problem?
5. If a conceptual framework is used, are the concepts adequately defined?
6. Are the relationships among concepts clearly identified?
7. Are the influencing factors of concepts clearly identified?
8. Are the relationships among influencing factors clearly described?

Quality assessments were undertaken by a single reviewer, quality assessment judgements are reported in Additional file 4.

### Analysis

Given that the literature relating to TMFs derives from multiple disciplines, the researcher decided to use a narrative synthesis approach, which allows for synthesis of diverse types, designs and contexts for studies [25–27].

First, collected TMFs were categorized against a pre-existing classification: (1) Process models; (2) Determinant frameworks; (3) Classic theories; (4) Implementation theories; (5) Evaluation frameworks [16] (Table 3).

**Table 3:** Classification of models used in implementation science

**Type of model**	**Description**	**Example(s)**
(1) Process models;	Represent idealised step-by-step, sequential, and linear interpretation of implementation and typically depict developer experience from implementing projects	Knowledge-to-action Framework/Quality Implementation Framework
(2) Determinant frameworks;	Typically describe variables associated with implementation outcome. Generally, do not depict causal relationships. Operate at multiple levels: individuals, institutions, etc. They are based on implementation barriers and contributing factors from original research together with each developer's implementation experience [28]. Some aggregate multiple frameworks [6, 29].	CFIR framework
(3) Classic theories;	Borrow classical theories from psychology, sociology and organisational science. Essentially, 'passive' - primarily explain, rather than guide, occurrence of change.	Include organizational theory [30], behavioural theories, and diffusion of innovation.
(4) Implementation theories;	Adapted from classical theories, specifically for implementation	COM-B (Capability, Opportunity, Motivation and Behaviour) and Normalization Process Theory).
(5) Evaluation frameworks	Developed specifically to inform evaluation of outcomes from complex interventions.	RE-AIM and PRECEDE-PROCEED model

To effectively analyze the factors influencing the adaptability, scalability, and sustainability (ASaS) of complex health interventions (CHIs), this review integrates insights from multiple frameworks. Initially, the EPIS (Exploration, Preparation, Implementation, Sustainment) model was utilized, categorizing influencing factors into four key dimensions: Outer Context, Inner Context, Intervention Characteristics, and Bridging Factors. However, a more comprehensive understanding was needed, as the EPIS model alone did not fully capture the complexity of these factors.

To address this, features from the CFIR (Consolidated Framework for Implementation Research) and insights from the NASSS (Non-adoption, Abandonment, Scale-up, Spread, and Sustainability) framework and the Dynamic Sustainability Framework (DSF) were integrated. This meta-model enhancement involves expanding the descriptions within each EPIS dimension to cover additional critical elements found in these other frameworks.

Specifically, within the Inner Context, the organizational characteristics were elaborated to reflect deeper organizational dynamics affecting CHIs. In the Outer Context, the Sociopolitical Context was added, acknowledging its crucial influence on intervention outcomes. Further, the Intervention Characteristics were detailed more extensively to capture the nuanced nature of the interventions themselves.

This enriched model aims to provide a robust analytical framework that better reflects the complex interplay of factors influencing the ASaS of CHIs. By adopting this meta-model, the study offers a comprehensive theoretical foundation that underpins the examination of these complex interventions, paving the way for more targeted and effective implementation strategies in diverse settings.

Finally, the Theoretical Quality Tool, adapted from Hean et al. [31], was employed to rigorously assess the applicability of the collected (TMFs) in the context of Low- and Middle-Income Countries (LMICs).

## Results

### Characteristics of included studies

**Fig. 1:**
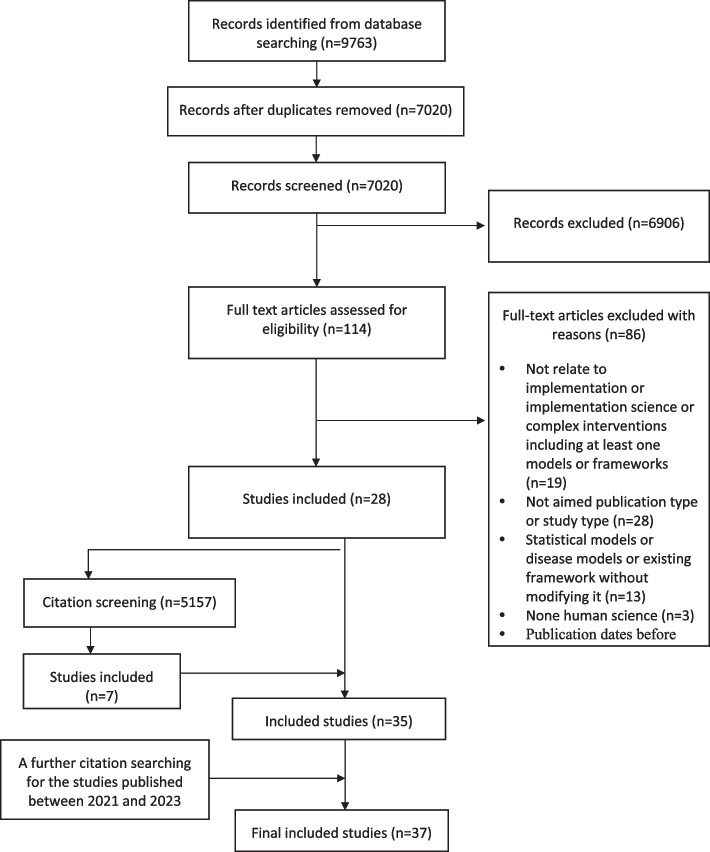
PRIMA diagram of article selection

The flowchart of the search results (Fig. 1) shows that the search identified 9763 studies. Following removal of duplicates and application of eligibility criteria, 37 studies remained for inclusion in the review. 25 studies provide macroscopic TMFs for CHIs [5–9, 30, 32–49] worldwide. A further seven included TMFs [50–56] that were developed in high-income countries and only five studies [21, 57–60] targeted LMICs.

### Types of TMF

Overall, 28 [5–9, 21, 32–46, 48–50, 55, 57, 58, 61] of the 37 studies describe macroscopic TMFs and nine studies [30, 47, 51–54, 56, 59, 60] describe TMFs for specific relevant interventions. Categorising these according to the five categories of Per Nilsen's schema (Table 3) reveals that 14 of the 37 TMFs are process models, 14 are determinant frameworks, one is classic theory, one is implementation theory, and seven are evaluation frameworks (See Additional file 5). One classic theory and one implementation theory are included. The Detailed classification for collected TMFs is described in Table 4.

**Table 4:** Overview of frameworks and models used in the complex health interventions

**Category of the framework**	**Name of the framework (underlying theory if any)**	**Name of the study**	**The correlation with three key concepts (adaptability, scalability and sustainability)**
**Process models**	**EPIS Model**	Advancing a Conceptual Model of Evidence-Based Practice Implementation in Public Service Sectors	Sustainability Fidelity Monitoring and Support Dissemination
		Systematic review of the Exploration, Preparation, Implementation, Sustainment (EPIS) framework	Sustainment Adaptation Diffusion
	**The dynamic sustainability framework (DSF)**	The dynamic sustainability framework: addressing the paradox of sustainment amid ongoing change	Sustainability Adaptation/adaptation phase
	**Overview of phases and steps in the process of adaptation**	Adapting evidence-informed complex population health interventions for new contexts: a systematic review of guidance	Sustainable Adaptability Scalable
	**Stages of innovation implementation and factors affecting sustainability**	A framework for implementing sustainable oral health promotion interventions	Sustainable Diffusion of innovation theory
	**the AIDED model**	A model for scale up of family health innovations in low-income and middle-income settings: A mixed methods study	Sustainability Scaling up Dissemination, diffusion
	**triple C model**	Implementation of sustainable complex interventions in health care services: the triple C model	Sustainability Adaptability
	**The Scaling Up Management (SUM) Framework**	Scaling up—from vision to large-scale change: a management framework for practitioners	Sustaining Adapting Scaling up/expanding
	**PRISM**	A Practical, Robust Implementation and Sustainability Model (PRISM) for Integrating Research Findings into Practice	Sustaining Diffusion
	**IHI Framework for Going to Full Scale**	A framework for scaling up health interventions: lessons from large-scale improvement initiatives in Africa	Scaling up/spread
	**A cross-cultural adaptation framework**	A framework for cross-cultural development and implementation of complex interventions to improve palliative care in nursing homes: the PACE steps to success programme	Sustainability Cross-cultural adaptation
	**WICID framework**	WICID framework version 1.0: criteria and considerations to guide evidence-informed decision-making on non-pharmacological interventions targeting COVID-19	Adaption Expanded
	**Greenhalgh et al.’s diffusion of innovation model**	Explaining high and low performers in complex intervention trials: a new model based on diffusion of innovations theory	Sustainably Adaptation Diffusion, spread
	**Hybrid Framework**	Development and application of a hybrid implementation research framework to understand success in reducing under-5 mortality in Rwanda	Sustainment Adaptation Diffusion
**Determinant frameworks**	**CFIR**	Fostering implementation of health services research findings into practice: a consolidated framework for advancing implementation science (CFIR	Sustainability Adaptation Scaling up, dissemination
	**chronic care models (CCM)**	Factors influencing the implementation of chronic care models: A systematic literature review	Sustainability
	**A Proposed Framework for Success**	Scaling Up Global Health Interventions: A Proposed Framework for Success	
	**Conceptual framework of sustainability of interventions implemented in SSA**	Toward the sustainability of health interventions implemented in sub-Saharan Africa: a systematic review and conceptual framework	Sustainability Local adaptation
	**Conceptual framework for sustainability of public health programs**	An Agenda for Research on the Sustainability of Public Health Programs	Sustainable Adaptation Diffusion
	**Framework of Dissemination in Health Services Intervention Research**	Interventions in Organizational and Community Context: A Framework for Building Evidence on Dissemination and Implementation in Health Services Research	Sustainability Adapting Disseminating
	**A Person-Focused Model of Care**	A Person-Focused Model of Care for the Twenty-First Century: A System-of-Systems Perspective	Sustainable Complex adaptive systems
	**Integrated sustainability framework**	The Sustainability of Evidence-Based Interventions and Practices in Public Health and Health Care	Sustainable Adaptability
	**FRAME**	The FRAME: an expanded framework for reporting adaptations and modifications to evidence-based interventions	Adaptation/fidelity
	**ExpandNet framework**	Practical guidance for scaling up health service innovations. Geneva: World Health Organization	Sustainable Adaptation Scaling up
	**multiple models**	Framework for the establishment of a feasible, tailored and effective perinatal education programme	Sustainability Adaptation Feasibility
	**Conceptual Framework to Prevent Childhood Obesity Through Policy-Level Initiatives in Afterschool Programs**	Translating Policies Into Practice: A Framework to Prevent Childhood Obesity in Afterschool Programs	Adapted from other settings
	**complex adaptive system**	Moving alcohol prevention research forward—Part I: introducing a complex systems paradigm	Adaptation, complex adaptive system
	**Conceptual framework for evaluating the scale-up and sustainability of digital solutions for front-line health workers**	A tale of ‘politics and stars aligning’: analysing the sustainability of scaled up digital tools for front-line health workers in India	Sustainability Adaptability Scalable
**Classic theories**	**Organizational theory**	Organizational theory for dissemination and implementation research	Adaptation, sustainment, dissemination
**Implementation theories**	**NPT theory**	Normalisation process theory: a framework for developing, evaluating and implementing complex interventions	Normalisation Wide scale
**Evaluation frameworks**	**RE-AIM**	Evaluating the public health impact of health promotion interventions: the RE-AIM framework	Adaptation Fidelity, cost
	**Theory of Change (ToC)**	Theory of Change: a theory-driven approach to enhance the Medical Research Council's framework for complex interventions	Sustainable Scalable
	**NASSS Framework**	Beyond Adoption: A New Framework for Theorizing and Evaluating Nonadoption, Abandonment, and Challenges to the Scale-Up, Spread, and Sustainability of Health and Care Technologies	Sustainability Scale-up/spread
	**A comprehensive conceptual framework for implementation science**	Developing a conceptual framework for implementation science to evaluate a nutrition intervention scaled-up in a real-world setting	Sustainability Scaling-up
	**The systems transformation framework (STF)**	The Power of the Frame: Systems Transformation Framework for Health Care Leaders	Sustainable Complex adaptive system
	**the Context and Implementation of Complex Interventions (CICI) framework**	Making sense of complexity in context and implementation: the Context and Implementation of Complex Interventions (CICI) framework	Sustainability Adaption

### Adaptability, scalability and sustainability definitions

All 37 included studies reported at least two out of the three concepts of ASaS, and the specific concepts involved in each study. (See Table 5). Definitions of ASaS found in the included studies are shown in the Additional file 2.

**Table 5:** The terminology used in the collected TMFs

**Concept**	**No. of Studies**	**Terminology**
Adaptability	22/37	adaptability, adaptation, local adaptation, adapting, complex adaptive system, fidelity, and feasibility
Scalability	27/37	scalable, scale up, diffusion, dissemination, spread, expanding
Sustainability	29/37	sustaining, sustainable, sustainability, normalization, sustainably, sustainment, and maintenance.

### The process of implementation and relationships of adaptability, scalability and sustainability

Diverse TMFs support a strong correlation between the three ASaS concepts and the implementation process. Twenty-five of the TMFs explicitly mentioned implementation of CHIs; while the remaining ten did not [6, 8, 21, 39, 40, 44, 54–56, 62].

This review confirms the interconnectedness of adaptability, scalability, and sustainability in the implementation of complex health interventions (CHIs). The findings suggest that adaptability is crucial during the initial stages of CHI deployment, determining the potential for effective and cost-efficient implementation. As the intervention progresses, scalability becomes critical, ensuring that strategies effective on a smaller scale can be expanded to broader populations and regions. Ultimately, sustainability is achieved in the final stages, focusing on maintaining the benefits of the intervention over time and making necessary adjustments based on ongoing feedback and changing conditions.

In essence, the successful scaling and long-term sustainability of CHIs fundamentally depend on their initial adaptability. This streamlined approach highlights the critical progression from adaptability through scalability to sustainability, without delving into the specifics of various models and frameworks.

### Influencing factors of adaptability, scalability and sustainability of complex interventions

This study collected and analyzed factors affecting the adaptability, scalability, and sustainability (ASaS) of complex health interventions (CHIs), systematically categorizing them into four distinct categories: outer context, inner context, intervention characteristics, and bridging factors. This classification helps clarify the various factors that influence the implementation of health interventions.

While all these factors impact the three concepts of ASaS, some have particularly close relationships with specific ASaS concepts. Subsequent sections will explore these factors in detail, emphasizing those closely linked to individual ASaS concepts. This approach highlights the multidimensionality of these factors and their varying impacts on the adaptability, scalability, and sustainability of CHIs. The overview of the factors influencing the ASaS is shown in the Table 6.

**Table 6:** The factors influencing the ASaS of CHIs

**Outer context**	**Inner context**
• Sociopolitical context • Ethical • Legislation • Norms or regulations • Policies • Religion • Interorganizational Networks • Funding • Client Advocacy • Leadership	• Organizational characteristics • Absorptive capacity • Organization readiness • Structure • Values or visions • Working environment • Tension for change • Organization culture • Credibility and reputation • Leadership • Individual adopter or provider characteristics • Ability or capacity • Race • Spoken language • Training or education • Views • Tension for change • Individual culture • Monitoring and evaluation • Staffing
Intervention characteristics • Physical environment • Access to resources • Project champions • Stakeholder Involvement • Communication among healthcare workers and participants • Advanced support systems or tools • Technology advance or environment • Geographical factors • Time-cost	Bridging factors • Community function • Purveyors/Intermediaries

#### Outer context

Name of influencing factors, suggested definition, frequency of influencing factors of Outer context is shown in Table 7.

**Table 7:** Name of influencing factors, suggested definition, frequency of influencing factors of outer context

	**Suggested Definition**	**Frequency of influencing factors**
**OUTER CONTEXT**		
Sociopolitical context	Relating to, or involving a combination of social and political factors [45]. The political context focuses on the distribution of power, assets and interests within a population, as well as the range of organisations involved, their interests and the formal and informal rules that govern interactions between them. Also comprises health care system and its accessibility (e.g., delivery of services, leadership and governance, health information, human resources and financing) [39]	18
Ethical	Reflections of morality, which encompasses norms, rules, standards of conduct and principles that guide the decisions and behaviour of individuals and institutions	2
Legislation	Rules and regulations established to protect a population’s rights and societal interests [63]	8
Norms or regulations	The informal rules that govern behavior in groups and societies; shared standards of acceptable behavior by groups	8
Policies	Incentives (or disincentives) embedded in regulatory policies, funding and reimbursement programs, and rules and policies of adopting organizations themselves that alter the costs and benefits supporting new behaviors and practices. Incentives may be monetary or come in non-financial forms. Also a broad construct that includes external strategies to spread interventions, including policy and regulations (governmental or other central entity), external mandates, recommendations and guidelines, pay-for-performance, collaboratives, and public or benchmark reporting [6]	15
Religion	a social-cultural system of designated behaviours and practices, morals, beliefs, worldviews, texts, sanctified places, prophecies, ethics, or organizations, that generally relates humanity to supernatural, transcendental, and spiritual elements [58]	1
Interorganizational Networks	Includes the linkages and connections among organizations and other stakeholders that enable social support and flows of information within a community or healthcare system [38]	18
Funding	Fiscal support can target multiple levels (e.g., staff training, fidelity monitoring, provision of the innovation) involved in implementation and delivery/use of the innovation [45]	19
Client Advocacy	Support/marketing for system change based on consumer needs, priorities and/or demographics [45].	3
Leadership	Characteristics and behaviors of key decision-makers pertinent at all levels who are necessary but not sufficient to facilitate or promote the implementation process and delivery/use of the innovation [45].	16

##### Sociopolitical context

This study has identified multiple studies highlighting how sociopolitical factors deeply influence the adaptability, scalability, and sustainability (ASaS) of complex health interventions (CHIs) [8, 30, 35, 37, 39, 42, 44, 47, 49–54, 58–60, 64]. These factors, including religion [58], ethics [39, 56], legislation [30, 35, 39, 44, 54, 58, 59, 64], norms or regulations [7, 21, 35, 38, 49, 54, 56, 58], and policies [6, 7, 9, 21, 35, 37–39, 44, 50, 51, 54, 56, 57, 59], play a critical role in shaping health outcomes and addressing healthcare disparities. The influence of sociocultural factors such as common traditions, habits, patterns, and beliefs was also evident across different populations [35, 37–39, 44, 50, 52, 54, 57, 58, 62].

##### Interorganizational networks

Interorganizational networks bridge full-scale relationships across organizations [65], and it was found to significantly enhance the implementation of CHIs, enabling better adaptation to local contexts and sustainability at lower costs through effective resource sharing and communication.

##### Funding

Also, the role of funding was another major factor discussed, highlighting its critical importance for providing necessary resources such as training, materials, and health services [66]. Nineteen of the identified models or frameworks emphasize fiscal support prioritized in implementation [6–9, 21, 30, 34–36, 38, 39, 44, 45, 50, 54, 55, 58, 59, 64].

##### Client advocacy

Three of the 37 studies identify client advocacy as an important influencing factor [9, 45, 50]. During implementation client advocacy assists healthcare workers, participants and their families in navigating the healthcare system [67].

##### Leadership

Finally, 16 of the 37 studies emphasize leadership [6, 21, 30, 35, 36, 40, 42–45, 50, 51, 56–59]. Specific subgroups may offer either approval or conflict. Strong leadership can promote effective use of resources while encouraging personnel to work towards a common goal.

#### Inner context

Name of influencing factors, suggested definition, frequency of influencing factors of Outer context is shown in the Table 8.

**Table 8:** Inner context

	**Suggested Definition**	**Frequency of influencing factors**
**INNER CONTEXT**		
**Organizational characteristics**	Structures or processes that take place and/or exist in organizations that may influence the process of implementation [45]	12
Absorptive capacity	A set of organizational routines and processes by which [organizations] acquire, assimilate, transform, and exploit knowledge to create a dynamic organizational capacity [61]. Absorptive capacity also describes an organizations pre-existing knowledge/skills, ability to use new knowledge, specialization and mechanisms to support knowledge sharing [50]	15
Organization readiness	Relationship between people, processes, systems and performance measurement. It requires synchronization and coordination without which no implementation will be successful	5
Organizational structure	Each department or unit able to make semiautonomous decisions) [61]. Relates to structure and way an organization operates, including differences in mission, size, decision-making process, and services offered. Organizational attributes such as larger size and greater differentiation in personnel and structure are associated with adoption of new organizational forms [38]	11
Values or visions	Encompasses range of attitudes and knowledge about particular health conditions, expectations and priorities toward types of treatments or client populations, and collectively held beliefs and values that may affect the receptivity of individual and organizational stakeholders to adopt or adhere to a new care practice or intervention. Principles of social cognitive, motivation expectancy, and other social learning theories [38]	8
Working environment	organizational climate (shared perceptions of the psychological impact of the work environment on the provider) [50]	10
Tension for change	The degree to which stakeholders perceive the current situation as intolerable or needing change [6]	4
Organization culture	Combines the normative beliefs and shared expectations of the organization [50]	7
Credibility and reputation	the believability of the current intention; reputation is a historical notion based on the sum of the past behaviours [45]	1
**Leadership**	Characteristics and behaviors of key decision-makers pertinent at all levels who are necessary but not sufficient to facilitate or promote the implementation process and delivery/use of the innovation [45]	16
**individual adopter or provider characteristics**	Shared or unique characteristics of individuals (e.g., provider, supervisor, director) that influence the process of implementation [45]	14
Ability or capacity		8
Race		1
Spoken language	Implementers, stakeholders and participants share a common spoken language	2
Training or education		9
Views		8
Tension for change	The degree to which stakeholders perceive the current situation as intolerable or needing change (Damschroder, Aron et al. 2009)	3
Individual culture	characterized by individualism, which is the prioritization or emphasis of the individual over the entire group. In individualistic cultures people are motivated by their own preference and viewpoints. Individualistic cultures focus on abstract thinking, privacy, self- dependence, uniqueness, and personal goals.	4
**Monitoring and evaluation**	Processes or procedures undertaken to ensure adherence to active delivery of the innovation/EBP and/or an implementation strategy.(Moullin, Dickson et al. 2019); Fidelity Support System; Quality assurance evaluation; Continuous quality improvement [45]	8
**Staffing**	Processes or procedures in place at an organization related to the hiring, review, and retention of staff involved in the active delivery of the innovation/EBP and/or its implementation.	14

##### Organizational characteristics

Organizational characteristics influence the process of implementing complex health interventions (CHIs) through structures and processes within organizations. These characteristics encompass ten influencing factors including absorptive capacity [6, 8, 9, 21, 36, 37, 40, 44, 50–52, 56, 57, 59, 64], organizational readiness [8, 30, 50–52], structure [6, 7, 35, 38, 40, 44, 49, 51, 52, 59, 64], values or visions [35, 37, 40, 43, 44, 51, 59], working environment [6–9, 41, 45, 50, 51, 54, 56], tension for change [6, 49, 51, 53], organization culture [6, 35, 40, 43, 50, 58], leadership [6, 21, 35, 36, 40, 42–45, 50, 51, 56–59], credibility and reputation [43]. The adaptability, scalability, and sustainability (ASaS) of CHIs are significantly dependent on these organizational elements.

**Structure**

Organisations with strong organisational power may be likely to implement CHIs because they have stronger leadership and more frequent communication than those with weak or decentralised organisational structures [68].

**Readiness for change**

In addition, strong organisations are prepared and aware of possible encountered changes and can adjust their strategies and approaches of working in time to enable CHIs to be carried out well. Readiness for change is related to other factors including organisational culture, and individual attitudes [69–72].

**Absorptive capacity**

During the exploration and preparation phases of a CHI, an organisation's absorptive capacity (the ability to identify, assimilate, transform, and use external knowledge, research and practice [73]), readiness for change and receptive environment exert a significant impact on the adaptability of CHIs.

**Individual adopter or provider characteristics**

Individual adopter or provider characteristics include participants’ personal characteristics, age, race/ethnicity, education, training, foundation subjects, professional experience, adaptability, personal values and goals, and personal character creative ability.

Fourteen included studies emphasise how the CHI is accepted and scaled-up by participants and health care workers [6–8, 34–36, 41, 45, 47, 51, 53, 54, 56, 58]. In parallel to the organisational level, individual ability or capacity [6, 35, 44, 51, 53, 56, 57, 64], training or education [7, 8, 35, 36, 40, 51, 52, 56], and tenacity for change [6, 51, 53] constitute important factors. CHIs are more easily adapted and diffused when led by experienced and leaders [6, 21, 35, 36, 40, 42–45, 50, 51, 56–59] with common visions or views [6, 35, 51, 53, 56, 60, 64]. In addition, race [35, 50], spoken language [35, 50, 52] and individual culture [35, 53, 56, 59] are considered to be vital influencing factors. Specifically, when there is a high degree of fit between the norms and values of the individual, organisation and CHIs, individuals may find that they achieve higher efficacy when implementing CHIs [64].

**Leadership**

As mentioned above, 16 of the 37 included studies emphasize leadership [6, 21, 30, 35, 36, 40, 42–45, 48, 50, 51, 56–59].

**Staffing**

CHIs require sufficient, well-trained healthcare workers. Fourteen of the 37 studies list staffing as an important factor in their TMFs [7, 30, 34–37, 43–45, 50–52, 56, 64]. Job candidates may be selected so that their knowledge, skills, competencies, and attitudes [74] match the requirements of the CHI.

**Supervision, monitoring and evaluation**

Supervision, monitoring and evaluation refers to the collection, storage, analysis and use of data to assess whether complex interventions are achieving their intended objectives, and further influences improvement, policy development and advocacy of complex intervention [75]. Nine of the 35 studies argue for the vital role of monitoring and evaluation in providing an effective approach by which to assess the effectiveness of complex interventions [21, 30, 40, 43, 44, 50, 51, 58, 64].

#### Intervention characteristics

The characteristics of the intervention itself is also an important factor. Specifically, the physical and community environment, the cost of the intervention and access to resources (8 studies) [6, 21, 30, 34, 38, 51, 52, 56] and the source of funding all exert a direct impact. Project champions are committed to supporting and promoting the implementation of CHI, along with a strong belief in the value of carrying out CHIs [76]. The factors within intervention characteristics is shown in the Table 9.

**Table 9:** Intervention characteristics

	**Suggested Definition**	**Frequency of influencing factors**
**INTERVENTION CHARACTERISTICS**	Factors relating to the characteristics of the innovation to be implemented. Innovation factors can also relate to the relationships of various stakeholders with intervention developers and the flexibility or rigidity in use of the innovation.	7
Communication	effectively communication among healthcare workers and participants	6
Quality and fidelity monitoring/support	continuous data collection plus collection across the sites to promote quality monitoring … costs and its consistency with the initial plan of the project. reflexive monitoring (formal and informal appraisal of the benefits and costs of the intervention).	5
Geographical	circumstances associated with a physical location that affect humans living within a specific area.	5
Project champion	Individuals who dedicate themselves to supporting, marketing, and 'driving through an [implementation]' [6]	11
Resources	Resources dedicated for implementation and on-going operations, including money, training, education, physical space, and time [6]; mobilising community resources.	9
Stakeholder involvement		11
Supervision	the action, process, or occupation of supervising especially : a critical watching and directing (as of activities or a course of action)	6
support system or tool	any hardware, software and other tools and/or utilities used to support complex health interventions. Example: information and communication systems facilitated rather than hindered the implementation and sustainability of a new CCM(Davy, Bleasel et al. 2015)	9
technology advance or environment		8
Time-cost		4

The included frameworks attest to how the characteristics of the CHI decide whether an intervention can be adapted, scaled-up and sustained [6, 8, 33, 36, 47, 51, 53]. Although researchers hope that CHIs can be adapted and conducted as quickly as possible, it takes time for both healthcare providers and participants to adapt to new interventions [77]. Also, when interventions change significantly within a short period of time, the lack of sufficient time to adapt to the intervention and adjust to relevant cultural factors prevent staff and participants from adopting a new CHI [78].

#### Bridging factors

Factors influencing the inter-relationship of outer and inner context are described as “bridging factors” in the EPIS framework. Bridging factors include community engagement and Purveyors/Intermediaries.

Twelve studies stress the importance of the community. Development of complex interventions within a community may be facilitated when they utilise existing community resources, available structures and staff, reducing dependence on external funding [21]. For example, community members were proud to participate in a project to improve malaria prevention through insecticide-treated mosquito nets and thereby contribute to disease control within their community. Consequently, the project was speedily adapted, replicated and scaled-up locally [79]. The community function is also affected by socio-political factors [80]. If the visions and beliefs of the policy are inconsistent with community objectives, the policy hinders spread and sustainability even where the community possesses powerful leadership, project champions and sufficient resources [80, 81].

Purveyors/Intermediaries take on a critical bridging role for key processes in the implementation of CHIs [45]. Purveyors, who may be individuals, groups or communities, aim to facilitate the effective and sustainable implementation of CHIs [82]. Intermediaries provide consultancy and training services to governments, organisations, etc., and also develop and implement different health-services and projects for them [82]. They also provide monitoring, support, quality improvement and evaluation services at the end of the project [82]. The factors within bridging factors is shown in the Table 10.

**Table 10:** Bridging factors

	**Suggested Definition**	**Frequency of influencing factors**
**BRIDGING FACTORS**		
Community engagement	Mobilising community resources [34], community-academic partnerships [45], facilitated community support to meet the needs of patients [34]	13
Purveyors/intermediaries	Individuals, groups or communities, who aim to facilitate the effective and sustainable implementation of CHIs [82]. Intermediaries provide consultancy and training services to governments, organisations, etc., and also develop and implement different health-services and projects for them [82].	1

### Applicability and feasibility of the collected TMFs

This study employs the Theoretical Quality Tool, adapted from Hean et al. [31], to rigorously assess the applicability of various Theoretical Models and Frameworks (TMFs) in the context of Low- and Middle-Income Countries (LMICs). The detailed outcomes of this assessment are presented in the Additional file 6. The summary table highlights the applicability and feasibility of TMFs in LMICs.

Of the 37 TMFs reviewed (two studies identify EPIS), seven demonstrate high applicability and feasibility, readily integrating into LMIC healthcare environments without necessitating significant overhauls. For example, models like the AIDED and NPT are readily implementable in LMICs due to their practicality and context-sensitive design. They integrate seamlessly into existing healthcare systems, offering solutions without the need for extensive system overhauls, crucial in resource-limited environments. Twenty-five TMFs require adaptations to align with the local conditions of LMICs, entailing modifications to fit cultural, economic, and healthcare infrastructure nuances. For example, the EPIS framework, CFIR framework, PRISM Model and Chronic Care Model, though broadly applicable, need customization to fit the unique cultural, economic, and healthcare infrastructures of LMICs.

For the remaining five TMFs, their inherent theoretical complexity and the fact that some were specifically designed for High-Income Countries (HICs) pose significant barriers to adoption in in Low- and Middle-Income Countries (LMICs). This finding highlights an important disconnect between their foundational assumptions and the practical realities of healthcare systems in LMICs. The evaluation outcomes of the applicability and feasibility of the collected 37 TMFs are shown in the Table 11.

**Table 11:** The applicability and feasibility of the collected TMFs

**Highly Applicable and feasible TMFs**	**Name of the TMFs**	**Name of the Study**	**Applicability**	**Feasibility**
1	DSF (Dynamic Sustainability Framework)	The Dynamic Sustainability Framework: Addressing the Paradox of Sustainment Amid Ongoing Change	**Applicabilit**y: This framework (DSF) is potentially suitable for LMICs due to its focus on continuous adaptation and learning, aligning with the diverse healthcare challenges in these regions.	**Feasibility**: DSF's feasibility in LMICs depends on factors such as each region's healthcare infrastructure, cultural factors, and resource availability. Its emphasis on ongoing learning, adaptation, and tailoring interventions to local contexts is critical for addressing specific needs and conditions in LMIC environments.
2	AIDED Model	A model for scale up of family health innovations in low-income and middle-income settings: a mixed methods study	**Applicability**: This model offers a practical approach for scaling up family health innovations in LMICs, focusing on adapting to local contexts and engaging user groups, crucial for addressing the challenges in these regions.	**Feasibility**: It emphasizes developing support systems and effectively spreading innovation, involving essential concepts like assessing the landscape and tailoring innovations to user needs. This approach is designed to overcome barriers to scaling up in LMICs, making it feasible for application in diverse healthcare environments.
3	NPT (Normalization Process Theory)	Normalisation Process Theory: a framework for developing, evaluating and implementing complex interventions	**Applicability**: This Theory (NPT) aids in implementing complex health interventions in LMICs by focusing on their integration into routine practices. It addresses challenges of assimilating these interventions within existing systems and cultural contexts, making it highly applicable in LMICs.	**Feasibility**: NPT concepts such as coherence, cognitive participation, collective action, and reflexive monitoring are crucial for facilitating intervention adoption. These principles enhance the feasibility of NPT in LMICs, considering socio-organizational factors and the need for interventions to resonate with local healthcare environments.
4	A Proposed Framework for Success	Scaling Up Global Health Interventions: A Proposed Framework for Success	**Applicability:** This framework is designed to guide the implementation of new health programs, policies, or interventions in LMICs, considering their unique challenges and requirements, making it suitable for these contexts.	**Feasibility:** A tailored approach for LMICs, drawing on literature and expert interviews, underscores its feasibility in these unique settings. Key aspects enhancing feasibility include simplicity of interventions, local engagement, using state and non-state actors, political will, and incorporating research into implementation, as evidenced in successful health interventions across various LMICs.
5	Theory of Change (ToC)	Theory of Change: a theory-driven approach to enhance the Medical Research Council's framework for complex interventions	**Applicability**: This approach, successfully piloted for mental health projects in LMICs, is adaptable to the varied local conditions in these settings. Its emphasis on stakeholder engagement and identifying causal pathways makes it highly relevant for designing, implementing, and evaluating complex interventions in LMICs.	**Feasibility**: Success of the ToC in LMICs depends on effectively customizing it to local conditions and ensuring active stakeholder participation. While it is adaptable and useful, challenges like significant stakeholder involvement and genuine ownership of ToC maps must be addressed to ensure its feasibility.
6	Conceptual framework of sustainability of interventions implemented in SSA	Toward the sustainability of health interventions implemented in sub-Saharan Africa: a systematic review and conceptual framework	**Applicability**: The study focus on challenges of sustaining health interventions in Sub-Saharan Africa (SSA), emphasizes the importance of sustainability for both communicable and non-communicable diseases in LMICs. It provides insights relevant to regions with similar challenges.	**Feasibility**: While addressing sustainability in SSA, the study faces limitations in resource availability, healthcare workforce, and system strength. Its primary focus on sustainability in SSA may not fully encompass all relevant concepts for broader LMIC contexts, indicating a need for a more comprehensive understanding of LMICs' diverse needs.
7	IHI Framework for Going to Full Scale	A Framework for Scaling Up Health Interventions	**Applicability**: This framework has proven practical in LMICs, particularly in African health initiatives. Its stages effectively address the critical aspects of scaling up health interventions in the resource-varied and infrastructurally challenging contexts of LMICs.	**Feasibility**: Comprehensive coverage of all phases, from initial setup to full-scale implementation and sustainability, demonstrates its feasibility in LMICs, accommodating their diverse healthcare environments and operational challenges.
**TMFs Requiring Adaptation**				
1	PRISM Model	A Practical, Robust Implementation and Sustainability Model (PRISM) for Integrating Research Findings into Practice	**Applicability**: This framework (DSF) is suitable for LMICs due to its emphasis on continuous adaptation and learning. It aligns to the diverse healthcare challenges in LMICs, focusing on adapting and improving health interventions. DSF's adaptability makes it relevant for various regional health concerns.	**Feasibility**: Feasibility in LMICs hinges on factors like healthcare infrastructure, cultural context, and resource availability in each region. Its principles of ongoing learning, adaptation, and fitting interventions to local contexts require consideration of these specific regional needs and conditions.
2	Chronic Care Model (CCM)	Factors influencing the implementation of chronic care models: A systematic literature review	**Applicability**: The model’s effectiveness in LMICs depends on its alignment with the specific health challenges and infrastructures of these regions. While it broadly covers various chronic diseases and settings, indicating a comprehensive approach, its relevance varies based on the unique healthcare contexts in different LMICs.	**Feasibility**: Implementing CCM in LMICs requires meticulous planning and is influenced by factors at multiple healthcare levels. Its adaptability and feasibility in LMICs hinge on the region-specific healthcare needs, infrastructure, and resource capabilities.
3	EPIS Framework	Advancing a Conceptual Model of Evidence-Based Practice Implementation in Public Service Sectors	**Applicability**: The conceptual model is broadly relevant for LMICs, particularly when tailored to address their unique cultural and systemic differences. It encompasses crucial aspects like local needs and the sociopolitical environment, underscoring its adaptability for diverse LMIC settings.	**Feasibility**: The model's feasibility in LMICs involves consideration of local resources and capacities, given its complexity and the multiple phases of exploration, adoption, implementation, and sustainment. Effective application in LMICs demands a deep understanding of local contexts and resource constraints.
4	Triple C Model	Implementation of sustainable complex interventions in health care services: the triple C model	**Applicability**: The model focuses on sustainable complex interventions in healthcare, is well-suited to LMICs, especially when adapted to local contexts. Its stages of consultation, collaboration, and consolidation emphasize key aspects like stakeholder engagement and teamwork, aligning with the needs in LMICs.	**Feasibility**: This model's simplicity and practical approach make it feasible for LMICs, particularly in settings constrained by resources. Its emphasis on clear communication and sustainable practices is critical for the success of healthcare interventions in these resource-limited environments.
5	NASSS Framework	Beyond Adoption: A New Framework for Theorizing and Evaluating Nonadoption, Abandonment, and Challenges to the Scale-Up, Spread, and Sustainability of Health and Care Technologies	**Applicability**: This framework, designed for health and social care technologies, has potential applicability to LMICs. Its broad design covers key areas such as health conditions, technology, adopters, and the wider context, offering concepts likely beneficial for LMICs, especially in informing technology design and implementation planning.	**Feasibility**: Feasibility in LMICs depends on local contexts, resources, and healthcare needs. Its development, informed by empirical case studies and a hermeneutic literature review, makes it adaptable to the diverse settings and challenges characteristic of LMICs.
6	A comprehensive conceptual framework for implementation science	Developing a conceptual framework for implementation science to evaluate a nutrition intervention scaled-up in a real-world setting	**Applicability**: This framework provides well-defined concepts that suggest clarity and potential usability in LMICs. It effectively connects components essential for identifying, scaling up, and sustaining effective interventions, indicating relevance for LMICs.	**Feasibility**: While framework is clear and has proven effective in programs like those in Bangladesh, its adaptation to specific LMIC contexts may require further clarification, ensuring it meets local needs and conditions.
7	EPIS Framework	Systematic review of the Exploration, Preparation, Implementation, Sustainment (EPIS) framework	**Applicability:** Focus on inner and outer context factors, innovation factors, and bridging factors, suggests potential adaptability to diverse settings, including LMICs. Its comprehensive approach to different implementation stages and context factors make it broadly relevant.	**Feasibility:** Feasibility of applying to LMICs depends on the specific contexts and available resources in these regions. While it covers factors crucial for implementing EBPs, the review doesn’t explicitly confirm inclusion of all useful concepts for LMICs, indicating a need for further assessment in these unique settings.
8	The systems transformation framework (STF)	The Power of the Frame: Systems Transformation Framework for Health Care Leaders	**Applicability:** Adaptability and relevance for LMICs, particularly in structuring healthcare leadership and change management, are evident. However, it lacks a specific focus on LMIC contexts, suggesting a need for further contextualization.	**Feasibility:** While concepts are broadly applicable to healthcare systems, their direct relevance and practical implementation in LMICs might require adaptations. This is due to varying healthcare challenges and resource constraints characteristic of LMICs.
9	A Proposed Framework for Success	Scaling Up—From Vision to Large-Scale Change: A Management Framework for Practitioners	**Applicability**: This field-tested framework, applicable across various sectors, aligns well with the challenges of scaling up interventions in LMICs. Its comprehensive approach, encompassing strategic planning, change management, resource allocation, and momentum maintenance, is aptly suited for these regions.	**Feasibility**: Given its proven applicability in different sectors, principles and methodologies show promise for effective implementation in LMICs, considering their specific challenges and needs in healthcare and resource management.
10	CFIR framework	Fostering implementation of health services research findings into practice: a consolidated framework for advancing implementation science	**Applicability**: The CFIR offers a comprehensive and pragmatic approach, making it adaptable for LMICs. Its flexibility and thorough consideration of both internal and external factors in implementation align well with the diverse challenges of health service implementation in LMICs.	**Feasibility**: Includes wide range of relevant concepts for LMICs, including its adaptability to local social, economic, and cultural contexts, supporting its feasibility in these varied settings, guiding effective management of health service implementation processes.
11	the Context and Implementation of Complex Interventions (CICI) framework	Making sense of complexity in context and implementation: the Context and Implementation of Complex Interventions (CICI) framework	**Applicability**: The CICI framework's comprehensive approach and focus on socio-economic and cultural contexts make it suitable for application in LMICs. It addresses a broad spectrum of factors vital for the success of complex interventions in these diverse environments.	**Feasibility**: Given its emphasis on context, the CICI framework is relevant and feasible for LMICs, providing a guide for effectively handling the unique challenges and complexities of health service implementation in these settings.
12	Conceptual framework for sustainability of public health programs	An Agenda for Research on the Sustainability of Public Health Programs	**Applicability**: Suggestions are adaptable for LMICs, focusing on sustainability of health interventions, particularly maintaining benefits post-funding. This aspect is highly relevant for LMICs, where sustaining health initiatives is a critical concern.	**Feasibility**: For effective application in LMICs, must consider specific resource limitations and health system dynamics prevalent in these regions. Comprehensive approach to addressing sustainability highlights feasibility in LMICs, considering their unique healthcare environments.
13	Framework of Dissemination in Health Services Intervention Research	Interventions in Organizational and Community Context: A Framework for Building Evidence on Dissemination and Implementation in Health Services Research	**Applicability**: This framework emphasizes adapting health interventions to diverse community settings in LMICs, focusing on the importance of community-based approaches given the varied resources and cultural contexts. It covers understanding multi-layered community dynamics and the diffusion of new practices.	**Feasibility**: Including essential elements like contextual factors, diffusion stages, and intervention outcomes, tailored to unique challenges in LMICs, making it a feasible approach for health interventions in these regions.
14	A Person-Focused Model of Care	A Person-Focused Model of Care for the Twenty-First Century: A System-of-Systems Perspective	**Applicability**: The model, integrating physical, mental, and social health aspects using a system-of-systems approach, is suitable for LMICs. It addresses multi-morbidity and provides a holistic view of health, essential for LMICs facing complex health challenges.	**Feasibility**: The focus on realigning and integrating existing resources, rather than requiring new infrastructure, makes the model feasible for LMICs. Including key elements like various health dimensions and stakeholder roles, the model effectively caters to the healthcare challenges in these regions.
15	Integrated sustainability framework	The Sustainability of Evidence-Based Interventions and Practices in Public Health and Health Care	**Applicability**: Sustaining EBIs in LMICs necessitates adapting to their specific resources, cultural differences, and economic conditions. This adaptation addresses the challenges of applying interventions initially developed in more resource-rich settings.	**Feasibility**: While concepts like community engagement and cultural adaptability are essential for the success of EBIs in LMICs, sustainability frameworks that highlight these aspects require further exploration. More research is needed to fully grasp their applicability across LMICs' diverse contexts, ensuring interventions are effectively tailored to local needs and realities.
16	RE-AIM Framework	Evaluating the Public Health Impact of Health Promotion Interventions: The RE-AIM Framework	**Applicability**: The RE-AIM framework, focusing on reach, efficacy, adoption, implementation, and maintenance, is adaptable for evaluating public health interventions in LMICs, addressing their effectiveness and sustainability.	**Feasibility**: Adapting to LMICs presents challenges due to resource limitations, cultural differences, and health system disparities. Unique challenges in these regions may require additional considerations beyond the five dimensions, ensuring comprehensive and context-sensitive application.
17	FRAME	The FRAME: an expanded framework for reporting adaptations and modifications to evidence-based interventions	**Applicability**: The FRAME framework, focusing on characterizing modifications to interventions, is suitable for LMICs as it addresses both planned and unplanned changes, critical in their diverse, resource-limited settings. It encompasses a wide range of intervention changes, including proactive adaptations and reactive modifications.	**Feasibility**: While FRAME provides a structure for understanding modifications, LMIC-specific challenges such as infrastructure, resource constraints, and cultural diversity may necessitate further consideration. This involves ensuring modifications align with original intervention goals while being sensitive to local contexts.
18	ExpandNet framework	Practical guidance for scaling up health service innovations	**Applicability**: Focus on addressing technical, managerial, and financial aspects is crucial for LMICs, ensuring that interventions are suited to their specific healthcare contexts and resource limitations.	**Feasibility**: The necessity for additional resources poses a challenge in LMICs, where such resources are often limited. Effectiveness in these settings hinges on practical testing under real-life conditions unique to LMICs, to validate its adaptability and utility.
19	Multiple models	Framework for the establishment of a feasible tailored and effective perinatal education programme	**Applicability**: Focus on adapting antenatal education to the specific needs of women in LMICs, considering local healthcare systems and cultural contexts, makes it highly applicable. It addresses diverse population needs and covers comprehensive maternal and child health aspects, vital in culturally and socioeconomically diverse LMICs.	**Feasibility**: The emphasis on adaptability and relevance enhances feasibility in LMICs. Key concepts like personalized education and community involvement are essential for effective implementation in these regions, where tailored approaches are necessary to meet unique healthcare challenges.
20	A cross-cultural adaptation framework	A framework for cross-cultural development and implementation of complex interventions to improve palliative care in nursing homes: the PACE Steps to Success programme	**Applicability**: The PACE Steps to Success program, aimed at enhancing palliative care in nursing homes, is universally relevant for LMICs. Its comprehensive approach to palliative care is applicable across different cultural contexts, including those in LMICs.	**Feasibility**: Implementing this program in LMICs requires adaptations to align with their unique health and social care systems, legal policies, and cultural norms. Adjustments are necessary to accommodate diverse resource availabilities and healthcare infrastructures, ensuring the program's effectiveness in these varied settings.
21	Greenhalgh et al.’s diffusion of innovation model	Explaining high and low performers in complex intervention trials: a new model based on diffusion of innovations theory	**Applicability**: The model emphasizes the importance of innovation adoption, organizational readiness, leadership, and managerial relations for the success of health interventions in LMICs, highlighting key aspects that are crucial for implementation in these contexts.	**Feasibility**: While these concepts are fundamental, additional context-specific factors may be necessary for LMICs. This includes adapting the model to align with the unique healthcare challenges and varying conditions of these regions, ensuring its practicality and effectiveness in local settings.
22	WICID framework	WICID framework version 1.0: criteria and considerations to guide evidence-informed decision-making on non-pharmacological interventions targeting COVID-19	**Applicability**: This framework, aligned with the WHO-INTEGRATE model, is designed for managing COVID-19 interventions and needs adaptation for LMICs, considering their cultural diversity and health infrastructure.	**Feasibility**: While it addresses health, social, economic, and rights-related aspects of COVID-19 management, customization is necessary for LMICs to address specific challenges like healthcare disparities and economic constraints, due to their unique contexts and resource limitations.
23	Hybrid Framework for Understanding Interventions to Reduce Under-5 Mortality	Development and application of a hybrid implementation research framework to understand success in reducing under-5 mortality in Rwanda	**Applicability**: Tailored for LMICs like Rwanda, offers comprehensive approach to reducing under-5 mortality, adapting existing frameworks with practical implementation suggestions and emphasizing local health system design, leadership, and community involvement.	**Feasibility**: This hybrid framework, focusing on equitable healthcare access and LMIC-specific factors, includes relevant concepts for implementing, adapting, and sustaining health interventions. It addresses contextual factors unique to LMICs, ensuring practicality in these diverse settings.
24	Conceptual framework for evaluating the scale-up and sustainability of digital solutions for front-line health workers	A tale of ‘politics and stars aligning’: analysing the sustainability of scaled up digital tools for front-line health workers in India	**Applicability**: The study on digital health tools in India provides insights applicable to LMICs, focusing on adaptability to local contexts and stakeholder engagement. It underscores the importance of addressing specific challenges like data governance and sustainability in these regions.	**Feasibility**: The scalability and sustainability of such tools in LMICs are contingent on strong government leadership, stakeholder collaboration, and a supportive ecosystem. These factors are crucial for the successful scaling of digital health solutions in LMICs.
**TMFs with Limited Applicability and feasibility**				
1	Complex Population Health Intervention Adjustment	Adapting Evidence-Informed Complex Population Health Interventions for New Contexts	**Applicability**: Emphasizes adapting interventions to new contexts and conserving resources, relevant for LMICs with their complex systems, norms, and structures. Highlights importance of tailoring interventions to specific characteristics and needs of target populations in LMICs.	**Feasibility**: Acknowledges the challenges posed by the complexities in LMICs. While not explicitly focused on LMICs, its principles of adaptation and resource conservation are key to practical implementation in these settings, considering their unique challenges.
2	Sustainable Oral Health Promotion Framework	A framework for implementing sustainable oral health promotion interventions	**Applicability**: Framework for sustainable oral health interventions, encompassing prevention, intervention, and recovery, is adaptable to LMICs. It addresses various stages including training, adoption, implementation, and practice improvement, making it relevant for oral health challenges in these regions.	**Feasibility**: Feasibility in LMICs depends on factors like local resource availability, cultural relevance, and healthcare infrastructure. While comprehensive, specific applicability of its concepts may vary according to each LMIC's unique healthcare challenges and context.
3	Conceptual Framework to Prevent Childhood Obesity Through Policy-Level Initiatives in Afterschool Programs	Translating Policies Into Practice: A Framework to Prevent Childhood Obesity in Afterschool Programs	**Applicability**: Designed for U.S. afterschool programs, focuses on policies and practices relevant to childhood obesity and physical activity but doesn't explicitly address its applicability to LMICs, overlooking factors like resource availability, cultural norms, and economic conditions in these regions.	**Feasibility**: While covering policy implementation, organizational change, and public health, it lacks specific consideration for adapting these concepts to the unique challenges and needs of LMICs, indicating a gap in its feasibility for application in these diverse contexts.
4	Complex adaptive system	Moving alcohol prevention research forward—Part I: Introducing a complex systems paradigm	**Applicability**: Focused on complex systems paradigm in context of U.S. college drinking. Doesn't address applicability to LMICs, neglecting aspects like resource availability, cultural differences, and economic conditions relevant in these regions.	**Feasibility**: While discussing complex systems and computational modeling, does not explore their relevance to LMICs, where context, particularly alcohol misuse and socio-ecological factors, differs significantly. Lack of specific coverage for unique LMIC challenges impacts feasibility of applying these concepts in such settings
5	Organizational theory	Organizational theory for dissemination and implementation research	**Applicability**: Discusses SafeCare's implementation theory in developed contexts, lacking specific guidance for LMICs. Its concepts, tailored for developed countries, may not directly translate to the diverse contexts of LMICs.	**Feasibility**: Focuses on general organizational theories and does not address unique challenges and requirements of LMICs, impacting the direct feasibility of applying these theories in such varied settings.

## Discussion

This theoretical systematic review identified common features and differences across 37 TMFs associated with ASaS.

### Similarities and differences between the TMFs

All identified TMFs emphasize the importance of one or more of the three ASaS concepts. These frameworks aim to enable CHIs to adapt to new contexts and populations, scale up interventions, and ensure long-term effectiveness. The components of different TMFs share broadly similar descriptions, even if the terminology varies. For example, the EPIS framework divides the implementation process into four phases: exploration, preparation, implementation, and sustainment whereas Sarma’s framework [5] describes three domains: i: evidence – efficacy to effectiveness; ii: Scaling-up; and iii: sustainability. A further study [36] describes four stages 1. Training (dissemination); 2. adoption (planning); 3. implementation; 4. practical improvement and two key points (preparation and maintenance).

In the EPIS framework, during exploration and preparation, adaptability is considered to determine whether the complex intervention can be conducted effectively with affordable cost. Domain I of Sarma’s framework [5] includes the four vital components of intervention sources, evidence strength and quality, relative advantages, adaptability and complexity. The Framework - oral health [36] emphasizes adoption within the second stage. Hence, these three stages have the similar key components. The EPIS framework describes how a pilot study is further implemented across diverse participants and areas, which is similar to Domain ii: Scaling-up in Sarma’s framework [5] and the implementation stage in Framework - oral health [36]. Finally, the sustainment stage in EPIS framework, Domain iii: sustainability in Sarma’s framework [5] and the maintenance point in Framework - oral health [36] all convey a shared understanding of sustainability.

Similar stages may be presented in a different order within various models, reflecting the inherently multi-stage and non-linear nature of CHI implementation. Significant differences across different TMFs primarily relate to influences on ASaS. Furthermore, even when different TMFs use the same terminology to describe influencing factors, the meanings may differ due to the inherent complexity and dynamics of these factors.

#### The complexity of influencing factors of adaptability, scalability and sustainability

The TMFs reflect how CHIs and associated influencing factors do not operate in isolation, but are non-linear, interacting and interdependent. Some influencing factors appear across multiple studies. For example, researchers share a consensus about the importance of funding [6–9, 21, 34–36, 38, 39, 44, 45, 50, 54, 55, 58, 64]. Some studies emphasise adequate and sustained financial support from governments and foundations as prerequisite to the sustainability and spread [5, 44, 50, 61], while Sarma [5] recognizes the need to sustain interventions in the absence of adequate funding [21]. In addition, the sociopolitical context, leadership and organizational characteristics are repeatedly mentioned as essential components for implementation. Furthermore, all the factors mentioned in the literature are bi-directional; the same influencing factor may act differently under diverse conditions, either as a facilitator or as a hindrance.

To be specific, first, in terms of the outer context, strong leadership can facilitate effective use of resources while encouraging personnel to work towards a common goal. Also, sociopolitical factors covers ethical considerations [39, 56], legislation [35, 39, 44, 54, 58, 64], norms or regulations [7, 21, 35, 38, 54, 56, 58], policies [6, 7, 9, 21, 35, 37–39, 44, 50, 51, 54, 56, 57], and religion [58]. Legislation and policies not only guide, and often guarantee, complex interventions at the macro level, but also, at the empirical level, provide a basis for adapting CHIs to the local environment thereby making interventions suitable for scale up and long term sustainment [35, 39, 44, 54, 58, 64]. High quality interorganizational communication contributes to the implementation and sustainability of CHIs [83]. Additionally, weak leadership exerts a negative impact on the management of the organisation, funding applications and the recruitment of staff.

Leadership remains an important factor in relation to the inner context. Given that complex interventions are often run by the state, an organisation or a group, strong leadership can facilitate complex interventions. Also, the organisational culture, the vision/belief and the structure of the organisation interact with each factor and are influenced by funding, leadership and staffing.

Strong leadership needs to be accompanied by a structured organisation with a common vision in order to achieve the objectives of complex interventions. People as the carriers of culture, organisation, professional and personal attitudes, norms, interests and affiliations [84] also fulfil an important role. Individual adopter or provider characteristics are important influencing factors. When people within the organisation are aligned with the organisation's philosophy and culture, along with sufficient financial support, strong leadership and effective supervision, adaptation, scale up and long-term sustainment become possible for CHIs. Finally, intervention factors are influenced by both the outer context and the inner context, and bridging factors serve to unite the outer context, the inner context, and the intervention factors.

#### The dynamics of influencing factors of adaptability, scalability and sustainability

Factors that influence complex interventions are dynamic in both temporal and geographical terms. The role of these factors may change over time [85]; anticipated barriers may become facilitators [85]. For example, in the early stages of an intervention, individual adopters may exhibit skepticism and distrust, presenting a barrier to CHI delivery. However, in later stages, if the intervention proves effective, participant attitudes may shift, motivating them to cooperate and thus becoming facilitators. Similarly, in the early stages, newly recruited or local staff may be unfamiliar with the intervention, posing a hindrance. Conversely, as staff become familiar with the intervention, they are better equipped to implement it, thereby becoming facilitators.

Identical influencing factors may have different effects in various geographical and national contexts. For example, women are generally considered a vulnerable group worldwide, particularly in LMICs, where they tend to have lower income and social status compared to men, making it difficult for them to access better health care resources [86]. However, in the matrilineal community in Indonesia, women occupy similar or even higher social status than men, with a cultural tradition of controlling family finances [87]. Therefore, in this context, gender and culture may facilitate interventions, especially maternal and child health related interventions. In relation to funding, reliable sources of funding help to sustain interventions [5], and one of the challenges to sustainability is the lack of long-term available funding [21]. In summary, this systematic review offers a comprehensive understanding of factors influencing ASaS and provides a theoretical framework for effective CHIs in the future.

### Have gaps in knowledge been addressed?

This is the first systematic review of ASaS related TMFs of CHIs. By focusing on the three factors of adaptability, sustainability and spread the review has been able to explore complex interactions of each with each other and with other important factors.

#### How have authors defined scalability, adaptability, and sustainability?

Additional file 2 consolidates definitions of scalability, adaptability and sustainability as identified across the included studies. It is noticeable that “sustainability has evolved from being considered as the endgame of a translational research process to a suggested 'adaptation phase’ that integrates and institutionalizes interventions within local organizational and cultural contexts.” [7]

This literature argues that sustainability is, in fact, a manifestation of adaptability, and that the two concepts are closely related.

#### Which theoretical studies explore at least one of the concepts of scalability, adaptability and sustainability of complex health intervention within a relevant model/frameworks;?

This review reveals the scarcity of theoretical models for LMICs. The review identified four main categories of theoretical models, (i) the generic TMFs (e.g. RE-AIM and CFIR), with no obvious geographical target (26/37); (ii) tailored TMFs developed by some high-income countries (e,g. [52, 53, 56]. for local needs (6/37); (iii) adapted TMFs (e.g. EPIS and Framework of Dissemination in Health Services Intervention Research), originally designed for high-income countries but now adapted to CHIs worldwide; (iv) TMFs specific to low and middle income countries (5/37) (e.g. [21, 57]). 85.7% of the included theories are either generic or specific to high-income countries, with a lack of TMFs specifically targeted at LMICs. As a result of this literature review the team have proceeded to develop a framework for Adaptability, Scalability and Sustainability that is suited for a low- and middle-income country context.

Thirty seven studies explore at least one of the concepts of sustainability, scalability and adaptability. However, no previous studies have explored all three ASaS concepts within a single TMF. Although some studies invoke the need to explore influencing factors and correlation among ASaS, no studies have actually conducted this research.

#### What inter-relationships have been demonstrated between factors influencing scalability, adaptability, and sustainability of the complex health interventions?

The meta-framework provides a comprehensive structure to explore the complexities of CHI implementation, emphasizing the interplay among four critical domains: outer context, inner context, intervention characteristics, and bridging factors.

In the outer context, the interplay between strong leadership, sociopolitical factors, and interorganizational networks is crucial. Strong leadership promotes resource optimization and strategic alignment toward CHI goals, essential for ASaS [35, 39, 44, 54, 58, 64]. Sociopolitical factors, including legislation, policies, and norms, provide a regulatory framework that guides the adaptation of CHIs to local settings, enhancing their feasibility and long-term integration [83]. Additionally, robust interorganizational communication facilitates effective adaptation of CHIs to local contexts, potentially lowering costs and enhancing sustainability.

Within the inner context, organizational culture, structure, and leadership significantly interact, affecting CHI outcomes. Strong, visionary leadership is crucial for fostering an organizational culture that supports CHIs and aligns with broader intervention goals [84]. The organization's structure further influences the implementation of these interventions, with well-structured organizations likely to achieve better scalability and sustainability. Additionally, the characteristics of individual providers and adopters play a critical role, impacting their ability to effectively implement and sustain CHIs.

The characteristics of the intervention itself directly impact its implementation. Factors such as the intervention's complexity, cost, resource requirements, and specific design elements determine the ASaS especially for the stages of adaptability and scalability [6, 21, 30, 34, 38, 51, 52, 56, 59]. Support from project champions and stakeholder involvement are crucial in facilitating the implementation process, ensuring that the interventions are well-supported and aligned with stakeholder expectations [8, 21, 34, 36, 37, 40, 44, 49, 50, 56, 60].

Bridging factors like community engagement and the role of purveyors/intermediaries are vital for linking the outer and inner contexts of CHIs. Community engagement leverages local resources and capacities, which is essential for the localized adaptation and sustainability of interventions [8, 9, 21, 30, 34, 38, 42, 44, 45, 54, 56, 58]. Purveyors and intermediaries facilitate the transfer of knowledge and best practices, enhancing the overall effectiveness and reach of CHIs [45]. These bridging roles ensure that interventions are not only well-integrated within communities but also maintain fidelity to their objectives and outcomes over time.

#### Lack of TMFs designed for LMICs

The lack of specifically designed TMFs for LMICs presents significant challenges in effectively implementing complex health interventions (CHIs) in these settings. Evaluating existing TMFs reveals a gap in their suitability and feasibility for application within the unique healthcare environments of LMICs.

Of the 37 TMFs assessed, many were found to require adaptations to align with the local conditions of LMICs, necessitating modifications to fit cultural, economic, and healthcare infrastructure nuances. For instance, frameworks such as EPIS, CFIR, PRISM Model, and Chronic Care Model, though broadly applicable, need customization to fit the unique contexts of LMICs.

Five of the TMFs reviewed were identified as inherently complex and primarily designed for high-income settings, posing substantial barriers to their adoption in LMICs. This highlights a critical disconnect between the foundational assumptions of these models and the practical realities of healthcare systems in LMICs, which face challenges such as limited resources, differing disease burdens, and varied healthcare delivery systems.

Despite these challenges, some models demonstrate higher applicability and feasibility. For example, the Dynamic Sustainability Framework (DSF) and the AIDED model are noted for their practicality and context-sensitive design, aligning with the continuous adaptation and learning required in LMICs. These models integrate seamlessly into existing healthcare systems, offering solutions without the need for extensive system overhauls, which is crucial in resource-limited environments.

The findings underscore the need to develop or adapt existing TMFs specifically tailored to the conditions of LMICs. This involves considering local healthcare practices, resource limitations, and cultural factors to ensure that the frameworks are both applicable and feasible in supporting the effective implementation and sustainability of CHIs in these settings.

### Strengths and limitations

This systematic review retrieved relevant literature through a comprehensive search across four databases. Only studies published in English were included, potentially missing those from the grey literature. Identifying relevant implementation TMFs proved challenging due to the complex and diffuse terminologies used in this field. Exhaustive lists of synonyms would have been prohibitive, resulting in lack of specificity and numerous false positives. The authors sought an optimal balance between sensitivity and workload. Although the included studies were evaluated using a quality assessment tool, the risk of bias remains, particularly since only one author was responsible for data extraction.

Furthermore, although this review has identified how influencing factors interact, no clear theoretical model charts the specific TMFs, routes, and pathways from the influencing factors to the ASaS of CHIs. Finally, concepts such as acceptability, fidelity, and feasibility, are recognized as important features of CHIs [88] but fell outside the remit of this review.

Only one classic theory and one implementation theory are included. There are two possible reasons. Classical theories are borrowed from such disciplines as psychology, sociology and organisational development (e.g. the Diffusion of Innovation theory [89]. Similarly. the Health Belief Model was published in 1950 [90] and the Theory of Planned Behavior in the late 1980s [91]. Given that inclusion requires publication after 2000, many classic theories predate the study period. On the other hand, other theories, such as the implementation climate theory [92], may not be conceptually related to ASaS, resulting in their exclusion. The Detailed classification for collected TMFs is described in Table 4.

## Conclusion

This review synthesizes 37 TMFs that document factors influencing the ASaS of CHIs. It confirms the wide variety of definitions used for adaptability, scalability, and sustainability within current TMFs, which typically do not include all three components. Current approaches focus on high-income countries or generic “whole world” approaches, with few frameworks specific to low- and middle-income countries. Numerous attempts have been made to describe and explore the interrelationships between implementation components. Of these, the EPIS and CFIR frameworks seem to possess the greatest inherent value, particularly within a model consisting of outer context, inner context, intervention characteristics, and bridging factors. This review offers a starting point for further exploration of adaptability, scalability, and sustainability, particularly within a low- and middle-income context.

### Supplementary Information

Supplementary Material 1.

Supplementary Material 2.

Supplementary Material 3.

Supplementary Material 4.

Supplementary Material 5.

Supplementary Material 6.

Supplementary Material 7.

## Data Availability

All data cited in this review derives from published papers and therefore already available.

## Abbreviations

ASaS: Adaptability, scalability and sustainability
COM-B: Capability, Opportunity, Motivation and Behaviour
CHI: Complex health interventions
EBP: Evidence-based practice
EPIS framework: Exploration, Preparation, Implementation, Sustainment (EPIS) framework
LMICs: Low- and middle- income countries
MRC: Medical Research Council
TMF: Theory, model and framework
