# Supplementary material for: Adaptability, Scalability and Sustainability (ASaS) of complex health interventions: a systematic review of theories, models and frameworks
Source: Implement Sci. 2024 Jul 17;19:52. doi: 10.1186/s13012-024-01375-7 (PMC11253497; doi:10.1186/s13012-024-01375-7)
Supplement: Supplementary file 1 — Supplementary Material 1. [file 13012_2024_1375_MOESM1_ESM.docx]

# Additional file 1: The specific search terms and strategy of systematic review of TMFs

- (Sustainable OR Sustainability OR Capacity* building OR Spread OR diffusion)
- (Adaptable OR Adaptability OR Replicabilit* OR Fidelit* OR Adoption)
- (Scalability OR Scalable OR Scaling up OR Scale out OR expansion)
- (Sustainable OR Sustainability OR Capacity* building OR Spread OR diffusion) AND (Adaptable OR Adaptability OR Replicabilit* OR Fidelit* OR Adoption)
- (Sustainable OR Sustainability OR Capacity* building OR Spread OR diffusion) AND (Scalability OR Scalable OR Scaling up OR Scale out OR expansion)
- (Adaptable OR Adaptability OR Replicabilit* OR Fidelit* OR Adoption) AND (Scalability OR Scalable OR Scaling up OR Scale out OR expansion)
- (Sustainable OR Sustainability OR Capacity* building OR Spread OR diffusion) AND (Adaptable OR Adaptability OR Replicabilit* OR Fidelit* OR Adoption) AND (Scalability OR Scalable OR Scaling up OR Scale out OR expansion)

All previous mentioned search terms were combined with the following two sets of terms using the Boolean operator “AND”.

AND (complex NEAR3 intervention* OR complex NEAR3 treatment* OR complex NEAR3 training OR complex NEAR3 system* OR adaptive system*)

AND (Theor* OR models* OR Framework* OR Heuristic* OR Lens OR Paradigm*)
