# Supplementary material for: Adaptability, Scalability and Sustainability (ASaS) of complex health interventions: a systematic review of theories, models and frameworks
Source: Implement Sci. 2024 Jul 17;19:52. doi: 10.1186/s13012-024-01375-7 (PMC11253497; doi:10.1186/s13012-024-01375-7)
Supplement: Supplementary file 2 — Supplementary Material 2. [file 13012_2024_1375_MOESM2_ESM.docx]

# Additional file 2: The inclusive definitions of ASaS

| **Concepts** | **Definitions** |
| --- | --- |
| Adaptability | Adaptability in health care means being skilled at integrating a patient's cultural values or beliefs into any encounter with the awareness and ability to adapt behaviours to maximize the patient’s comfort, reconcile misunderstandings, and be responsive to the patient’s needs. [102] [103] |
| Scalability | deliberate efforts to increase the impact of health service innovations successfully tested in pilot or experimental projects so as to benefit more people and to foster policy and programme development on a lasting basis[48] |
| Sustainability | continued benefits to those who received health services when the program started and to new participants when the supporting funds are discontinued; (2) continued implementation of a program activities in an organization following the discontinuation of the program financial support; and (3) community empowerment to improve their health by continuing the activities of a finished program. Together, these measures, they argue, allow one to plan for “what is to be sustained, how or by whom, how much and by when” [104] |
