# Supplementary material for: Adaptability, Scalability and Sustainability (ASaS) of complex health interventions: a systematic review of theories, models and frameworks
Source: Implement Sci. 2024 Jul 17;19:52. doi: 10.1186/s13012-024-01375-7 (PMC11253497; doi:10.1186/s13012-024-01375-7)
Supplement: Supplementary file 3 — Supplementary Material 3. [file 13012_2024_1375_MOESM3_ESM.docx]

# Additional file 3: The factors influencing the adaptability, scalability and sustainability of complex interventions in the included studies of theoretical systematic review

|  | **Influencing factors** |  | **Referred studies** |
| --- | --- | --- | --- |
|  | **Main themes** | **sub-components** |  |
| **outer context** | **Sociopolitical context** |  | 6,7,8,9,10,11,18,24,26,27,28,29,30,31,33,35,36,37 |
|  |  | ethical | 18,32 |
|  |  | legislation | 6,11,18,24,26,33,35,36 |
|  |  | norms or regulations | 4,6,7,13,22,26,32,33 |
|  |  | policies | 4,8,10,13,17,18,19,21,22,24,26,30,32,33,36 |
|  |  | religion | 6 |
|  |  |  |  |
|  | **leadership** |  | 5,6,8,9,13,14,15,16,17,21,24,26,30,32,35,36 |
|  |  |  |  |
|  | **funding** |  | 3,4,5,6,8,11,13,14,17,18,19,22,23,24,26,27,33,35,36 |
|  |  |  |  |
|  | **Client Advocacy** |  | 8,14,19 |
|  |  |  |  |
|  | **Interorganizational Networks** |  | 3,8,9,10,11,14,15,17,19,22,23,24,29,30,32,35,36,37 |
|  |  |  |  |
| **inner context** |  |  |  |
|  | **Organizational characteristics** |  | 1,4,7,8,11,15,19,27,29,30,33,35 |
|  |  | Absorptive capacity | 5,8,10,11,13,15,17,19,21,24,27,29,30,32,36 |
|  |  | Organization readiness | 8,27,29,30,35 |
|  |  | structure | 4,7,11,15,17,22,24,26,29,30,36 |
|  |  | values or visions | 10,15,16,24,26,30,35,36 |
|  |  | working environment | 1,4,8,14,17,19,27,30,32,33 |
|  |  | tension for change | 7,17,28,30 |
|  |  | Organization culture | 6,8,15,16,17,26,35 |
|  |  | Credibility and reputation | 16 |
|  |  |  |  |
|  | **leadership** |  | 5,6,8,9,13,14,15,16,17,21,24,26,30,32,35,36 |
|  |  |  |  |
|  | **Individual adopter or provider characteristics** |  | 1,3,4,5,6,14,17,26,27,28,30,31,32,33 |
|  |  | ability or capacity | 11,17,21,24,26,28,30,32 |
|  |  | race | 26 |
|  |  | spoken language | 26,29 |
|  |  | training or education | 4,5,15,26,27,29,30,32,35 |
|  |  | views | 8,11,17,26,28,30,32,37 |
|  |  | tension for change | 17,28,30 |
|  |  | individual culture | 26,28,32,36 |
|  |  |  |  |
|  | **monitoring and evaluation** |  | 6,8,11,13,15,16,24,30 |
|  |  |  |  |
|  | **staffing** |  | 3,4,5,8,10,11,14,16,24,26,29,30,32,35 |
| **intervention characteristics** |  |  | 5,17,25,27,28,30,31 |
|  |  | communication | 7,10,14,24,27,30 |
|  |  | Quality and fidelity monitoring/support | 2,5,14,24,35 |
|  |  | geographical | 5,18,32,33,35 |
|  |  | project champion | 3,5,7,8,10,13,15,24,27,32,37 |
|  |  | resources | 3,13,17,22,29,30,32,35,36 |
|  |  | stakeholders involvement | 2,6,8,9,13,14,23,30,32,36,37 |
|  |  | supervision | 4,13,16,24,29,32 |
|  |  | support system or tool | 4,5,8,10,14,21,27,33,35 |
|  |  | technology advance or environment | 6,11,24,26,27,30,32,35 |
|  |  | Time | 3,7,26,35 |
|  |  |  |  |
| **Bridging factors** | **community engagement** |  | 3,6,9,13,14,19,22,24,27,32,33,35,36 |
|  |  |  |  |
|  | **Purveyors/intermediaries** |  | 14 |

**The study name of the referred study number**

1. A Practical, Robust Implementation and Sustainability Model (PRISM) for Integrating Research Findings into Practice
2. Adapting evidence-informed complex population health interventions for new contexts: a systematic review of guidance
3. Factors influencing the implementation of chronic care models: A systematic literature review
4. The dynamic sustainability framework: addressing the paradox of sustainment amid ongoing change
5. A framework for implementing sustainable oral health promotion interventions
6. A model for scale up of family health innovations in low-income and middle-income settings: A mixed methods study
7. Normalisation process theory: a framework for developing, evaluating and implementing complex interventions
8. Advancing a Conceptual Model of Evidence-Based Practice Implementation in Public Service Sectors
9. Scaling Up Global Health Interventions: A Proposed Framework for Success
10. Implementation of sustainable complex interventions in health care services: the triple C model
11. Beyond Adoption: A New Framework for Theorizing and Evaluating Nonadoption, Abandonment, and Challenges to the Scale-Up, Spread, and Sustainability of Health and Care Technologies
12. Developing a conceptual framework for implementation science to evaluate a nutrition intervention scaled-up in a real-world setting
13. Toward the sustainability of health interventions implemented in sub-Saharan Africa: a systematic review and conceptual framework
14. Systematic review of the Exploration, Preparation, Implementation, Sustainment (EPIS) framework
15. The Power of the Frame : Systems Transformation Framework for Health Care Leaders
16. Scaling up—from vision to large-scale change: a management framework for practitioners
17. Fostering implementation of health services research findings into practice: a consolidated framework for advancing implementation science (CFIR)
18. Making sense of complexity in context and implementation: the Context and Implementation of Complex Interventions (CICI) framework
19. An Agenda for Research on the Sustainability of Public Health Programs
20. Theory of Change: a theory-driven approach to enhance the Medical Research Council's framework for complex interventions
21. A framework for scaling up health interventions: lessons from large-scale improvement initiatives in Africa
22. Interventions in Organizational and Community Context: A Framework for Building Evidence on Dissemination and Implementation in Health Services Research
23. A Person-Focused Model of Care for the Twenty-First Century: A System-of-Systems Perspective
24. The Sustainability of Evidence-Based Interventions and Practices in Public Health and Health Care
25. Evaluating the public health impact of health promotion interventions: the RE-AIM framework
26. The RE-AIM framework: a systematic review of use over time
27. The FRAME: an expanded framework for reporting adaptations and modifications to evidence-based interventions
28. Practical guidance for scaling up health service innovations. Geneva: World Health Organization
29. Framework for the establishment of a feasible, tailored and effective perinatal education programme
30. A framework for cross-cultural development and implementation of complex interventions to improve palliative care in nursing homes: the PACE steps to success programme
31. Explaining high and low performers in complex intervention trials: a new model based on diffusion of innovations theory
32. WICID framework version 1.0: criteria and considerations to guide evidence-informed decision-making on non-pharmacological interventions targeting COVID-19
33. Translating Policies Into Practice: A Framework to Prevent Childhood Obesity in Afterschool Programs
34. Moving alcohol prevention research forward—Part I: introducing a complex systems paradigm
35. Organizational theory for dissemination and implementation research

**Further studies obtained from citation searching**

1. Development and application of a hybrid implementation research framework to understand success in reducing under-5 mortality in Rwanda
2. A tale of ‘politics and stars aligning’: analysing the sustainability of scaled up digital tools for front-line health workers in India
