# Supplementary material for: Adaptability, Scalability and Sustainability (ASaS) of complex health interventions: a systematic review of theories, models and frameworks
Source: Implement Sci. 2024 Jul 17;19:52. doi: 10.1186/s13012-024-01375-7 (PMC11253497; doi:10.1186/s13012-024-01375-7)
Supplement: Supplementary file 4 — Supplementary Material 4. [file 13012_2024_1375_MOESM4_ESM.docx]

# Additional file 4: The quality assessment of the collected TMFs (Adapted from Hean et al, 2016)

| **N** | **Author (year)** | **Name of the study** | **1) Is the methodology identified and justified?** | **2) Was a theoretical lens or perspective used to guide the study, with a reference provided?** | **3) Is the theoretical framework described?** | **4) Is the theoretical framework easily linked with the problem?** | **5) If a conceptual framework is used, are the concepts adequately defined?** | **6) Are the relationships among concepts clearly identified?** | **7) Are the influencing factors of concepts clearly identified?** | **8)Are the relationships among influencing factors clearly described?** |
| --- | --- | --- | --- | --- | --- | --- | --- | --- | --- | --- |
| 1 | AC Feldstein, 2008 | A Practical, Robust Implementation and Sustainability Model (PRISM) for Integrating Research Findings into Practice | √ | √ | √ | √ | √ | √ | √ | × |
| 2 | A Movsisyan, 2019 | Adapting evidence-informed complex population health interventions for new contexts: a systematic review of guidance | √ | √ | √ | √ | √ | √ | √ | √ |
| 3 | C Davy, 2015 | Factors influencing the implementation of chronic care models: A systematic literature review | √ | √ | √ | √ | √ | √ | √ | √ |
| 4 | DA Chambers,2013 | The dynamic sustainability framework: addressing the paradox of sustainment amid ongoing change | √ | √ | √ | √ | √ | √ | √ | √ |
| 5 | DD Simpson, 2011 | A framework for implementing sustainable oral health promotion interventions | √ | √ | √ | √ | √ | √ | √ | × |
| 6 | EH Bradley, 2012 | A model for scale up of family health innovations in low-income and middle-income settings: A mixed methods study | √ | √ | √ | √ | √ | √ | √ | √ |
| 7 | Murray., 2010 | Normalisation process theory: a framework for developing, evaluating and implementing complex interventions | √ | √ | √ | √ | √ | √ | √ | √ |
| 8 | GA Aarons,，2010 | Advancing a Conceptual Model of Evidence-Based Practice Implementation in Public Service Sectors | √ | √ | √ | √ | √ | √ | √ | √ |
| 9 | G Yamey, 2012 | Scaling Up Global Health Interventions: A Proposed Framework for Success | √ | √ | √ | √ | √ | √ | √ | √ |
| 10 | H Khalil, 2021 | Implementation of sustainable complex interventions in health care services: the triple C model | √ | √ | √ | √ | √ | √ | √ | × |
| 11 | Greenhalgh, Trisha., 2017 | Beyond Adoption: A New Framework for Theorizing and Evaluating Nonadoption, Abandonment, and Challenges to the Scale-Up, Spread, and Sustainability of Health and Care Technologies | √ | √ | √ | √ | √ | √ | √ | √ |
| 12 | H Sarma, 2021 | Developing a conceptual framework for implementation science to evaluate a nutrition intervention scaled-up in a real-world setting | √ | √ | √ | √ | √ | √ | √ | × |
| 13 | J Iwelunmor, 2015 | Toward the sustainability of health interventions implemented in sub-Saharan Africa: a systematic review and conceptual framework | √ | √ | √ | √ | √ | √ | √ | √ |
| 14 | JC Moullin, 2019 | Systematic review of the Exploration, Preparation, Implementation, Sustainment (EPIS) framework | √ | √ | √ | √ | √ | √ | √ | √ |
| 15 | Kathy A Scott, 2018 | The Power of the Frame : Systems Transformation Framework for Health Care Leaders | √ | √ | √ | √ | √ | √ | √ | √ |
| 16 | L Cooley, 2006 | Scaling up—from vision to large-scale change: a management framework for practitioners | √ | √ | √ | √ | √ | √ | √ | √ |
| 17 | LJ Damschroder, 2009 | Fostering implementation of health services research findings into practice: a consolidated framework for advancing implementation science（CFIR） | √ | √ | √ | √ | √ | √ | √ | √ |
| 18 | Lisa M. Pfadenhauer, 2017 | Making sense of complexity in context and implementation: the Context and Implementation of Complex Interventions (CICI) framework | √ | √ | √ | √ | √ | √ | √ | √ |
| 19 | MA Scheirer, 2011 | An Agenda for Research on the Sustainability of Public Health Programs | √ | √ | √ | √ | √ | √ | √ | √ |
| 20 | MJ De Silva, 2014 | Theory of Change: a theory-driven approach to enhance the Medical Research Council's framework for complex interventions | √ | √ | √ | √ | √ | √ | × | × |
| 21 | PM Barker, 2016 | A framework for scaling up health interventions: lessons from large-scale improvement initiatives in Africa | √ | √ | √ | √ | √ | √ | √ | √ |
| 22 | P Mendel, 2008 | Interventions in Organizational and Community Context: A Framework for Building Evidence on Dissemination and Implementation in Health Services Research | √ | √ | √ | √ | √ | √ | √ | √ |
| 23 | RA Greene, 2014 | A Person-Focused Model of Care for the Twenty-First Century: A System-of-Systems Perspective | √ | √ | √ | √ | √ | √ | × | × |
| 24 | RC Shelton, 2018 | The Sustainability of Evidence-Based Interventions and Practices in Public Health and Health Care | √ | √ | √ | √ | √ | √ | √ | √ |
| 25 | RE Glasgow, 1999 | Evaluating the public health impact of health promotion interventions: the RE-AIM framework | √ | √ | √ | √ | √ | √ | × | × |
| 26 | B Gaglio, 2013 | The RE-AIM framework: a systematic review of use over time | √ | √ | √ | √ | √ | √ | × | × |
| 27 | SW Stirman, 2019 | The FRAME: an expanded framework for reporting adaptations and modifications to evidence-based interventions | √ | √ | √ | √ | √ | √ | √ | √ |
| 28 | WHO， 2009 | Practical guidance for scaling up health service innovations. Geneva: World Health Organization | √ | √ | √ | √ | √ | √ | √ | √ |
| 29 | I Artieta-Pinedo, 2017 | Framework for the establishment of a feasible, tailored and effective perinatal education programme | √ | √ | √ | √ | √ | √ | √ | √ |
| 30 | J Hockley, 2019 | A framework for cross-cultural development and implementation of complex interventions to improve palliative care in nursing homes: the PACE steps to success programme | √ | √ | √ | √ | √ | √ | × | × |
| 31 | H McMullen, 2015 | Explaining high and low performers in complex intervention trials: a new model based on diffusion of innovations theory | √ | √ | √ | √ | √ | √ | √ | √ |
| 32 | JM Stratil, 2020 | WICID framework version 1.0: criteria and considerations to guide evidence-informed decision-making on non-pharmacological interventions targeting COVID-19 | √ | √ | √ | √ | √ | √ | × | × |
| 33 | MW Beets,2013 | Translating Policies Into Practice: A Framework to Prevent Childhood Obesity in Afterschool Programs | √ | √ | √ | √ | √ | √ | √ | √ |
| 34 | Y Apostolopoulos, 2018 | Moving alcohol prevention research forward—Part I: introducing a complex systems paradigm | √ | √ | √ | √ | √ | √ | √ | √ |
| 35 | Birken, 2017 | Organizational theory for dissemination and implementation research | √ | √ | √ | √ | √ | √ | √ | √ |
| **Studies obtained from the citation searching** | | | | | | | | | | |
| 36 | A. Milat, K, 2020 | Development and application of a hybrid implementation research framework to understand success in reducing under-5 mortality in Rwandamakers and implementers | √ | √ | √ | √ | √ | √ | √ | √ |
| 37 | N. S. Singh, 2021 | A tale of ‘politics and stars aligning’: analysing the sustainability of scaled up digital tools for front-line health workers in India | √ | √ | √ | √ | √ | √ | √ | √ |
