# Supplementary material for: Adaptability, Scalability and Sustainability (ASaS) of complex health interventions: a systematic review of theories, models and frameworks
Source: Implement Sci. 2024 Jul 17;19:52. doi: 10.1186/s13012-024-01375-7 (PMC11253497; doi:10.1186/s13012-024-01375-7)
Supplement: Supplementary file 5 — Supplementary Material 5. [file 13012_2024_1375_MOESM5_ESM.docx]

# Additional file 5: the basic information of included studies

| **N** | **Author (year)** | **Name of the study** | **Name of the framework** | **Country** | **Method** | **Reference models** | **Purpose of the framework** | **Key concepts within the TMFs** |
| --- | --- | --- | --- | --- | --- | --- | --- | --- |
| 1 | AC Feldstein, 2008 | A Practical, Robust Implementation and Sustainability Model (PRISM) for Integrating Research Findings into Practice | PRISM—the Practical, Robust Implementation and Sustainability Model. | worldwide | Descriptive | CCM（chronic care model，Model for Improvement；RE-AIM | To provide a new tool for researchers and health care decision makers that integrates existing concepts relevant to translating research into practice. | (PARIHS) framework, which focuses on three elements: evidence, context, and facilitation |
| 2 | Movsisyan, 2019 | Adapting evidence-informed complex population health interventions for new contexts: a systematic review of guidance | Overview of phases and steps in the process of adaptation | worldwide | Systematic review | n/a | To provide information of the establishment of general guidelines on the adaptation of health interventions for complex populations | Adaptability  Drift  Fidelity  Replicability  Scalability  Social validity |
| 3 | C Davy, 2015 | Factors influencing the implementation of chronic care models: A systematic literature review | chronic care models | worldwide | systematic review | n/a | To explore the influencing factors of the implementation of CCMs in primary care settings | 1.acceptability of CCM  2. CCM funding, mental health, teamwork support the healthcare workers  3.supporting patients Encouraging patients, self-management, timely reporting, etc.  4. Prepared resources for interventions to sustain |
| 4 | DA Chambers,2013 | The dynamic sustainability framework: addressing the paradox of sustainment amid ongoing change | The dynamic sustainability framework (DSF) | worldwide | literature review | multiple models | To develop an iterative, dynamic framework named ‘Dynamic Sustainability Framework’ (DSF) to explore the sustainability related concepts in complex interventions | 1. intervention/context  2. practice setting (e.g. clinical or community setting)  3. ecological system (external facilitator for sustainability) |
| 5 | DD Simpson, 2011 | A framework for implementing sustainable oral health promotion interventions | Stages of innovation implementation and factors affecting sustainability | worldwide | Descriptive | PRECEDE-PROCEED planning model, DSF model | To explore fundamental procedural and assessment concepts relevant to the implementation of sustainable behavioral and social interventions in oral health | 1.training  2.adoption, 3.implementation  4.practice |
| 6 | EH Bradley, 2012 | A model for scale up of family health innovations in low-income and middle-income settings: A mixed methods study | the AIDED model | LMICs | A mixed methods study | multiple models | To build an integrated and practical model of scale up that synthesises experiences of family health programmes in low-income and middle-income countries (LMICs). | The AIDED model comprised 5 different but intercorrelated parts:  1. assess the landscape,  2. innovate to fit user receptivity,  3. develop support,  4. engage with user groups 5. devolve efforts for spreading the innovation. |
| 7 | Murray., 2010 | Normalisation process theory: a framework for developing, evaluating and implementing complex interventions | NPT theory | worldwide | descriptive | MRC framework | The develop a theory indicating the factors are required for successful implementation and integration of interventions into routine work (normalisation) | 1.Coherence  2.Cognitive participation  3.Collective action  4.Reflexive monitoring |
| 8 | GA Aarons,，2010 | Advancing a Conceptual Model of Evidence-Based Practice Implementation in Public Service Sectors | EPIS model | used in US previously and now used worldwide | literature review | multiple models | Proposes a multi-level, four phase model of the implementation process (i.e., Exploration, Adoption/Preparation, Implementation, Sustainment), derived from extant literature, and applies it to public sector services. | 1. Exploration Phase 2.Adoption Decision/Preparation Phase 3. Active Implementation Phase 4. Sustainment Phase |
| 9 | G Yamey, 2011 | Scaling Up Global Health Interventions: A Proposed Framework for Success | A Proposed Framework for Success | worldwide | literature review | n/a | This framework is aimed at planners of scale-up processes to use in thinking about strategies for implementing a new program, policy, or intervention to scale. | • attributes of the specific tool or service being scaled up • attributes of the implementers • the chosen delivery strategy • attributes of the “adopting” community • the socio-political context, and Political will and national policies • the research context |
| 10 | H Khalil, 2021 | Implementation of sustainable complex interventions in health care services: the triple C model | triple C model | worldwide | literature review | ACE (Academic Center for Evidence Based Practice) Star Model of Knowledge Transformation, the Knowledge to Action Framework, the Iowa Model, the Ottawa Model for Research Use and the Joanna Briggs Institute (JBI) model, CFIR, RE-AIM | The framework was used to devise the components of the model that explains the successful implementation of complex interventions in healthcare | The consultation stage The collaboration stage The consolidation stage |
| 11 | Greenhalgh, Trisha., 2017 | Beyond Adoption: A New Framework for Theorizing and Evaluating Nonadoption, Abandonment, and Challenges to the Scale-Up, Spread, and Sustainability of Health and Care Technologies | NASSS Framework | worldwide | systematic review | 28 previous technology implementation frameworks, of which 14 had taken a dynamic systems approach | to produce an evidence-based, theory-informed, and pragmatic framework to help predict and evaluate the success of a technology-supported health or social care program | Domain 1: The Condition Domain 2: The Technology Domain 3: The Value Proposition Domain 4: The Adopter System Domain 5: The Organization Domain 6: The Wider Context Domain 7: Interactions Between Domains and Adaptation Over Time |
| 12 | H Sarma, 2021 | Developing a conceptual framework for implementation science to evaluate a nutrition intervention scaled-up in a real-world setting | A comprehensive conceptual framework for implementation science | n/a | narrative review | n/a | The aim of this paper is to identify and develop a comprehensive conceptual framework using implementation science that can be applied to assess a nutrition intervention in a real-world setting. | The framework consisted of three domains:  Domain i – efficacy to effectiveness trials,  Domain ii – scaling-up and  Domain iii – sustainability.  These three domains encompass five components:  1. identifying an ‘effective’ intervention;  2. scaling-up and implementation fidelity;  3. course corrections during implementation;  4. promoting sustainability of interventions and consideration of a comprehensive methodological paradigm to identify ‘effective’ interventions and to  5. assess the process and outcome indicators of implementation. |
| 13 | J Iwelunmor, 2015 | Toward the sustainability of health interventions implemented in sub-Saharan Africa: a systematic review and conceptual framework | Conceptual framework of sustainability of interventions implemented in SSA | sub-Saharan Africa | Systematic review | multiple models such as PEN-3 model | The purpose of this study was to conduct a systematic review of empirical literature to explore how health interventions implemented in SSA are sustained. | Intervention  Socio-culture and community context Organizational settings |
| 14 | JC Moullin, 2019 | Systematic review of the Exploration, Preparation, Implementation, Sustainment (EPIS) framework | Exploration, Preparation, Implementation, Sustainment (EPIS) framework | Sweden [19], South Africa [20], and Mexico [21]. EPIS has also been used in other settings including public health [22], schools [23], and community health centers | systematic review | n/a | To examine and describe the research application of a widely used implementation framework, the Exploration, Preparation, Implementation, Sustainment (EPIS) framework. | Exploration-Preparation-Implementation-Sustainment |
| 15 | Kathy A Scott, 2018 | The Power of the Frame : Systems Transformation Framework for Health Care Leaders | The systems transformation framework | n/a | Descriptive | n/a | to create environments that are readiness for change  to plan, design, implement, and evaluate change within complex adaptive systems | 8 major domains:  1. vision,  2. leadership,  3. organizational culture,  4. organizational behavior, 5. organizational structure, 6. performance measurements,  7. internal learning,  8. external learning. |
| 16 | L Cooley, 2006 | Scaling up—from vision to large-scale change: a management framework for practitioners | Scaling up—from vision to large-scale change | worldwide | Descriptive | n/a | • To provide an easily understood and straightforward way for donors and investors to assess the scalability of proposed interventions; • To provide guidelines for designing pilot projects and other innovations “with scale in mind”;  • To provide tools and approaches to help practitioners manage the scaling up process | STEP 1: Develop a Scaling Up Plan  Task 1: Create a Vision  Task 2: Assess Scalability  Task 3: Fill Information Gaps  Task 4: Prepare a Scaling Up Plan STEP 2: Establish the Preconditions for Scaling Up  Task 5: Legitimize Change  Task 6: Build a Constituency  Task 7: Realign and Mobilize Resources  STEP 3: Implement the Scaling up Process  Task 8: Modify and Strengthen Organizations  Task 9: Coordinate Action  Task 10: Adapt Strategy and Maintain Momentum |
| 17 | LJ Damschroder, 2009 | Fostering implementation of health services research findings into practice: a consolidated framework for advancing implementation science（CFIR） | a consolidated framework for advancing implementation science（CFIR） | worldwide | a snowball sampling approach | n/a | To offer an overarching typology to promote implementation theory development and verification about what works where and why across multiple contexts. | intervention characteristics, outer setting, inner setting, characteristics of the individuals involved, and the process of implementation |
| 18 | Lisa M. Pfadenhauer, 2017 | Making sense of complexity in context and implementation: the Context and Implementation of Complex Interventions (CICI) framework | the Context and Implementation of Complex Interventions (CICI) framework | n/a | The Context and Implementation of Complex Interventions (CICI) framework was developed in an iterative manner and underwent extensive application | n/a | To develop a framework to facilitate the structured and comprehensive conceptualisation and assessment of context and implementation of complex interventions. | 1. context  2. implementation  3. setting |
| 19 | MA Scheirer, 2011 | An Agenda for Research on the Sustainability of Public Health Programs | Conceptual framework for sustainability of public health programs. | worldwide | Descriptive | n/a | To develop a theoretical model to guide health-related researchers and funders in making clear and transparent decisions about research methods to promote the sustainability of interventions. | Inputs Factors Affecting Sustainability  Obtaining financial resources Outcomes (programs sustained) |
| 20 | MJ De Silva, 2014 | Theory of Change: a theory-driven approach to enhance the Medical Research Council's framework for complex interventions | Theory of Change (ToC) | worldwide | a theory-driven approach to the design and evaluation of complex interventions by adapting and integrating a programmatic design and evaluation tool, | n/a | to develop complex medical interventions that are more likely to be effective, sustainable and scalable. | Development of complex interventions using Theory of Change Feasibility and piloting complex interventions using Theory of Change Evaluating complex interventions using Theory of Change Implementing complex interventions using Theory of Change |
| 21 | PM Barker, 2016 | A framework for scaling up health interventions: lessons from large-scale improvement initiatives in Africa | IHI Framework for Going to Full Scale. | LMICs | literature review | multiple models | To describe a framework for taking health interventions to full scale | 4 phases (1) Set-up(2) Develop the Scalable Unit(3) Test of Scale-up (4) Go to Full Scale  3 core components  (1) a sequence of activities  (2) underlying influencing factors  (3) support system |
| 22 | P Mendel, 2008 | Interventions in Organizational and Community Context: A Framework for Building Evidence on Dissemination and Implementation in Health Services Research | Framework of Dissemination in Health Services Intervention Research | n/a | Descriptive | n/a | to address this research-to-practice gap | Context of diffusion  Stages of Diffusion  Intervention Outcomes |
| 23 | RA Greene, 2014 | A Person-Focused Model of Care for the Twenty-First Century: A System-of-Systems Perspective | A Person-Focused Model of Care | America | Descriptive | prior models of chronic care with new empiric findings and complex adaptive system (CAS) theory | to offer a unifying focus for all participants in the health care delivery process | (a) the patient's health (spiritual-social-physical) in relation to the patient, the payer, the caregiver and the clinician;  (b) the person in relation to other entities (i.e. the patient);  (c) the caregiver in relation to other entities;  (d) the clinician in relation to other entities  (d) clinicians who have relationships with other entities; and  (e) payers who have relationships with other entities. |
| 24 | RC Shelton, 2018 | The Sustainability of Evidence-Based Interventions and Practices in Public Health and Health Care | Integrated sustainability framework. | worldwide | literature review | n/a | to address the gap between research and practice in public health | Influencing factors: Inner contextual factors; Processes; Characteristics of the intervention; Program sustainability; Outer contextual factors  Intervention process: development, adoption, implementation (with potential adaptations), and sustainability |
| 25 | RE Glasgow, 1999 | Evaluating the public health impact of health promotion interventions: the RE-AIM framework | RE-AIM | worldwide | Descriptive | n/a | To develop a comprehensive evaluation framework to facilitate progress in public health and community-based interventions | Five main components: reach, effectiveness, adoption, implementation, maintenance |
| 26 | B Gaglio, 2013 | The RE-AIM framework: a systematic review of use over time | RE-AIM | worldwide | systematic review | n/a | to (1) describe criteria for reporting on diverse dimensions of the RE-AIM framework, (2) review the empirical studies using RE-AIM framework, (3) emphasis experience learned from using RE-AIM, and (4) provide recommendations for future studies. | Five main components: reach, effectiveness, adoption, implementation, maintenance |
| 27 | SW Stirman, 2019 | The FRAME: an expanded framework for reporting adaptations and modifications to evidence-based interventions | The FRAME | n/a | Descriptive | n/a | 1.to improve feasibility or acceptability,  2.to increase reach or engagement, to improve fit (note that cultural modifications intended to improve fit are assigned a subcategory under this goal),  3.to reduce costs, to improve clinical outcomes,  4.to align the intervention with cultural values, norms, or priorities. | (1) when and how in the implementation process the modification was made, (2) whether the modification was planned/proactive (i.e., an adaptation) or unplanned/reactive, (3) who determined that the modification should be made, (4) what is modified, (5) at what level of delivery the modification is made, (6) type or nature of context or content-level modifications, (7) the extent to which the modification is fidelity-consistent, and (8) the reasons for the modification, including (a) the intent or goal of the modification (e.g., cultural adaptations, to reduce costs, etc.) and (b) contextual factors that influenced the decision. |
| 28 | WHO， 2009 | Practical guidance for scaling up health service innovations. Geneva: World Health Organization | ExpandNet framework | n/a | Descriptive | n/a | To provide a way of thinking about scaling up;  To identify conditions that lead to success;  To articulate strategic choices that have to be made;  To highlight actions that enhance the potential for success and sustainability | The innovation, The user organization, The environment, The resource team or organization, Strategic choice areas  The type of scaling up to be pursued.  Methods of dissemination and advocacy. Organisation of the scaling-up process. The cost of scaling up and mobilising resources to support it.  Monitoring and evaluation. |
|  |  |  |  |  |  |  |  |  |
|  |  |  |  |  |  |  |  |  |
| 29 | I Artieta-Pinedo, 2017 | Framework for the establishment of a feasible, tailored and effective perinatal education programme | multiple models | Spain | literature review and qualitative research with focus groups | diverse models | to assess women needs, identify factors that influence the desired outcomes, and propose a framework for developing new perinatal education | - Individual characteristics Demographic Personality External characteristics - Normative beliefs Behavioural beliefs Efficacy beliefs - Constraints Skills Intention - behaviour |
| 30 | J Hockley, 2019 | A framework for cross-cultural development and implementation of complex interventions to improve palliative care in nursing homes: the PACE steps to success programme | A cross-cultural adaptation framework | seven European countries | part of the PACE cluster randomised control trial | PARiHS framework | To offer a framework for the cross-cultural development and support necessary to implement a complex palliative care intervention in nursing homes. | Phase 1: adaptation and preparation of resources  Phase 2: training and implementation of the programme using the train-the-trainer model  Phase 3: supporting the implementation |
| 31 | H McMullen, 2015 | Explaining high and low performers in complex intervention trials: a new model based on diffusion of innovations theory | diffusion of innovation model | UK | ethnographic observation, 21 semi-structured interviews and analysis of routine documents | Greenhalgh et al.’s model | to a retrospective process evaluation of a complex intervention to introduce rapid HIV testing in a general practice setting | THE INNOVATION 1. SYSTEM ANTECEDENTS FOR INNOVATIONS 2. Absorptive capacity for new knowledge 3. Receptive context for change 4. SYSTEM READINESS FOR INNOVATION 5. COMMUNICATION AND INFLUENCE DIFFUSION 6. The Outer context 7. LINKAGE 8. THE IMPLEMENTATION PROCESS 9. The adopter 10. Assimilation |
| 32 | JM Stratil, 2020 | WICID framework version 1.0: criteria and considerations to guide evidence-informed decision-making on non-pharmacological interventions targeting COVID-19 | WICID framework | worldwide | case study systematic reivew qualitative analysis conceptual analysis | WHO-INTEGRATE framework  ‘best fit’ framework | To develop a decision-making framework adapted to the challenges of decision-making on non-pharmacological interventions to contain the global SARS-CoV-2 pandemic. | 1. implications for the course of the pandemic and its impact on health 2. implications for quality of life social well-being and mental health 3. implications for physical health beyond COVID-19 4. proportionality and implications for individual autonomy and fundamental rights 5. acceptability of and willingness to implement the measures 6. equity, equality, and fair distribution of benefits and burdens 7. societal and environmental implications 8 considerations 8. economic implications & considerations 9. resource implications & considerations 10. feasibility implications & considerations 11. interaction with and implications for the health system quality of evidence |
| 33 | Beets,2013 | Translating Policies Into Practice: A Framework to Prevent Childhood Obesity in Afterschool Programs | Conceptual Framework to Prevent Childhood Obesity Through Policy-Level Initiatives in Afterschool Programs | the United States | Descriptive | Afterschool programs | to discuss obesity prevention policies in ASPs and outline a conceptual framework for future research related to the successful adoption and implementation of such policies within the ASP setting | 1. Outside organizational partners (university, community, school) 2. National, state, local organzaition 3. Site 4. Asp leader 5. Frontline staffers 6. Child 7. Physical activity/diet |
| 34 | Apostolopoulos, 2018 | Moving alcohol prevention research forward—Part I: introducing a complex systems paradigm | complex adaptive system | the United States | A complex systems paradigm, grounded in socio-ecological and complex systems theories and computational modeling and simulation | n/a | (1) outlines the limitations inherent in current approaches to alcohol prevention research and explore how complex systems approaches can cope with these flaws; (2) offers the reader a fundamental comprehension of computational simulation modelling methods and an overview of heuristic conceptual models | government and business forces  college environment  student microsocial factors  alcohol misuse consequences |
| 35 | Birken, 2017 | Organizational theory for dissemination and implementation research | Organizational theory | the United States | Descriptive | transaction cost economics theory, institutional theory, contingency theories, and resource dependency theory | To demonstrate the utility of organizational theories for implementation research | Adoption  Implementation  Sustainment |
| **Studies obtained from the citation searching after the confirmation review** | | | | | | | | |
| **N** | **Author (year)** | **Name of the study** | **Name of the framework** | **Country** | **Method** | **Reference models** | **Purpose of the framework** | **Key concepts within the TMFs** |
| 36 | A. Milat, K, 2020 | Development and application of a hybrid implementation research framework to understand success in reducing under-5 mortality in Rwandamakers and implementers | Hybrid Framework for Understanding Interventions to Reduce Under-5 Mortality | LMICs | Descriptive | existing IR frameworks | to better understand U5M reduction in LMICs from identification of leading causes of amenable U5M, to EBI choice, identification, and testing of strategies, work to achieve sustainability at scale, and key contextual factors | **Ministry**  Leadership  Data availability and use  Donor coordination  culture  org structure  mission and values  Decision making process  Capacity development  Interaction with other ministries  Community and other stakeholder engagement  **National context**  Leadership  Budget  Support from other org  Legislation and policies  Economic development  Community advocacy and civil society  Accountability  Data availability and use  stability |
| 37 | N. S. Singh, 2021 | A tale of ‘politics and stars aligning’: analysing the sustainability of scaled up digital tools for front-line health workers in India | Conceptual framework for evaluating the scale-up and sustainability of digital solutions for front-line health workers. | Inida | Descriptive | n/a | to answer ‘Why have some digital health programmes scaled, and others not, and what are the implications for sustainability in India?’. To understand the factors that underpin the scale-up and sustainability of different typologies of digital health solutions in India | Perceived value  Adaptability  Data storage and governance  Government champions  Stakeholders networks  Frontline health worker engagement  Investing in evidence  Operationalization  Evolving stakeholder roles and relationships  National-state dynamics  Interoperability |
