# Supplementary material for: Adaptability, Scalability and Sustainability (ASaS) of complex health interventions: a systematic review of theories, models and frameworks
Source: Implement Sci. 2024 Jul 17;19:52. doi: 10.1186/s13012-024-01375-7 (PMC11253497; doi:10.1186/s13012-024-01375-7)
Supplement: Supplementary file 6 — Supplementary Material 6. [file 13012_2024_1375_MOESM6_ESM.docx]

| **Additional file 6: Applicability and feasibility of the collected TMFs** | | |
| --- | --- | --- |
| 1. **A Practical, Robust Implementation and Sustainability Model (PRISM) for Integrating Research Findings into Practice** | **Y=1  N=0** | **Justification of Decision** (If “yes”, How? If “no”, Why?) |
| 1. ***Is there pragmatic adequacy?*** | | |
| Are there clear concrete, feasible suggestions for how the theory proposed can actually be used in LMICs?  Does the author of the theory make a clear explicit link to use in LMICs? | 1 | The PRISM model focuses on integrating research findings into practice, emphasizing the interaction of health care programs with recipients to influence adoption, implementation, and effectiveness. While the model is comprehensive, its feasibility in LMICs would depend on the specific health care infrastructure, cultural factors, and economic conditions of those regions. |
| Do ALL concepts in the theory possess relevance for LMICs?  i.e. Is it Relevant? | 1 | PRISM integrates concepts from various models like the Diffusion of Innovations, Chronic Care Model, and others. These concepts are broad and could be relevant to LMICs, but their applicability would depend on the specific context of each LMIC. |
| Are all concepts of usefulness to LMICs included in the theory?  i.e. Is it Complete? | 1 | PRISM is developed from a range of existing models and elements with high face validity. However, whether it includes all concepts of usefulness for LMICs would depend on the unique challenges and needs of these regions. |
| 1. ***Is theory clearly articulated?*** | | |
| Are all the included concepts understandable/defined? | 1 | PRISM and its underlying models are well-documented and defined. However, the clarity for LMIC contexts would depend on local expertise and the ability to interpret these models in a culturally relevant manner. |
| Are the linkages between concepts clearly articulated or displayed? | 1 | The PRISM model and its underlying frameworks like RE-AIM articulate clear linkages between different concepts, focusing on aspects like reach, effectiveness, adoption, implementation, and maintenance. |
| Does the theory clearly present propositions that could be tested/used in an LMIC context? | 1 | PRISM provides a framework that can be adapted to different settings, including LMICs, but the clarity of propositions for these contexts would require specific tailoring and consideration of local conditions. |
| 1. ***Is there evidence of operational and empirical adequacy?*** | | |
| Have any studies used the theory in an LMIC context? | 1 | It is true that the PRISM framework has now been applied in some low and middle income countries (LMICs). Among the sources I looked for, a systematic review paper titled "A Systematic Review of Citation Analysis and Scoping for the Operationalization of the Practical, Robust Implementation, and Sustainability Model (PRISM)" mentions that out of 23 studies, 3 were conducted in LMICs. These studies covered different thematic areas, including health equity and multiple health issues, such as infectious diseases and reproductive health. |
| Have any studies that have used the theory supported their findings with data that relates to the theory or propositions? | 1 | Example: Using the Practical, Robust Implementation and Sustainability Model (PRISM) to qualitatively assess multilevel contextual factors to help plan, implement, evaluate, and disseminate health services programs |
| Does the empirical evidence from LMICs support the theory or propositions? | 1 | The empirical evidence from low-middle income countries (LMICs) supporting the Practical, Robust Implementation and Sustainability Model (PRISM) appears limited. A systematic review indicated that of the studies analyzed, only three were conducted in LMICs[105]. These studies primarily focused on health equity, addressing disparities in health affecting excluded or marginalized groups. |

| 1. **Adapting Evidence-Informed Complex Population Health Interventions for New Contexts** | **Y=1  N=0** | **Justification of Decision** (If “yes”, How? If “no”, Why?) |
| --- | --- | --- |
| 1. ***Is there pragmatic adequacy?*** | | |
| Are there clear concrete, feasible suggestions for how the theory proposed can actually be used in LMICs?  Does the author of the theory make a clear explicit link to use in LMICs? | Not clear | The review highlights the importance of adapting interventions to new contexts while saving resources. However, the complexities of different systems, norms, resources, and structures in LMICs make implementation challenging. Also, this document did not mention this issue. |
| Do ALL concepts in the theory possess relevance for LMICs?  i.e. Is it Relevant? | 1 | While the review doesn't explicitly focus on LMICs, it emphasizes adapting interventions to suit specific characteristics and needs of new contexts, which is relevant for LMICs. |
| Are all concepts of usefulness to LMICs included in the theory?  i.e. Is it Complete? | 1 | The review suggests adapting interventions to local contexts by considering the specific needs and characteristics of the target population, which should include all relevant concepts for LMICs. |
| 1. ***Is theory clearly articulated?*** | | |
| Are all the included concepts understandable/defined? | 1 | The review provides clear definitions and explanations of key concepts like adaptation, fidelity, and core components, enhancing their understandability. |
| Are the linkages between concepts clearly articulated or displayed? | 0 | The review synthesizes existing guidance on key concepts, the rationale for adaptation, types of adaptations, processes for conducting adaptation, and methodological approaches, but the clarity of linkages between these concepts is not explicitly detailed. |
| Does the theory clearly present propositions that could be tested/used in an LMIC context? | 0 | The review does not directly present testable propositions for LMICs, but it discusses the process of adaptation and re-evaluation of interventions in new contexts, which could be applicable to LMICs. |
| 1. ***Is there evidence of operational and empirical adequacy?*** | | |
| Have any studies used the theory in an LMIC context? | 0 | The review does not specifically address the application of its findings in LMICs, but the principles discussed are broadly applicable to adapting interventions in various contexts, including LMICs. |
| Have any studies that have used the theory supported their findings with data that relates to the theory or propositions? | Not clear | NA |
| Does the empirical evidence from LMICs support the theory or propositions? | Not clear | The review does not provide empirical evidence specifically from LMICs supporting the theory or its limitations. It synthesizes existing guidance and identifies limitations in the current understanding of adaptation processes. |

| **3.Factors influencing the implementation of chronic care models: A systematic literature review** | **Y=1  N=0** | **Justification of Decision** (If “yes”, How? If “no”, Why?) |
| --- | --- | --- |
| 1. ***Is there pragmatic adequacy?*** | | |
| Are there clear concrete, feasible suggestions for how the theory proposed can actually be used in LMICs?  Does the author of the theory make a clear explicit link to use in LMICs? | 1 | The chronic care model (CCM) requires careful consideration and planning for implementation. Its feasibility in LMICs would depend on factors at the healthcare provider, team, organization, and system levels. |
| Do ALL concepts in the theory possess relevance for LMICs?  i.e. Is it Relevant? | 1 | The review included studies on major chronic diseases prevalent in both developed and developing countries. The relevance of CCM concepts for LMICs would depend on the adaptability of these concepts to the specific health challenges and infrastructure in LMICs. |
| Are all concepts of usefulness to LMICs included in the theory?  i.e. Is it Complete? | 1 | The review covered a broad range of chronic diseases and healthcare settings, suggesting a comprehensive approach. However, whether all useful concepts for LMICs are included would depend on the specific healthcare needs and context of these countries. |
| 1. ***Is theory clearly articulated?*** | | |
| Are all the included concepts understandable/defined? | 1 | The review synthesized findings from various studies, implying that the concepts were defined and applied in diverse contexts. |
| Are the linkages between concepts clearly articulated or displayed? | 0 | The review identified key themes such as acceptability of the intervention, preparation of healthcare providers, support for patients, and resource needs for implementation. These themes suggest an interconnected approach to implementing CCMs, though explicit linkages between these concepts are not detailed in the review. |
| Does the theory clearly present propositions that could be tested/used in an LMIC context? | 0 | The review focused on identifying facilitators and barriers to implementing CCMs. While it provides insights into factors influencing successful implementation, it does not present specific testable propositions for LMIC contexts. |
| 1. ***Is there evidence of operational and empirical adequacy?*** | | |
| Have any studies used the theory in an LMIC context? | 1 | The review includes studies from various geographic regions, including a study from Africa, but primarily focuses on studies from the Americas and Europe. The applicability of the findings to LMIC contexts would depend on the similarity of healthcare systems and challenges. |
| Have any studies that have used the theory supported their findings with data that relates to the theory or propositions? | 1 | The review synthesizes findings from studies conducted in various settings |
| Does the empirical evidence from LMICs support the theory or propositions? | 1 | Example: Models of care for chronic conditions in low/middle-income countries: a 'best fit' framework synthesis |

| **4.The Dynamic Sustainability Framework: Addressing the Paradox of Sustainment Amid Ongoing Change** | **Y=1  N=0** | **Justification of Decision** (If “yes”, How? If “no”, Why?) |
| --- | --- | --- |
| 1. ***Is there pragmatic adequacy?*** | | |
| Are there clear concrete, feasible suggestions for how the theory proposed can actually be used in LMICs?  Does the author of the theory make a clear explicit link to use in LMICs? | 1 | The Dynamic Sustainability Framework (DSF) emphasizes ongoing adaptation and learning, making it potentially feasible for LMICs. However, its feasibility would depend on the specific context of LMICs, including healthcare infrastructure, cultural aspects, and resource availability. |
| Do ALL concepts in the theory possess relevance for LMICs?  i.e. Is it Relevant? | 1 | The DSF's focus on continuous adaptation and improvement of health interventions can be relevant for LMICs, especially given the varying healthcare contexts and challenges in these countries. |
| Are all concepts of usefulness to LMICs included in the theory?  i.e. Is it Complete? | 1 | The DSF's principles, including ongoing learning, adaptation, and fit with the local context, are broad enough to encompass concepts useful for LMICs. It stresses the importance of customizing interventions to local needs and conditions, which is crucial for LMIC settings. |
| 1. ***Is theory clearly articulated?*** | | |
| Are all the included concepts understandable/defined? | 1 | The DSF and its underlying concepts are well-articulated. However, understandability of these concepts in LMICs would depend on local capacity to interpret and apply them in culturally relevant ways. |
| Are the linkages between concepts clearly articulated or displayed? | 1 | The DSF presents clear linkages between its core concepts, including the interaction between interventions, practice settings, and the broader system, emphasizing the need for ongoing adaptation and fit. |
| Does the theory clearly present propositions that could be tested/used in an LMIC context? | 1 | The DSF proposes a framework for ongoing improvement and adaptation of health interventions, which can be tested in LMICs. It encourages the development of hypotheses that can be tested in diverse healthcare settings, including LMICs. |
| 1. ***Is there evidence of operational and empirical adequacy?*** | | |
| Have any studies used the theory in an LMIC context? | 1 | The DSF's principles are applicable to a wide range of settings, including LMICs. |
| Have any studies that have used the theory supported their findings with data that relates to the theory or propositions? | 1 | The Dynamic Sustainability Framework (DSF) has been applied in various studies, supporting findings with data that align with the theory or propositions of the framework.  Example: Sustaining a nursing best practice guideline in an acute care setting over 10 years: A mixed methods case study |
| Does the empirical evidence from LMICs support the theory or propositions? DO authors of empirical studies identify any limitations of the theory? If so, what are they? | 1 | Example: Sustainable support solutions for community-based rehabilitation workers in refugee camps: piloting telehealth acceptability and implementation |

| **5.A framework for implementing sustainable oral health promotion interventions** | **Y=1  N=0** | **Justification of Decision** (If “yes”, How? If “no”, Why?) |
| --- | --- | --- |
| 1. ***Is there pragmatic adequacy?*** | | |
| Are there clear concrete, feasible suggestions for how the theory proposed can actually be used in LMICs?  Does the author of the theory make a clear explicit link to use in LMICs? | 1 | Emphasizes implementation of sustainable oral health interventions through a multistage process. While it seems adaptable, feasibility in LMICs would depend on factors like resource availability, cultural relevance, and healthcare infrastructure in these contexts. |
| Do ALL concepts in the theory possess relevance for LMICs?  i.e. Is it Relevant? | 1 | Addresses diverse factors in oral health promotion, including prevention, intervention, and recovery, which are relevant for LMICs. However, the applicability of specific concepts would depend on the unique healthcare challenges in LMICs. |
| Are all concepts of usefulness to LMICs included in the theory?  i.e. Is it Complete? | 1 | Incorporates comprehensive approach, covering various stages from training and adoption to implementation and practice improvement. It should encompass concepts useful for LMICs, given a focus on adapting the interventions to the local context. |
| 1. ***Is theory clearly articulated?*** | | |
| Are all the included concepts understandable/defined? | 1 | The concepts are well-defined. However, their understandability in LMICs would depend on local expertise and the ability to interpret these concepts in a culturally relevant manner. |
| Are the linkages between concepts clearly articulated or displayed? | 1 | Presents clear linkages between core concepts, including stages of implementation and key factors influencing sustainability. It highlights the need for preparation, maintenance, and organizational readiness, which are integral to successful implementation. |
| Does the theory clearly present propositions that could be tested/used in an LMIC context? | 0 | Provides a structured approach for implementing interventions, but doesn't present specific testable propositions tailored for LMICs. However, principles can be adapted to create hypotheses suitable for testing in these contexts. |
| 1. ***Is there evidence of operational and empirical adequacy?*** | | |
| Have any studies used the theory in an LMIC context? | Not clear | Doesn't explicitly focus on LMICs. Provides generalizable model for implementing oral health interventions. Its application in LMICs would require adaptation to local healthcare systems and needs. |
| Have any studies that have used the theory supported their findings with data that relates to the theory or propositions? | 1 | Example: A qualitative assessment of factors influencing implementation and sustainability of evidence-based tobacco use treatment in Vietnam health centers |
| Does the empirical evidence from LMICs support the theory or propositions? DO authors of empirical studies identify any limitations of the theory? If so, what are they? | 0 | No. However, it discusses the potential for research networks, like the dental PBRNs, to advance the implementation of oral health innovations, which could be relevant for LMICs. |

| **6.A model for scale up of family health innovations in low-income and middle-income settings: a mixed methods study** | **Y=1  N=0** | **Justification of Decision** (If “yes”, How? If “no”, Why?) |
| --- | --- | --- |
| 1. ***Is there pragmatic adequacy?*** | | |
| Are there clear concrete, feasible suggestions for how the theory proposed can actually be used in LMICs?  Does the author of the theory make a clear explicit link to use in LMICs? | 1 | Many family health innovations that are efficacious and cost-effective fail to scale up for widespread use in LMICs. The AIDED model is specifically designed to address this challenge by providing a practical approach for scaling up health innovations in LMICs. |
| Do ALL concepts in the theory possess relevance for LMICs?  i.e. Is it Relevant? | 1 | The AIDED model's components are relevant for LMICs as they are designed to adapt innovations to local contexts and preferences, engage user groups effectively, and develop support systems within these environments. |
| Are all concepts of usefulness to LMICs included in the theory?  i.e. Is it Complete? | 1 | The model includes concepts that are essential for scaling up health innovations in LMICs, such as assessing the landscape, innovating to fit user receptivity, developing support, engaging with user groups, and devolving efforts for spreading the innovation. |
| 1. ***Is theory clearly articulated?*** | | |
| Are all the included concepts understandable/defined? | 1 | The concepts in the AIDED model are presented in a manner that aims to be practical and integrated, which should aid in their understandability, especially when applied to the context of LMICs. |
| Are the linkages between concepts clearly articulated or displayed? | 1 | The AIDED model outlines clear linkages between its components, emphasizing the importance of a comprehensive approach to scaling up innovations that includes understanding user needs, adapting to local contexts, and building and utilizing support systems. |
| Does the theory clearly present propositions that could be tested/used in an LMIC context? | 1 | The AIDED model itself is a proposition for effective scale-up in LMICs. Its practicality and adaptability to various health innovations make it a testable model in these contexts. |
| 1. ***Is there evidence of operational and empirical adequacy?*** | | |
| Have any studies used the theory in an LMIC context? | 1 | The AIDED model is specifically developed for use in LMICs, taking into account the unique challenges and environments of these settings. |
| Have any studies that have used the theory supported their findings with data that relates to the theory or propositions? | 1 | The model is derived from a mixed methods study that synthesizes experiences from various family health programs in LMICs, suggesting that it is grounded in practical evidence. |
| Does the empirical evidence from LMICs support the theory or propositions? | 1 | The study bases its model on empirical evidence gathered from experiences in LMICs. It acknowledges the unpredictable nature of complex adaptive systems and the importance of anticipating unintended consequences in these settings.   1. **Ghana Telemedicine Initiative**: This initiative focused on user-centered design, utilizing mobile phones by community health workers in remote areas. The program's design was based on end-user insights, leading to widespread adoption and expansion across Ghana​​. 2. **CommCare Platform in Bangladesh**: This platform enabled real-time data entry and monitoring, significantly benefiting frontline healthcare workers. It exemplifies the importance of capturing and utilizing real-time data to improve healthcare outcomes and justify further scaling​​. 3. **Community-Based Hypertension Improvement Project (ComHIP) in Ghana**: This project involved training community health workers to screen for hypertension, supported by a comprehensive digital health platform. It highlights the necessity of effective training and support systems for scaling digital health solutions​​. |

| **7.Normalisation Process Theory: a framework for developing, evaluating and implementing complex interventions** | **Y=1  N=0** | **Justification of Decision** (If “yes”, How? If “no”, Why?) |
| --- | --- | --- |
| 1. ***Is there pragmatic adequacy?*** | | |
| Are there clear concrete, feasible suggestions for how the theory proposed can actually be used in LMICs?  Does the author of the theory make a clear explicit link to use in LMICs? | 1 | The Normalisation Process Theory (NPT) is a tool that could aid in implementing complex interventions effectively, including in LMICs. Its focus on normalizing interventions into routine practice addresses a crucial gap in research implementation, which is relevant for LMICs. |
| Do ALL concepts in the theory possess relevance for LMICs?  i.e. Is it Relevant? | 1 | NPT's concepts are relevant for LMICs as they address the universal challenges of integrating complex health interventions into everyday practice, taking into account socio-organizational factors and the need for interventions to fit within existing systems and cultural contexts. |
| Are all concepts of usefulness to LMICs included in the theory?  i.e. Is it Complete? | 1 | NPT includes essential concepts for implementing interventions in any context, including LMICs. It considers coherence, cognitive participation, collective action, and reflexive monitoring, which are crucial for understanding and facilitating the adoption of interventions. |
| 1. ***Is theory clearly articulated?*** | | |
| Are all the included concepts understandable/defined? | 1 | NPT provides a clear framework for understanding and guiding the implementation of complex interventions. Its concepts are well-articulated and can be understood in the context of health systems, including those in LMICs. |
| Are the linkages between concepts clearly articulated or displayed? | 1 | NPT clearly links its components, highlighting the dynamic relationships between coherence, cognitive participation, collective action, and reflexive monitoring in the context of intervention implementation. |
| Does the theory clearly present propositions that could be tested/used in an LMIC context? | 1 | NPT provides a framework that can be used to assess and enhance the implementation potential of interventions, making it applicable for generating testable propositions in LMIC contexts. |
| 1. ***Is there evidence of operational and empirical adequacy?*** | | |
| Have any studies used the theory in an LMIC context? | 1 | While NPT is not exclusively focused on LMICs, its principles are broadly applicable to the implementation challenges in these contexts. However, no studies located in LMICs use this theory |
| Have any studies that have used the theory supported their findings with data that relates to the theory or propositions? | 1 | A qualitative systematic review of peer-reviewed NPT literature up to June 2012 found that NPT has been primarily used in qualitative research to study the implementation of complex interventions in healthcare settings. This includes studies in various fields such as e-health, telehealthcare, chronic health care, maternity care, and language interpretation services. These studies have originated mainly from the UK, Australia, Ireland, South Africa, and The Netherlands[106]. |
| Does the empirical evidence from LMICs support the theory or propositions? | 1 | Example: Effect of collaborative quality improvement on stillbirths, neonatal mortality and newborn care practices in hospitals of Telangana and Andhra Pradesh, India: evidence from a quasi-experimental mixed-methods study |

| **8.Advancing a Conceptual Model of Evidence-Based Practice Implementation in Public Service Sectors** | **Y=1  N=0** | **Justification of Decision** (If “yes”, How? If “no”, Why?) |
| --- | --- | --- |
| 1. ***Is there pragmatic adequacy?*** | | |
| Are there clear concrete, feasible suggestions for how the theory proposed can actually be used in LMICs?  Does the author of the theory make a clear explicit link to use in LMICs? | 1 | The conceptual model for implementing evidence-based practices (EBPs) in public service sectors could be feasible in LMICs if adapted to their specific context. The model is complex and involves multiple phases and contexts, so its applicability would depend on resources and local capacity in LMICs. |
| Do ALL concepts in the theory possess relevance for LMICs?  i.e. Is it Relevant? | 1 | The concepts within the model, including the phases of exploration, adoption/preparation, implementation, and sustainment, are broadly relevant for LMICs. However, their specific application would require consideration of local conditions, including cultural and systemic differences. |
| Are all concepts of usefulness to LMICs included in the theory?  i.e. Is it Complete? | 1 | The model includes a range of concepts that are likely useful for LMICs, particularly concerning the adaptability and sustainability of EBPs in varied contexts. These concepts include understanding local needs, resource constraints, and the sociopolitical environment. |
| 1. ***Is theory clearly articulated?*** | | |
| Are all the included concepts understandable/defined? | 1 | While the concepts in the model are comprehensive, their understandability in LMICs may vary based on local expertise and familiarity with EBP implementation frameworks. The concepts might require simplification or contextualization for better understanding in LMICs. |
| Are the linkages between concepts clearly articulated or displayed? | 1 | The model presents clear linkages between its various phases and the factors within each phase that influence EBP implementation. However, these linkages might need to be contextualized for LMICs to reflect their unique challenges and resources. |
| Does the theory clearly present propositions that could be tested/used in an LMIC context? | 1 | The model can potentially provide a basis for testable propositions in LMICs, especially regarding the adaptation and effectiveness of EBPs in these settings. Testing these propositions would require careful consideration of local variables and resources. |
| 1. ***Is there evidence of operational and empirical adequacy?*** | | |
| Have any studies used the theory in an LMIC context? | 1 | The conceptual model is not specifically designed for LMICs but offers a framework that can be adapted to different contexts, including LMICs. Its use in LMICs would require modifications to account for local realities, resources, and systemic differences. |
| Have any studies that have used the theory supported their findings with data that relates to the theory or propositions? | 1 | The model synthesizes a broad range of implementation science literature. While it does not specifically focus on LMICs, the principles it outlines could be supported by data from these contexts if properly adapted and tested. |
| Does the empirical evidence from LMICs support the theory or propositions? | 1 | it offers a comprehensive framework, its effectiveness and limitations in LMICs  South Africa: A study titled "Integrated knowledge translation to advance noncommunicable disease policy and practice in South Africa: application of the Exploration, Preparation, Implementation, and Sustainment (EPIS) framework" was conducted by researchers affiliated with Stellenbosch University, Cape Town, and other institutions. This study used the EPIS framework to develop, implement, and monitor an integrated knowledge translation approach to influence noncommunicable disease policy and practice in South Africa​​.  Brazil: The EPIS framework has also been applied in Brazil, as part of a range of projects that span high-, low-, and middle-income contexts. This was part of a broader set of applications of the EPIS framework across 11 countries, which also included the USA, Canada, Mexico, Sweden, Norway, Belgium, Australia, the UK, the Bahamas, and South Africa​​. |

| **9.Scaling Up Global Health Interventions: A Proposed Framework for Success** | **Y=1  N=0** | **Justification of Decision** (If “yes”, How? If “no”, Why?) |
| --- | --- | --- |
| 1. ***Is there pragmatic adequacy?*** | | |
| Are there clear concrete, feasible suggestions for how the theory proposed can actually be used in LMICs?  Does the author of the theory make a clear explicit link to use in LMICs? | 1 | The proposed framework for scaling up global health interventions is aimed at guiding the implementation of new programs, policies, or interventions in LMICs. It considers the unique challenges and requirements of these contexts, suggesting its feasibility in LMICs. |
| Do ALL concepts in the theory possess relevance for LMICs?  i.e. Is it Relevant? | 1 | Includes concepts specifically relevant to LMICs, such as the attributes of the tool or service being scaled up, attributes of implementers, delivery strategy, adopting community attributes, socio-political context, and the research context. |
| Are all concepts of usefulness to LMICs included in the theory?  i.e. Is it Complete? | 1 | Comprehensively covers concepts crucial for LMICs, including simplicity of interventions, robust technical policies, leadership, community engagement, political will, and the integration of research into implementation. |
| 1. ***Is theory clearly articulated?*** | | |
| Are all the included concepts understandable/defined? | 1 | Concepts clearly defined and explained, making them understandable for planners and implementers in LMICs. However, applicability would depend on local capacity to interpret and apply them in specific contexts. |
| Are the linkages between concepts clearly articulated or displayed? | 1 | Shows clear linkages between different components of the scaling-up process, emphasizing the interconnectedness of various factors such as the nature of the intervention, implementer attributes, and the socio-political environment. |
| Does the theory clearly present propositions that could be tested/used in an LMIC context? | 1 | Provides basis for developing testable propositions in LMICs, particularly in context of scaling up health interventions. Propositions can be tested through research and implementation projects. |
| 1. ***Is there evidence of operational and empirical adequacy?*** | | |
| Have any studies used the theory in an LMIC context? | 1 | Specifically tailored for use in LMICs, taking into account the unique challenges and environments of these settings for scaling up health interventions. |
| Have any studies that have used the theory supported their findings with data that relates to the theory or propositions? | 1 | Informed by literature and interviews with experts in large-scale change in global health, including experiences in LMICs. Suggests that it is grounded in practical evidence and experiences from these contexts. |
| Does the empirical evidence from LMICs support the theory or propositions? | 1 | Integrates insights from both literature and expert interviews, including experiences in LMICs. Emphasizes importance of adapting to local conditions and engaging with community and political stakeholders, which are key for successful implementation in LMICs.   1. **Simplicity of the Intervention**: Successful scaling up of antiretroviral therapy (ART) in Malawi and post-abortion care in Bolivia and Mexico was attributed to keeping the interventions simple, which is a key aspect of the framework​​. 2. **Engagement of Local Implementers and Communities**: In Bangladesh, the Bangladesh Rural Advancement Committee and in Pakistan, the "Lady Health Workers" program, are examples where engaging local implementers and communities played a crucial role in successful scale-up​​​​. 3. **Using State and Non-State Actors as Implementers**: NGOs have been instrumental in successful scale-up in many settings, as evidenced by their role in Bangladesh and in the work of non-governmental recipients of support from the Global Fund to Fight AIDS, Tuberculosis, and Malaria​​. 4. **Political Will and National Policies**: The successful scale-up of interventions like breastfeeding in Bolivia and Madagascar, post-abortion care in Guatemala, and DOTs in India demonstrates the importance of political will and national policies, which are key elements of the framework​​. 5. **Incorporating Research into Implementation**: In India, the scale-up of DOTs is an example of incorporating research into implementation, demonstrating the effectiveness of using evidence to guide the scale-up process and incorporate new learning​​. |

| **10.Implementation of sustainable complex interventions in health care services: the triple C model** | **Y=1  N=0** | **Justification of Decision** (If “yes”, How? If “no”, Why?) |
| --- | --- | --- |
| 1. ***Is there pragmatic adequacy?*** | | |
| Are there clear concrete, feasible suggestions for how the theory proposed can actually be used in LMICs?  Does the author of the theory make a clear explicit link to use in LMICs? | Not clear | The Triple C model, designed for implementing sustainable complex interventions in healthcare services, could be feasible in LMICs if adapted to local contexts. Its simplicity and focus on practicality make it potentially suitable for varied healthcare settings, including those with resource constraints typical in LMICs. |
| Do ALL concepts in the theory possess relevance for LMICs?  i.e. Is it Relevant? | 1 | The model's concepts of consultation, collaboration, and consolidation are broadly relevant for LMICs. These stages emphasize critical aspects like stakeholder engagement, teamwork, and sustainable practices, which are crucial in any healthcare context, including LMICs. |
| Are all concepts of usefulness to LMICs included in the theory?  i.e. Is it Complete? | 1 | The Triple C model includes concepts crucial for implementing complex interventions in LMICs, such as stakeholder engagement, clear communication, and ensuring sustainability. These concepts are vital for the successful implementation of healthcare interventions in resource-constrained settings. |
| 1. ***Is theory clearly articulated?*** | | |
| Are all the included concepts understandable/defined? | 1 | The model presents its concepts in a straightforward and practical manner, which should enhance their understandability in LMICs. Its focus on real-world applicability makes it a potentially user-friendly framework for healthcare workers in these regions. |
| Are the linkages between concepts clearly articulated or displayed? | 1 | The model demonstrates clear linkages between its three stages, highlighting the importance of a holistic and integrated approach to implementing and sustaining healthcare interventions. |
| Does the theory clearly present propositions that could be tested/used in an LMIC context? | 1 | The Triple C model can provide a basis for developing testable propositions in LMICs, especially in terms of its effectiveness in implementing and sustaining complex healthcare interventions. |
| 1. ***Is there evidence of operational and empirical adequacy?*** | | |
| Have any studies used the theory in an LMIC context? | 0 | While the model is not specifically tailored for LMICs, its principles are applicable to various healthcare settings. Its application in LMICs would require adjustments to accommodate specific local challenges and resource limitations. |
| Have any studies that have used the theory supported their findings with data that relates to the theory or propositions? | 0 | The model is grounded in a review of relevant literature and practical application in healthcare settings. However, the specific applicability to LMICs would need further exploration and data support from these contexts. |
| Does the empirical evidence from LMICs support the theory or propositions? | 0 | The model has been applied in various healthcare projects, demonstrating its practical utility. However, its limitations include resource availability, staff capacity, and the need for business intelligence tools. These factors would be crucial considerations in LMICs, where resource constraints are common. |

| **11.Beyond Adoption: A New Framework for Theorizing and Evaluating Nonadoption, Abandonment, and Challenges to the Scale-Up, Spread, and Sustainability of Health and Care Technologies** | **Y=1  N=0** | **Justification of Decision** (If “yes”, How? If “no”, Why?) |
| --- | --- | --- |
| 1. ***Is there pragmatic adequacy?*** | | |
| Are there clear concrete, feasible suggestions for how the theory proposed can actually be used in LMICs?  Does the author of the theory make a clear explicit link to use in LMICs? | 0 | Aims to predict and evaluate the success of technology-supported health or social care programs, which can be relevant for LMICs. However, its feasibility in LMICs would depend on the local context, available resources, and specific health care needs of these regions. |
| Do ALL concepts in the theory possess relevance for LMICs?  i.e. Is it Relevant? | 1 | Designed to be broadly applicable across health and social care technologies. Its potential uses, such as informing design of new technologies and planning their implementation, are relevant for LMICs. |
| Are all concepts of usefulness to LMICs included in the theory?  i.e. Is it Complete? | 1 | Built upon empirical case studies and a hermeneutic literature review, covering key domains such as the nature of health conditions, technology, adopter systems, and the wider context. These domains are likely to include concepts useful for LMICs. |
| ***If answer “yes” to question 1 then proceed to question 2. If answer “no” to question 1 then consider exclusion from review*** | | |
| **All papers that answer “yes” to question 1:** | **Y=1 N=0** | If “yes”, How? If “no”, Why? |
| 1. ***Is theory clearly articulated?*** | | |
| Are all the included concepts understandable/defined? | Yes |  |
| Are the linkages between concepts clearly articulated or displayed? | Yes | The NASSS framework links various domains influencing the adoption, spread, and sustainability of health technologies. |
| Does the theory clearly present propositions that could be tested/used in an LMIC context? | No | While not exclusively designed for LMICs, can provide basis for developing testable propositions in these settings, particularly in the adaptation of health technologies. |
| 1. ***Is there evidence of operational and empirical adequacy?*** | | |
| Have any studies used the theory in an LMIC context? | 1 | Development considers a broad range of contexts, including potentially LMICs. Its application in LMICs would require adjustments to fit the specific challenges and conditions of these regions.  Example: Assessing the potential of wearable health monitors for health system strengthening in low- and middle-income countries: a prospective study of technology adoption in Cambodia |
| Have any studies that have used the theory supported their findings with data that relates to the theory or propositions? | 1 | Challenges and opportunities in employing digital health to address self-management needs of people with NCDs in India |
| Does the empirical evidence from LMICs support the theory or propositions? | 1 | Example: Assessing the potential of wearable health monitors for health system strengthening in low- and middle-income countries: a prospective study of technology adoption in Cambodia |

| **12.Developing a conceptual framework for implementation science to evaluate a nutrition intervention scaled-up in a real-world setting** | **Y=1  N=0** | **Justification of Decision** (If “yes”, How? If “no”, Why?) |
| --- | --- | --- |
| 1. ***Is there pragmatic adequacy?*** | | |
| Are there clear concrete, feasible suggestions for how the theory proposed can actually be used in LMICs?  Does the author of the theory make a clear explicit link to use in LMICs? | 1 | Offers a methodological paradigm and practical components, such as identifying effective interventions and ensuring scalability, which seem feasible for application in LMICs. It has been successfully applied in Bangladesh, an LMIC, indicating its practical feasibility. |
| Do ALL concepts in the theory possess relevance for LMICs?  i.e. Is it Relevant? | 1 | All the concepts, such as efficacy, scaling-up, and sustainability, are relevant to LMICs, as these are critical factors in the successful implementation of nutrition interventions in these settings. |
| Are all concepts of usefulness to LMICs included in the theory?  i.e. Is it Complete? | 1 | The theory includes essential concepts like implementation fidelity and course corrections during implementation, which are crucial for LMICs to ensure interventions are adapted to local contexts and needs. |
| 1. ***Is theory clearly articulated?*** | | |
| Are all the included concepts understandable/defined? | 1 | Concepts are well-defined and detailed, suggesting that they are understandable. However, the specific context of LMICs might require additional clarification or adaptation. |
| Are the linkages between concepts clearly articulated or displayed? | 1 | Framework outlines clear linkages between its components, such as transition from identifying effective interventions to scaling-up and ensuring sustainability. |
| Does the theory clearly present propositions that could be tested/used in an LMIC context? | 1 | Presents clear propositions that can be tested and used in LMICs, as demonstrated by its application in the Bangladesh program. |
| 1. ***Is there evidence of operational and empirical adequacy?*** | | |
| Have any studies used the theory in an LMIC context? | 1 | Has been used in a real-world setting in Bangladesh, an LMIC, indicating its applicability in such contexts. |
| Have any studies that have used the theory supported their findings with data that relates to the theory or propositions? | 1 | Application in Bangladesh and its successful outcomes suggest that empirical data from LMICs can support the theory. However, more widespread application and data needed for robust validation. |
| Does the empirical evidence from LMICs support the theory or propositions? | 1 | The successful application in Bangladesh provides empirical support. Paper does not explicitly discuss limitations related to LMICs, but general limitations of implementation science frameworks could be relevant, such as the need for context-specific adaptations and the challenge of measuring long-term sustainability.   1. "Developing a conceptual framework for implementation science to evaluate a nutrition intervention scaled-up in a real-world setting" published in Public Health Nutrition​​. 2. "Operationalizing Implementation Science in Nutrition: The Implementation Science Initiative in Kenya and Uganda" published on PubMed​​. |

| **13.Toward the sustainability of health interventions implemented in sub-Saharan Africa: a systematic review and conceptual framework** | **Y=1  N=0** | **Justification of Decision** (If “yes”, How? If “no”, Why?) |
| --- | --- | --- |
| 1. ***Is there pragmatic adequacy?*** | | |
| Are there clear concrete, feasible suggestions for how the theory proposed can actually be used in LMICs?  Does the author of the theory make a clear explicit link to use in LMICs? | 1 | The study indicates a critical need to understand the sustainability of health interventions in SSA, a region with limited resources, healthcare worker shortages, and weak health systems. This suggests that while sustainability is crucial, achieving it in LMICs presents significant challenges. |
| Do ALL concepts in the theory possess relevance for LMICs?  i.e. Is it Relevant? | 1 | The study specifically addresses the sustainability of health interventions in SSA, indicating the relevance of this concept in LMICs. It focuses on both communicable diseases (CDs) and non-communicable diseases (NCDs), which are pertinent health issues in these regions. |
| Are all concepts of usefulness to LMICs included in the theory?  i.e. Is it Complete? | 1 | The study reviews literature on various health interventions in SSA, suggesting an inclusive approach. However, it's not clear if all useful concepts for LMICs are included, as the focus is primarily on sustainability. |
| ***If answer “yes” to question 1 then proceed to question 2. If answer “no” to question 1 then consider exclusion from review*** | | |
| **All papers that answer “yes” to question 1:** | **Y=1 N=0** | If “yes”, How? If “no”, Why? |
| 1. ***Is theory clearly articulated?*** | | |
| Are all the included concepts understandable/defined? | 1 | While the study discusses sustainability, it notes that only 21 out of 41 reviewed studies had clear definitions of sustainability, suggesting potential issues with understandability and consistency in concept definition. |
| Are the linkages between concepts clearly articulated or displayed? | 1 | The study highlights the importance of community ownership and mobilization as facilitators of sustainability, linking these factors to successful intervention outcomes. However, it does not provide detailed information on the linkages between different sustainability factors. |
| Does the theory clearly present propositions that could be tested/used in an LMIC context? | 1 | The study does not explicitly present testable propositions but reviews existing literature to understand factors influencing sustainability in SSA. |
| 1. ***Is there evidence of operational and empirical adequacy?*** | | |
| Have any studies used the theory in an LMIC context? | 1 | Social work in Africa: Decolonizing methodologies and approaches |
| Have any studies that have used the theory supported their findings with data that relates to the theory or propositions? | 1 | Example: Gaps in hypertension guidelines in low-and middle-income versus high-income countries: a systematic review |
| Does the empirical evidence from LMICs support the theory or propositions? | 1 | The study concludes that sustainability is a core component of health interventions in SSA, based on the empirical evidence reviewed. It acknowledges challenges such as limited resources and weak health systems, but does not explicitly discuss the limitations of the sustainability concept in LMICs. |

| **14.Systematic review of the Exploration, Preparation, Implementation, Sustainment (EPIS) framework** | **Y=1  N=0** | **Justification of Decision** (If “yes”, How? If “no”, Why?) |
| --- | --- | --- |
| 1. ***Is there pragmatic adequacy?*** | | |
| Are there clear concrete, feasible suggestions for how the theory proposed can actually be used in LMICs?  Does the author of the theory make a clear explicit link to use in LMICs? | 1 | The review does not specifically address LMICs, but the EPIS framework's focus on inner and outer context factors, innovation factors, and bridging factors suggests its potential adaptability to diverse settings, including LMICs. However, its feasibility in LMICs would depend on the specific context and resources available. |
| Do ALL concepts in the theory possess relevance for LMICs?  i.e. Is it Relevant? | 1 | The concepts within the EPIS framework, including the focus on different stages of implementation and context factors, are broadly relevant for application in various settings, potentially including LMICs. |
| Are all concepts of usefulness to LMICs included in the theory?  i.e. Is it Complete? | 1 | The EPIS framework covers a range of factors crucial for the implementation of EBPs, such as inner and outer context factors, which are likely to be relevant in LMICs. However, the review does not explicitly address whether all useful concepts for LMICs are included. |
| 1. ***Is theory clearly articulated?*** | | |
| Are all the included concepts understandable/defined? | 1 | The EPIS framework is applied in various research projects, indicating that its concepts are generally understandable. However, the level of understanding might vary depending on the specific context and background of the implementers. |
| Are the linkages between concepts clearly articulated or displayed? | 1 | The EPIS framework includes the examination of factors that link the outer system and inner organizational context, which suggests a focus on the clarity of linkages between different implementation factors. |
| Does the theory clearly present propositions that could be tested/used in an LMIC context? | 1 | While the review does not explicitly focus on LMICs, the EPIS framework’s applicability in public sector settings indicates that it could potentially present testable propositions relevant to LMICs. |
| 1. ***Is there evidence of operational and empirical adequacy?*** | | |
| Have any studies used the theory in an LMIC context? | 1 | Does not provide specific information about application in LMICs. Studies were conducted in various public sector settings, but the review does not detail geographical/economic contexts of settings. |
| Have any studies that have used the theory supported their findings with data that relates to the theory or propositions? | 1 | Inclusion of 67 articles in the review, representing 49 unique research projects, suggests empirical support in context of EBP implementation. However, depth of EPIS application was rated as moderate, and review does not specifically discuss data from LMICs. |
| Does the empirical evidence from LMICs support the theory or propositions? | 1 | Indicates widespread use with promising characteristics for implementation research. Suggests more precise operationalization and increased depth and breadth of application for future use. Does not explicitly discuss limitations in context of LMICs or empirical evidence from such settings. |

| **15. The Power of the Frame : Systems Transformation Framework for Health Care Leaders** | **Y=1  N=0** | **Justification of Decision** (If “yes”, How? If “no”, Why?) |
| --- | --- | --- |
| 1. ***Is there pragmatic adequacy?*** | | |
| Are there clear concrete, feasible suggestions for how the theory proposed can actually be used in LMICs?  Does the author of the theory make a clear explicit link to use in LMICs? | 1 | Adaptable and could be relevant for LMICs, especially in structuring health care leadership and change management, though does not specifically address LMIC contexts. |
| Do ALL concepts in the theory possess relevance for LMICs?  i.e. Is it Relevant? | 1 | While the concepts are generally applicable to healthcare systems, their direct relevance to LMICs might require contextual adaptations given different health care challenges and resource limitations in LMICs. |
| Are all concepts of usefulness to LMICs included in the theory?  i.e. Is it Complete? | 1 | Includes comprehensive concepts for organizational change, which are relevant for LMICs, but it does not specifically address LMIC-unique challenges. |
| 1. ***Is theory clearly articulated?*** | | |
| Are all the included concepts understandable/defined? | 1 | Defines and explains the concepts clearly, providing a detailed understanding of each domain. |
| Are the linkages between concepts clearly articulated or displayed? | 1 | Articulates the interconnections between the different domains, demonstrating how each aspect contributes to overall systems transformation. |
| Does the theory clearly present propositions that could be tested/used in an LMIC context? | 1 | Provides structured approach for health care systems transformation, which can be applied in LMICs, although it does not offer LMIC-specific propositions. |
| 1. ***Is there evidence of operational and empirical adequacy?*** | | |
| Have any studies used the theory in an LMIC context? | 0 | Does not specifically mention application in LMIC studies. |
| Have any studies that have used the theory supported their findings with data that relates to the theory or propositions? | 0 | No direct reference to empirical data from LMICs. |
| Does the empirical evidence from LMICs support the theory or propositions? | 0 | Not directly supported by empirical evidence from LMICs, and it does not discuss limitations of the theory based on such evidence. |

| **16.Scaling Up—From Vision to Large-Scale Change: A Management Framework for Practitioners** | **Y=1  N=0** | **Justification of Decision** (If “yes”, How? If “no”, Why?) |
| --- | --- | --- |
| 1. ***Is there pragmatic adequacy?*** | | |
| Are there clear concrete, feasible suggestions for how the theory proposed can actually be used in LMICs?  Does the author of the theory make a clear explicit link to use in LMICs? | 0 | Practical and field-tested nature suggests it could be feasible in LMICs, especially since it has been applied in diverse sectors relevant to these contexts. |
| Do ALL concepts in the theory possess relevance for LMICs?  i.e. Is it Relevant? | 1 | Concepts, such as strategic planning and change management, are highly relevant for LMICs, considering the need for effective scaling up of interventions in these regions. |
| Are all concepts of usefulness to LMICs included in the theory?  i.e. Is it Complete? | 1 | Appears to include comprehensive range of concepts important for scaling up in LMICs, including planning, resource allocation, and maintaining momentum. |
| 1. ***Is theory clearly articulated?*** | | |
| Are all the included concepts understandable/defined? | 1 | Presented as set of manageable tasks, which implies clarity and understandability in its application. |
| Are the linkages between concepts clearly articulated or displayed? | 1 | Step-by-step approach and the division into specific tasks suggest clear linkages between different aspects of scaling up. |
| Does the theory clearly present propositions that could be tested/used in an LMIC context? | 0 | Does not explicitly present propositions for LMICs, yet its broad application across various sectors implies its relevance and potential applicability in these contexts. |
| 1. ***Is there evidence of operational and empirical adequacy?*** | | |
| Have any studies used the theory in an LMIC context? | 1 | Example: Best practices in scaling digital health in low and middle income countries |
| Have any studies that have used the theory supported their findings with data that relates to the theory or propositions? | 0 |  |
| Does the empirical evidence from LMICs support the theory or propositions? | 1 | 1. **Program Characteristics**: Focusing on user-centered design and tangible benefits addressing unmet needs​​. (Best practices in scaling digital health in low and middle income countries) 2. **Human Factors**: Emphasizing training and preparation of end-users for effective utilization of digital health solutions​​. 3. **Technical Factors**: Prioritizing simplicity, interoperability, and adaptability in digital health initiatives for easier scalability and sustainability​​. 4. **Healthcare Ecosystem**: Aligning with broader healthcare policies and ensuring regulatory compliance and sustainable funding​​. 5. **Extrinsic Ecosystem**: Considering the reliability of networks, availability of electricity, and infrastructure support for digital health projects​​.   Examples include the Ghana-based telemedicine initiative, the Community-Based Hypertension Improvement Project (ComHIP) in Ghana, and the M-TIBA mobile health wallet in Kenya, each demonstrating different aspects of effective scaling in digital health​​. These case studies underline the importance of close engagement with stakeholders, dynamic scaling processes, and the crucial role of the private sector in facilitating the scaling of digital health initiatives in LMICs​​. |

| **17.Fostering implementation of health services research findings into practice: a consolidated framework for advancing implementation science** | **Y=1  N=0** | **Justification of Decision** (If “yes”, How? If “no”, Why?) |
| --- | --- | --- |
| 1. ***Is there pragmatic adequacy?*** | | |
| Are there clear concrete, feasible suggestions for how the theory proposed can actually be used in LMICs?  Does the author of the theory make a clear explicit link to use in LMICs? | 1 | The CFIR's comprehensive and pragmatic approach is likely to be applicable in LMICs, considering its flexibility to adapt to various contexts and its focus on both internal and external environmental factors relevant to implementation. |
| Do ALL concepts in the theory possess relevance for LMICs?  i.e. Is it Relevant? | 1 | The CFIR includes a wide range of concepts that are relevant to LMICs, given the diversity and complexity of health service implementation challenges in these contexts. |
| Are all concepts of usefulness to LMICs included in the theory?  i.e. Is it Complete? | 1 | The CFIR appears to include a comprehensive set of concepts crucial for understanding and guiding the implementation process in various contexts, including LMICs. |
| ***If answer “yes” to question 1 then proceed to question 2. If answer “no” to question 1 then consider exclusion from review*** | | |
| **All papers that answer “yes” to question 1:** | **Y=1 N=0** | If “yes”, How? If “no”, Why? |
| 1. ***Is theory clearly articulated?*** | | |
| Are all the included concepts understandable/defined? | 1 | The CFIR provides explicit definitions for each construct, suggesting that the concepts are clearly defined and should be understandable to practitioners and researchers. |
| Are the linkages between concepts clearly articulated or displayed? | 1 | The CFIR's structure, which organizes constructs into distinct domains while acknowledging their interactions, indicates an effort to clarify the linkages between different aspects of implementation. |
| Does the theory clearly present propositions that could be tested/used in an LMIC context? | 1 | While the CFIR is not specifically designed for LMICs, its comprehensive nature and adaptability make it potentially suitable for developing testable propositions in these contexts. |
| 1. ***Is there evidence of operational and empirical adequacy?*** | | |
| Have any studies used the theory in an LMIC context? | 1 | Does not explicitly detail the CFIR's application in LMICs, but its broad and inclusive nature suggests it could be applied in such studies. |
| Have any studies that have used the theory supported their findings with data that relates to the theory or propositions? | 1 | The CFIR is built upon a synthesis of existing theories, indicating a strong foundation in empirical data. However, its application and validation in LMIC-specific contexts may require further research. |
| Does the empirical evidence from LMICs support the theory or propositions? DO authors of empirical studies identify any limitations of the theory? If so, what are they? | 1 | The CFIR is designed to adapt to various settings, including LMICs, and is based on empirical evidence from existing theories. However, its practical limitations in specific LMIC contexts, such as resource constraints or cultural differences, are not explicitly discussed. Framework's effectiveness in these contexts would likely depend on adaptation to local needs and circumstances. |

| **18.Making sense of complexity in context and implementation: the Context and Implementation of Complex Interventions (CICI) framework** | **Y=1  N=0** | **Justification of Decision** (If “yes”, How? If “no”, Why?) |
| --- | --- | --- |
| 1. ***Is there pragmatic adequacy?*** | | |
| Are there clear concrete, feasible suggestions for how the theory proposed can actually be used in LMICs?  Does the author of the theory make a clear explicit link to use in LMICs? | Not clear | Comprehensive nature suggests it could be feasibly applied in LMICs, especially given its emphasis on context, which is crucial in varying resource settings. |
| Do ALL concepts in the theory possess relevance for LMICs?  i.e. Is it Relevant? | 1 | Concepts, such as socio-economic and cultural context, are highly relevant to LMICs, as these factors significantly influence success of interventions in these settings. |
| Are all concepts of usefulness to LMICs included in the theory?  i.e. Is it Complete? | 1 | Seems to include wide range of concepts important for understanding and implementing complex interventions in various contexts, likely encompassing those relevant to LMICs. |
| 1. ***Is theory clearly articulated?*** | | |
| Are all the included concepts understandable/defined? | 1 | Appears to be structured in a way that makes its concepts clear and understandable, with tools provided for operationalization, such as checklists and data extraction tools. |
| Are the linkages between concepts clearly articulated or displayed? | 1 | Emphasizes interaction between its different dimensions, suggesting that it provides clarity on the linkages between context, implementation, setting, and the intervention itself. |
| Does the theory clearly present propositions that could be tested/used in an LMIC context? | Not clear | Not specifically tailored for LMICs. Focus on diverse contexts and settings makes it potentially suitable for formulating propositions relevant to these areas. |
| 1. ***Is there evidence of operational and empirical adequacy?*** | | |
| Have any studies used the theory in an LMIC context? | 1 | Example: A model for national assessment of barriers for implementing digital technology interventions to improve hypertension management in the public health care system in India |
| Have any studies that have used the theory supported their findings with data that relates to the theory or propositions? | Not clear | NA |
| Does the empirical evidence from LMICs support the theory or propositions? | 1 | Example: Exemplars in vaccine delivery protocol: a case-study-based identification and evaluation of critical factors in achieving high and sustained childhood immunisation coverage in selected low-income and lower-middle-income countries |

| **19.An Agenda for Research on the Sustainability of Public Health Programs** | **Y=1  N=0** | **Justification of Decision** (If “yes”, How? If “no”, Why?) |
| --- | --- | --- |
| 1. ***Is there pragmatic adequacy?*** | | |
| Are there clear concrete, feasible suggestions for how the theory proposed can actually be used in LMICs?  Does the author of the theory make a clear explicit link to use in LMICs? | Not clear | Suggestions provided could be adapted for use in LMICs, though specific challenges related to resource constraints and different health system dynamics in these settings would need to be considered. |
| Do ALL concepts in the theory possess relevance for LMICs?  i.e. Is it Relevant? | 1 | The concepts of sustainability, including the continuation of program benefits and activities, are relevant to LMICs as these regions often face issues with sustaining health interventions post-funding. |
| Are all concepts of usefulness to LMICs included in the theory?  i.e. Is it Complete? | 1 | Covers comprehensive range of concepts relevant to sustainability, which would likely be useful in the context of LMICs. |
| 1. ***Is theory clearly articulated?*** | | |
| Are all the included concepts understandable/defined? | 1 | Provides clear definitions and explanations of the concepts, making them understandable for application in different contexts, including LMICs. |
| Are the linkages between concepts clearly articulated or displayed? | 1 | Links sustainability to earlier stages of intervention development, adoption, and implementation, providing a clear understanding of how sustainability is interconnected with these processes. |
| Does the theory clearly present propositions that could be tested/used in an LMIC context? | Not clear | Doesn't specifically focus on LMICs, but principles and frameworks it discusses could be used to develop propositions relevant to sustainability in these contexts. |
| 1. ***Is there evidence of operational and empirical adequacy?*** | | |
| Have any studies used the theory in an LMIC context? | Not clear | NA |
| Have any studies that have used the theory supported their findings with data that relates to the theory or propositions? | 1 | The sustainability of new programs and innovations: a review of the empirical literature and recommendations for future research  Also, many other studies used this theory   1. **Sustainable by Design: A Systematic Review of Factors for Health Promotion Program Sustainability**: This study conducted a systematic review to identify barriers and facilitators influencing the sustainability of health promotion programs (HPP). It highlighted the lack of clear sustainability definitions, infrequent use of sustainability frameworks, and limited understanding of factors influencing sustainability. The study identified 83 barriers and 191 facilitators for HPP sustainability, categorized into 14 factors. The study provides clarity on sustainability definitions and frameworks, suggesting the need for practitioners to clearly articulate elements for sustainability, select appropriate frameworks, and consider these factors in program planning​​. 2. **A Conceptual Model for Building Program Sustainability in Public Health Settings**: This research is part of a larger randomized control trial evaluating the effectiveness of the Program Sustainability Action Planning Model and Training Curricula. It aims to establish a conceptual model for program sustainability and related capacity-building interventions. This study contributes to implementation science by documenting the development and implementation of a novel training intervention for program sustainability in public health. It proposes an empirically-grounded conceptual model for implementing sustainability capacity-building interventions, focusing on factors such as organizational capacity, funding stability, strategic planning, and partner engagement​​. |
| Does the empirical evidence from LMICs support the theory or propositions? | 0 | Provides theoretical and practical foundation for sustainability research, but does not explicitly discuss the limitations of applying these concepts in the context of LMICs or provide empirical evidence from such settings. The effectiveness of these concepts in LMICs would likely depend on context-specific adaptations and the availability of resources. |

| **20.Theory of Change: a theory-driven approach to enhance the Medical Research Council's framework for complex interventions** | **Y=1  N=0** | **Justification of Decision** (If “yes”, How? If “no”, Why?) |
| --- | --- | --- |
| 1. ***Is there pragmatic adequacy?*** | | |
| Are there clear concrete, feasible suggestions for how the theory proposed can actually be used in LMICs?  Does the author of the theory make a clear explicit link to use in LMICs? | 1 | The ToC approach has been piloted for mental health projects in LMICs, indicating its feasibility in these contexts. The adaptability of ToC to local conditions and resources in LMICs is a key feature. |
| Do ALL concepts in the theory possess relevance for LMICs?  i.e. Is it Relevant? | 1 | The concepts within the ToC framework, including stakeholder engagement and the identification of causal pathways, are highly relevant for LMICs, especially for mental health interventions. |
| Are all concepts of usefulness to LMICs included in the theory?  i.e. Is it Complete? | 1 | The ToC framework appears to include a comprehensive range of concepts crucial for designing, implementing, and evaluating complex interventions in LMICs. |
| 1. ***Is theory clearly articulated?*** | | |
| Are all the included concepts understandable/defined? | 1 | ToC provides a structured approach to intervention design, with clear definitions and visual representations, making it understandable for implementers and stakeholders. |
| Are the linkages between concepts clearly articulated or displayed? | 1 | The ToC approach emphasizes the identification and articulation of causal pathways, preconditions, and assumptions, thereby clarifying the linkages between various components of an intervention. |
| Does the theory clearly present propositions that could be tested/used in an LMIC context? | 1 | ToC enables the formulation of specific, contextually relevant propositions and hypotheses for interventions in LMICs. |
| 1. ***Is there evidence of operational and empirical adequacy?*** | | |
| Have any studies used the theory in an LMIC context? | 1 | Describes application of ToC in mental health interventions in several LMICs, demonstrating its practical use in these settings. |
| Have any studies that have used the theory supported their findings with data that relates to the theory or propositions? | 1 | The application of ToC in various projects suggests empirical support for its effectiveness in designing and evaluating complex interventions, although specific empirical data from LMICs is not extensively detailed. |
| Does the empirical evidence from LMICs support the theory or propositions? | 1 | Presents ToC as a useful and adaptable framework for complex interventions in LMICs. However, challenges such as the need for extensive stakeholder involvement and the difficulty in operationalizing true ownership of ToC maps by stakeholders are acknowledged. The effectiveness of ToC in different LMIC contexts would likely depend on its adaptation to local needs and challenges, as well as the commitment and involvement of local stakeholders.   1. **Application in Healthcare Interventions**: ToC has been established as an effective approach for designing and evaluating interventions in the health sector. This is particularly significant in the context of LMICs, where healthcare challenges can be complex and multifaceted. For instance, Barnhart et al. (2020) developed a ToC for the Better Birth program, a maternal and child health initiative, by analyzing three different implementation phases. Similarly, Aggarwal (2021) proposed a ToC model for delivering psychosocial interventions aimed at reducing self-harm in low and middle-income countries. These examples illustrate how ToC is used for planning, implementing, and evaluating healthcare programs in LMICs​​. **(Integration of evidence into Theory of Change frameworks in the healthcare sector: A rapid systematic review)** 2. **Case Study of SolidarMed in Zambia**: A practical case study of the ToC model is seen in the health worker housing program by the nonprofit SolidarMed in rural Zambia. This program aimed to construct and renovate housing for healthcare workers to address staff shortages and improve health outcomes in rural areas. The ToC model helped SolidarMed clearly articulate its program, lay a foundation for monitoring and evaluation, and identify key assumptions and outcomes for its intervention. This approach enabled the organization to prioritize aspects of its program for data collection and adapt its strategies to enhance the impact​​​​. **(Theory of Change case study: SolidarMed)** 3. **USAID/Vietnam Theory of Change Training**: USAID/Vietnam provides another example of ToC application, offering training and resources on context- and evidence-driven theories of change. These resources include narratives and logic models adapted for specific interventions and are designed to guide teams in developing effective ToC frameworks. This approach demonstrates how ToC can be tailored to specific contexts and challenges, particularly in LMIC settings​​. **(https://usaidlearninglab.org/resources/theory-change-toc-samples)** |

| **21.A Framework for Scaling Up Health Interventions** | **Y=1  N=0** | **Justification of Decision** (If “yes”, How? If “no”, Why?) |
| --- | --- | --- |
| 1. ***Is there pragmatic adequacy?*** | | |
| Are there clear concrete, feasible suggestions for how the theory proposed can actually be used in LMICs?  Does the author of the theory make a clear explicit link to use in LMICs? | 1 | Four-phase approach (Set-up, Develop the Scalable Unit, Test of Scale-up, Go to Full Scale), appears feasible for LMICs, especially considering its successful application in African health initiatives. |
| Do ALL concepts in the theory possess relevance for LMICs?  i.e. Is it Relevant? | 1 | The concepts of setting up, developing a scalable unit, testing scale-up, and going to full scale are highly relevant for LMICs. They address critical aspects of scaling up health interventions in contexts with varying resources and infrastructure challenges. |
| Are all concepts of usefulness to LMICs included in the theory?  i.e. Is it Complete? | 1 | Includes comprehensive set of concepts essential for scaling up health interventions in LMICs, covering everything from initial setup to full-scale implementation and sustainability. |
| 1. ***Is theory clearly articulated?*** | | |
| Are all the included concepts understandable/defined? | 1 | Concepts are well-articulated and explained in the context of their application in real-world health system scenarios, suggesting a high level of understandability. |
| Are the linkages between concepts clearly articulated or displayed? | 1 | Framework clearly outlines the linkages between its various phases and components, demonstrating how each step contributes to the overall process of scaling up health interventions. |
| Does the theory clearly present propositions that could be tested/used in an LMIC context? | 0 | Does not explicitly formulate testable propositions for LMICs, but structure and demonstrated applications imply suitability for generating relevant propositions in these contexts. |
| 1. ***Is there evidence of operational and empirical adequacy?*** | | |
| Have any studies used the theory in an LMIC context? | 1 | Framework has been applied in national-scale health initiatives in Ghana and South Africa, showcasing its practical application in LMIC contexts. |
| Have any studies that have used the theory supported their findings with data that relates to the theory or propositions? | 1 | Framework development based on review of existing scale-up models and frameworks, combined with practical experience from large-scale initiatives in Africa, providing empirical support for its application. |
| Does the empirical evidence from LMICs support the theory or propositions? | 1 | Successful applications in Ghana and South Africa provide empirical evidence of effectiveness in LMICs. Acknowledges need for training, support systems, and sustainability planning as part of scale-up process, but does not explicitly discuss limitations specific to LMICs beyond these aspects.   1. **South Africa**: Framework was applied to prevention of mother-to-child transmission (PMTCT) of HIV program. It involved a sequential scale-up process starting from a demonstration phase in a few health districts, followed by expanding to more districts, and finally going to full scale across the country. This approach led to significant decline in HIV transmission rates​​. **(A framework for scaling up health interventions: lessons from large-scale improvement initiatives in Africa)** 2. **Ghana**: In Ghana, framework was utilized for a national health systems improvement initiative aimed at reducing child and maternal mortality. This project involved introducing quality improvement methods to enhance the application of evidence-based maternal and child health interventions​​. |

| **22.Interventions in Organizational and Community Context: A Framework for Building Evidence on Dissemination and Implementation in Health Services Research** | **Y=1  N=0** | **Justification of Decision** (If “yes”, How? If “no”, Why?) |
| --- | --- | --- |
| 1. ***Is there pragmatic adequacy?*** | | |
| Are there clear concrete, feasible suggestions for how the theory proposed can actually be used in LMICs?  Does the author of the theory make a clear explicit link to use in LMICs? | Not clear | Addresses effective dissemination and implementation of health interventions within community settings, emphasizing the importance of adapting to diverse healthcare contexts. It suggests the need for community-based participatory approaches, which could be feasible in LMICs considering their varied resource, cultural, and economic contexts. |
| Do ALL concepts in the theory possess relevance for LMICs?  i.e. Is it Relevant? | 1 | Concepts, such as understanding multi-layered community and healthcare contexts and the diffusion process of new practices, are relevant to LMICs. These concepts are fundamental to addressing the unique challenges and dynamics of LMICs. |
| Are all concepts of usefulness to LMICs included in the theory?  i.e. Is it Complete? | 1 | Includes essential concepts for LMICs, such as contextual factors, stages of diffusion, and intervention outcomes, which are crucial for the dissemination and implementation of health interventions in these regions. |
| 1. ***Is theory clearly articulated?*** | | |
| Are all the included concepts understandable/defined? | 1 | Provides theoretically-grounded understanding of its concepts, which should be clear and understandable for diverse LMIC settings, especially with its focus on practical application in varied healthcare contexts. |
| Are the linkages between concepts clearly articulated or displayed? | 1 | Framework explicitly outlines the linkages between its key components, such as contextual factors, diffusion stages, and intervention outcomes, aiding in understanding their interaction in LMIC contexts. |
| Does the theory clearly present propositions that could be tested/used in an LMIC context? | 1 | Presents a process for evaluating and implementing health interventions that are applicable to LMICs. However, specific testable propositions for LMICs are not detailed in the abstract or introduction, indicating a need for further exploration of LMIC-specific hypotheses. |
| 1. ***Is there evidence of operational and empirical adequacy?*** | | |
| Have any studies used the theory in an LMIC context? | Not clear | Does not specifically mention empirical studies from LMIC contexts, which suggests potential for future research and application. |
| Have any studies that have used the theory supported their findings with data that relates to the theory or propositions? | 1 | Bridging research and practice: models for dissemination and implementation research  Example: Community Health Workers in Low- and Middle-Income Countries: What Do We Know About Scaling Up and Sustainability? |
| Does the empirical evidence from LMICs support the theory or propositions? | Not clear | Focus on outcome and impact evaluations indicates a commitment to assessing empirical evidence. Doesn't specifically address empirical evidence from LMICs or limitations of the theory in these contexts, which may require further investigation. |

| **23.A Person-Focused Model of Care for the Twenty-First Century: A System-of-Systems Perspective** | **Y=1  N=0** | **Justification of Decision** (If “yes”, How? If “no”, Why?) |
| --- | --- | --- |
| 1. ***Is there pragmatic adequacy?*** | | |
| Are there clear concrete, feasible suggestions for how the theory proposed can actually be used in LMICs?  Does the author of the theory make a clear explicit link to use in LMICs? | Not clear | The model emphasizes the importance of integrating various aspects of health care, including physical, mental, and social health, within a system-of-systems (SoS) approach. This approach, focusing on complex adaptive systems (CAS), could be feasibly implemented in LMICs, especially since it does no1t require significant infrastructure investments but rather the realignment and integration of existing resources. |
| Do ALL concepts in the theory possess relevance for LMICs?  i.e. Is it Relevant? | 1 | The concepts central to this model, such as addressing multi-morbidity (MM) and focusing on the whole person rather than just the disease, are relevant to LMICs. These regions often face complex health challenges, including chronic diseases, which this model aims to address more effectively. |
| Are all concepts of usefulness to LMICs included in the theory?  i.e. Is it Complete? | 1 | The model is inclusive of essential concepts for understanding health care challenges in LMICs. It incorporates physical, mental, and social dimensions of health and recognizes the roles of various stakeholders, including caregivers and payers, which are critical in LMIC contexts. |
| 1. ***Is theory clearly articulated?*** | | |
| Are all the included concepts understandable/defined? | 1 | The concepts presented in the model, such as CAS and SoS, are defined within the context of healthcare delivery, making them understandable for diverse settings, including LMICs. |
| Are the linkages between concepts clearly articulated or displayed? | 1 | The model clearly articulates the linkages between different components of the healthcare system, such as the relationships among stakeholders and between stakeholders and the patient’s health, essential for understanding their interaction in LMIC settings. |
| Does the theory clearly present propositions that could be tested/used in an LMIC context? | 1 | The model presents a framework applicable to LMICs, focusing on person- and household-focused care. However, specific propositions tailored to LMICs would need further development and testing within these contexts. |
| 1. ***Is there evidence of operational and empirical adequacy?*** | | |
| Have any studies used the theory in an LMIC context? | Not clear | Does not specifically mention empirical studies applying the model in LMIC contexts. The pilot study discussed is based on a US population, indicating a need for further research and application in LMICs. |
| Have any studies that have used the theory supported their findings with data that relates to the theory or propositions? | 0 | While the model is supported by a pilot study, this study is conducted in the context of a developed country's healthcare system. Its applicability and support by empirical data in LMICs would need further exploration and validation. |
| Does the empirical evidence from LMICs support the theory or propositions? | 0 | The model's effectiveness in addressing the challenges of chronic diseases and multi-morbidity is supported by pilot study, but its limitations in LMIC contexts are not explicitly discussed. Further empirical evidence from LMICs would be beneficial to understand its effectiveness and limitations in these regions. |

| **24.The Sustainability of Evidence-Based Interventions and Practices in Public Health and Health Care** | **Y=1  N=0** | **Justification of Decision** (If “yes”, How? If “no”, Why?) |
| --- | --- | --- |
| 1. ***Is there pragmatic adequacy?*** | | |
| Are there clear concrete, feasible suggestions for how the theory proposed can actually be used in LMICs?  Does the author of the theory make a clear explicit link to use in LMICs? | 1 | The sustainability of evidence-based interventions (EBIs) in LMICs can be challenging, especially when interventions are adapted from well-resourced settings and unrepresentative populations. It's important for interventions to fit the local context, considering factors like resources, cultural differences, and economic conditions of LMICs. |
| Do ALL concepts in the theory possess relevance for LMICs?  i.e. Is it Relevant? | 1 | The concepts in sustainability research, such as adaptation to local contexts and community engagement, are highly relevant to LMICs. The need to understand and adapt to the local context, including cultural norms and values, is critical for the success of EBIs in LMICs. |
| Are all concepts of usefulness to LMICs included in the theory?  i.e. Is it Complete? | 1 | The sustainability frameworks include essential concepts such as community engagement and adaptability, which are vital for LMICs. However, more research is needed to identify all relevant concepts and their applicability in the unique contexts of LMICs. |
| 1. ***Is theory clearly articulated?*** | | |
| Are all the included concepts understandable/defined? | 1 | While sustainability concepts are increasingly defined and discussed, there's still a lack of common definitions and conceptual clarity in the field. This may impact the understandability of these concepts in diverse LMIC settings. |
| Are the linkages between concepts clearly articulated or displayed? | 0 | The relationship between various sustainability factors and outcomes is complex and not fully understood. There's a need for more empirical research to clarify these linkages and their applicability in different contexts, including LMICs. |
| Does the theory clearly present propositions that could be tested/used in an LMIC context? | 0 | Sustainability research often lacks clear propositions specific to LMICs. While there's growing interest in adapting interventions to local contexts, more focused research is needed to develop testable propositions for these settings. |
| 1. ***Is there evidence of operational and empirical adequacy?*** | | |
| Have any studies used the theory in an LMIC context? | 1 | Studies have been conducted in LMICs, particularly in Africa, highlighting the importance of community engagement and understanding the local context. However, gaps remain in understanding the sustainability of interventions for specific health issues like chronic diseases in these regions. |
| Have any studies that have used the theory supported their findings with data that relates to the theory or propositions? | 1 | Empirical research on sustainability in LMICs indicates the importance of factors like community engagement and adapting interventions to local contexts. However, variability in how sustainability is defined and measured, making it challenging to generalize findings across different LMIC settings. |
| Does the empirical evidence from LMICs support the theory or propositions? | 1 | Empirical evidence from LMICs supports importance of adapting interventions to local contexts for sustainability. However, further research is needed to understand the impact of adaptations on intervention effectiveness and sustainability in these regions. Additionally, distinction between sustainability and concepts like reinvention and evolution needs further exploration. |

| **25.Evaluating the Public Health Impact of Health Promotion Interventions: The RE-AIM Framework** | **Y=1  N=0** | **Justification of Decision** (If “yes”, How? If “no”, Why?) |
| --- | --- | --- |
| 1. ***Is there pragmatic adequacy?*** | | |
| Are there clear concrete, feasible suggestions for how the theory proposed can actually be used in LMICs?  Does the author of the theory make a clear explicit link to use in LMICs? | 1 | The RE-AIM framework, focusing on the dimensions of reach, efficacy, adoption, implementation, and maintenance, appears to be adaptable for evaluating public health interventions in LMICs. However, challenges might arise due to differences in available resources, cultural contexts, and health systems compared to more controlled environments where many interventions are initially developed. |
| Do ALL concepts in the theory possess relevance for LMICs?  i.e. Is it Relevant? | 1 | All concepts within the RE-AIM framework—reach, efficacy, adoption, implementation, and maintenance—are relevant to LMICs. These concepts address the critical aspects of public health interventions, including their effectiveness, acceptability, and long-term sustainability in diverse settings. |
| Are all concepts of usefulness to LMICs included in the theory?  i.e. Is it Complete? | 1 | Includes essential concepts for LMICs. However, the specific challenges and needs of these regions, such as dealing with scarce resources or varying health infrastructure, may require additional considerations beyond the five dimensions of the RE-AIM framework. |
| 1. ***Is theory clearly articulated?*** | | |
| Are all the included concepts understandable/defined? | 1 | Concepts are clearly defined and should be understandable in context of LMICs. Focus on both individual-level measures (like reach and efficacy) and organizational/community-level measures (such as adoption and maintenance) is especially pertinent for diverse LMIC settings. |
| Are the linkages between concepts clearly articulated or displayed? | 1 | Articulates linkages between its components, emphasizing that the overall impact of an intervention results from the combined effects of reach, efficacy, adoption, implementation, and maintenance. Holistic approach is beneficial for understanding the interplay of these factors in LMICs. |
| Does the theory clearly present propositions that could be tested/used in an LMIC context? | 1 | Provides comprehensive structure for evaluating interventions. Specific propositions or hypotheses tailored for LMIC contexts not explicitly detailed. Applying framework to LMICs would require contextual adaptation and possibly the development of additional propositions relevant to these settings. |
| 1. ***Is there evidence of operational and empirical adequacy?*** | | |
| Have any studies used the theory in an LMIC context? | 1 | RE-AIM framework has been applied in low and middle-income countries (LMICs) for various health-related interventions.  For example, a study used the RE-AIM framework to guide the evaluation of clean fuel cooking programs in several LMICs[107]. |
| Have any studies that have used the theory supported their findings with data that relates to the theory or propositions? | 1 | the RE-AIM framework was used to evaluate the implementation of the Friendship Bench program in Zimbabwe. This study operationalized the RE-AIM framework to create quantitative indicators based on available data from the Friendship Bench implementation, covering three of the five RE-AIM domains: Reach, Adoption, and Implementation. This study was carried out in 36 primary health care clinics in three cities, contributing to the understanding of the program's impact and implementation in a LMIC context​[108]. |
| Does the empirical evidence from LMICs support the theory or propositions? | 1 | Example: Applying the RE-AIM conceptual framework for the promotion of physical activity in low-and middle-income countries |

| **26.The RE-AIM Framework: A Systematic Review of Use Over Time** | **Y=1  N=0** | **Justification of Decision** (If “yes”, How? If “no”, Why?) |
| --- | --- | --- |
| 1. ***Is there pragmatic adequacy?*** | | |
| Are there clear concrete, feasible suggestions for how the theory proposed can actually be used in LMICs?  Does the author of the theory make a clear explicit link to use in LMICs? | 1 | The RE-AIM framework, with its focus on key aspects like reach, effectiveness, adoption, implementation, and maintenance, can be adaptable for LMICs. However, challenges arise from wide-scale implementation barriers at multiple levels, including citizens, practitioners, organizational, community, and policy levels, which are prevalent in LMICs. |
| Do ALL concepts in the theory possess relevance for LMICs?  i.e. Is it Relevant? | 1 | Concepts are relevant to LMICs as they encompass crucial dimensions of health interventions, like reach (the extent of participation in the target population), effectiveness, adoption by settings and intervention agents, implementation fidelity, and maintenance at both individual and setting levels. |
| Are all concepts of usefulness to LMICs included in the theory?  i.e. Is it Complete? | 1 | Appears to include essential concepts for understanding and addressing health challenges in LMICs. However, given the unique complexities of LMICs, additional context-specific considerations might be necessary. |
| 1. ***Is theory clearly articulated?*** | | |
| Are all the included concepts understandable/defined? | 1 | Concepts are well-defined and should be understandable in diverse LMIC settings. However, broad range of settings and conditions in LMICs might require further contextual adaptation and explanation. |
| Are the linkages between concepts clearly articulated or displayed? | 1 | Presents clear linkages between dimensions. Precise nature of these relationships and their combined impact on public health is complex and not fully understood, which could pose challenges in LMICs where health systems and intervention contexts vary greatly. |
| Does the theory clearly present propositions that could be tested/used in an LMIC context? | 1 | Provides comprehensive structure for evaluating interventions. |
| 1. ***Is there evidence of operational and empirical adequacy?*** | | |
| Have any studies used the theory in an LMIC context? | 1 | RE-AIM framework has been applied in low and middle-income countries (LMICs) for various health-related interventions.  For example, a study used the RE-AIM framework to guide the evaluation of clean fuel cooking programs in several LMICs[107]. |
| Have any studies that have used the theory supported their findings with data that relates to the theory or propositions? | 1 | the RE-AIM framework was used to evaluate the implementation of the Friendship Bench program in Zimbabwe. This study operationalized the RE-AIM framework to create quantitative indicators based on available data from the Friendship Bench implementation, covering three of the five RE-AIM domains: Reach, Adoption, and Implementation. This study was carried out in 36 primary health care clinics in three cities, contributing to the understanding of the program's impact and implementation in a LMIC context​[108]. |
| Does the empirical evidence from LMICs support the theory or propositions? | 1 | Example: Applying the RE-AIM conceptual framework for the promotion of physical activity in low-and middle-income countries |

| **27.The FRAME: an expanded framework for reporting adaptations and modifications to evidence-based interventions** | **Y=1  N=0** | **Justification of Decision** (If “yes”, How? If “no”, Why?) |
| --- | --- | --- |
| 1. ***Is there pragmatic adequacy?*** | | |
| Are there clear concrete, feasible suggestions for how the theory proposed can actually be used in LMICs?  Does the author of the theory make a clear explicit link to use in LMICs? | 0 | The FRAME framework, designed for characterizing modifications to interventions, seems adaptable for LMIC contexts. It considers planned and unplanned modifications, which are essential in the diverse and often resource-limited settings of LMICs. |
| Do ALL concepts in the theory possess relevance for LMICs?  i.e. Is it Relevant? | 1 | The concepts of adaptation and modification in the FRAME framework are highly relevant to LMICs. These concepts cover a broad range of changes that can occur in interventions, addressing both proactive adaptations and reactive modifications due to unanticipated challenges. |
| Are all concepts of usefulness to LMICs included in the theory?  i.e. Is it Complete? | 1 | FRAME includes essential concepts for understanding modifications in LMICs, such as the nature of the modification, the reason behind it, and its alignment with the original intervention’s goals. However, specific LMIC challenges like infrastructure, resource constraints, and cultural diversity may require further consideration. |
| 1. ***Is theory clearly articulated?*** | | |
| Are all the included concepts understandable/defined? | 1 | Concepts are well-defined and should be understandable in diverse LMIC settings. Comprehensive approach to documenting modifications caters to the complex implementation landscapes in LMICs. |
| Are the linkages between concepts clearly articulated or displayed? | 1 | Provides clear linkages between types of modifications, their reasons, and the intended and unintended consequences. This clarity is beneficial for understanding the impact of modifications in the varied contexts of LMICs. |
| Does the theory clearly present propositions that could be tested/used in an LMIC context? | 0 | Offers a comprehensive structure for evaluating and documenting interventions. Specific propositions or hypotheses tailored for LMIC contexts not explicitly detailed. Applying to these settings would likely require contextual adaptation. |
| 1. ***Is there evidence of operational and empirical adequacy?*** | | |
| Have any studies used the theory in an LMIC context? | 0 |  |
| Have any studies that have used the theory supported their findings with data that relates to the theory or propositions? | Not clear |  |
| Does the empirical evidence from LMICs support the theory or propositions? | 0 | The effectiveness of FRAME in LMICs is not explicitly addressed. More empirical evidence and application studies in LMICs are needed to understand its effectiveness and limitations in these regions. Stakeholders have identified the need to balance comprehensiveness with feasibility and pragmatism in documentation and reporting, which is particularly relevant in LMICs. |

| **28.Practical guidance for scaling up health service innovations** | **Y=1  N=0** | **Justification of Decision** (If “yes”, How? If “no”, Why?) |
| --- | --- | --- |
| 1. ***Is there pragmatic adequacy?*** | | |
| Are there clear concrete, feasible suggestions for how the theory proposed can actually be used in LMICs?  Does the author of the theory make a clear explicit link to use in LMICs? | 0 | Acknowledges complexity of scaling up health service innovations, emphasizing need for locally generated evidence of programmatic effectiveness and feasibility. Also notes the requirement of special technical, managerial, and financial resources, which may pose challenges in LMICs due to resource limitations. |
| Do ALL concepts in the theory possess relevance for LMICs?  i.e. Is it Relevant? | 1 | The concepts of scaling up are relevant to LMICs. Focuses on expanding tested health service innovations to benefit more people and foster sustainable policy and program development. |
| Are all concepts of usefulness to LMICs included in the theory?  i.e. Is it Complete? | 1 | Covers essential concepts for LMICs, including the need for innovations to be realistic, relevant, and to have a beneficial impact on health concerns. It highlights the importance of testing interventions under real-life conditions, which is critical in LMICs. |
| 1. ***Is theory clearly articulated?*** | | |
| Are all the included concepts understandable/defined? | 1 | Guide provides clear definitions and explanations of the scaling-up process, which should be understandable in diverse LMIC settings. It emphasizes the importance of contextually relevant innovations and the need for careful planning and testing. |
| Are the linkages between concepts clearly articulated or displayed? | 1 | Framework presents clear linkages among the innovation, user organization, environment, resource team, and the scaling-up strategy. Emphasizes importance of understanding the dynamic interplay of these elements, particularly in the varied contexts of LMICs. |
| Does the theory clearly present propositions that could be tested/used in an LMIC context? | 1 | Provides comprehensive framework for scaling up but does not offer specific propositions tailored for LMIC contexts. Applying this framework in LMICs would require contextual adaptation and potentially the development of additional propositions relevant to these settings. |
| 1. ***Is there evidence of operational and empirical adequacy?*** | | |
| Have any studies used the theory in an LMIC context? | 0 | Guide is intended for public health programme managers, donors, and technical assistance providers involved in scaling up health service innovations, which is relevant for LMICs. However, does not specifically discuss application in LMICs, suggesting need for further research and application in these regions. |
| Have any studies that have used the theory supported their findings with data that relates to the theory or propositions? | 0 |  |
| Does the empirical evidence from LMICs support the theory or propositions? DO authors of empirical studies identify any limitations of the theory? If so, what are they? | 0 | Effectiveness of scaling-up framework in LMICs is not explicitly addressed. More empirical evidence and detailed application studies in LMICs would be beneficial to understand its effectiveness and limitations in these regions. |

| **29.Framework for the establishment of a feasible tailored and effective perinatal education programme** | **Y=1  N=0** | **Justification of Decision** (If “yes”, How? If “no”, Why?) |
| --- | --- | --- |
| 1. ***Is there pragmatic adequacy?*** | | |
| Are there clear concrete, feasible suggestions for how the theory proposed can actually be used in LMICs?  Does the author of the theory make a clear explicit link to use in LMICs? | 0 | Aims to adapt antenatal education (AE) to the needs of women, considering factors such as local healthcare systems and cultural contexts. This approach seems feasible for LMICs as it emphasizes adaptability and relevance to women's specific circumstances. |
| Do ALL concepts in the theory possess relevance for LMICs?  i.e. Is it Relevant? | 1 | Concepts, such as personalized perinatal education and incorporating the needs of diverse populations, are relevant to LMICs. Concepts address the essential aspects of maternal and child health, critical in these regions. |
| Are all concepts of usefulness to LMICs included in the theory?  i.e. Is it Complete? | 1 | Framework includes essential concepts like need for flexibility, covering the entire process from pre-pregnancy to postpartum, and involving the woman's partner, family, and community. These concepts are vital for LMICs, where diverse cultural and socioeconomic factors play a significant role. |
| 1. ***Is theory clearly articulated?*** | | |
| Are all the included concepts understandable/defined? | 1 | Concepts are well-defined and should be understandable in diverse LMIC settings. It focuses on practical and adaptable solutions to maternal and child health, which is pertinent for LMICs. |
| Are the linkages between concepts clearly articulated or displayed? | 1 | Provides clear linkages between its components, such as the need for flexibility, inclusion of the partner and family, and continuous evaluation and adaptation. This clarity is beneficial for understanding the impact of these factors in the varied contexts of LMICs. |
| Does the theory clearly present propositions that could be tested/used in an LMIC context? | 0 | Provides comprehensive structure for developing new perinatal education programs, but specific propositions tailored for LMIC contexts are not explicitly detailed. Applying this framework in LMICs would require contextual adaptation. |
| 1. ***Is there evidence of operational and empirical adequacy?*** | | |
| Have any studies used the theory in an LMIC context? | Not clear | Does not specifically discuss application to LMICs. Future research and application in these regions are necessary to assess its practicality and effectiveness. |
| Have any studies that have used the theory supported their findings with data that relates to the theory or propositions? | 1 |  |
| Does the empirical evidence from LMICs support the theory or propositions? | 0 | Effectiveness of framework in LMICs not explicitly addressed. More empirical evidence and application studies in LMICs would be beneficial to understand its effectiveness and limitations in these regions. Recognizes need for continuous evaluation and adaptation, which is crucial for its application in diverse LMIC settings. |

| **30.A framework for cross-cultural development and implementation of complex interventions to improve palliative care in nursing homes: the PACE Steps to Success programme** | **Y=1  N=0** | **Justification of Decision** (If “yes”, How? If “no”, Why?) |
| --- | --- | --- |
| 1. ***Is there pragmatic adequacy?*** | | |
| Are there clear concrete, feasible suggestions for how the theory proposed can actually be used in LMICs?  Does the author of the theory make a clear explicit link to use in LMICs? | 0 | The PACE Steps to Success programme is a complex educational and development intervention aimed at improving palliative care in nursing homes. Its implementation in LMICs would need to consider differences in health and social care systems, legal and regulatory policies, and cultural norms. The cross-cultural adaptation and implementation framework described may need adjustments for LMIC contexts due to varying resource availability and healthcare infrastructure. |
| Do ALL concepts in the theory possess relevance for LMICs?  i.e. Is it Relevant? | 1 | The concepts of the programme, focusing on improving palliative care, are relevant to LMICs. Palliative care is an essential aspect of healthcare that addresses physical, psychosocial, and spiritual needs, which are universally pertinent, including in LMICs. |
| Are all concepts of usefulness to LMICs included in the theory?  i.e. Is it Complete? | 1 | Includes essential concepts for cross-cultural adaptation and implementation of palliative care interventions. However, specific adaptations for LMICs, considering their unique healthcare challenges and resources, would be necessary. |
| 1. ***Is theory clearly articulated?*** | | |
| Are all the included concepts understandable/defined? | 1 | The programme's concepts and framework are well-defined and could be understandable in LMIC settings, given a focus on contextual relevance and cultural sensitivity. |
| Are the linkages between concepts clearly articulated or displayed? | 1 | Provides clear linkages between the adaptation of resources, training in the intervention, and support during implementation. These linkages are crucial for understanding how different components interact and affect palliative care in nursing homes, which would be beneficial for LMIC contexts. |
| Does the theory clearly present propositions that could be tested/used in an LMIC context? | 0 | Offers structured approach for implementing the programme but may not provide specific propositions tailored for LMICs. Applying to LMICs would require contextual adaptation and possibly the development of additional propositions relevant to these settings. |
| 1. ***Is there evidence of operational and empirical adequacy?*** | | |
| Have any studies used the theory in an LMIC context? | 0 | Does not specifically discuss application to LMICs. Programme largely implemented in well-resourced countries, indicating need for further research and application in LMICs. |
| Have any studies that have used the theory supported their findings with data that relates to the theory or propositions? | 0 | Based on implementation of programme in European countries. While it provides a structured approach, support from empirical data in LMIC contexts remains to be established, as it needs further research and application in these settings. |
| Does the empirical evidence from LMICs support the theory or propositions? | 0 | Effectiveness for LMICs is not explicitly addressed. More empirical evidence and application studies in LMICs would be beneficial to understand its effectiveness and limitations. Integration of cross-cultural development within the implementation process is a strength, but applicability in constantly shifting and transforming environment of nursing homes, especially in LMICs, remains a challenge. |

| **31.Explaining high and low performers in complex intervention trials: a new model based on diffusion of innovations theory** | **Y=1  N=0** | **Justification of Decision** (If “yes”, How? If “no”, Why?) |
| --- | --- | --- |
| 1. ***Is there pragmatic adequacy?*** | | |
| Are there clear concrete, feasible suggestions for how the theory proposed can actually be used in LMICs?  Does the author of the theory make a clear explicit link to use in LMICs? | 0 | The trial focused on rapid HIV testing in general practice and required health care organizations to implement new service models. While the study provides insights into factors influencing the success of such interventions, adapting this model to LMICs would require considering specific challenges like resource constraints, cultural differences, and existing health system infrastructure. |
| Do ALL concepts in the theory possess relevance for LMICs?  i.e. Is it Relevant? | 1 | The concepts of diffusion of innovations theory, which was used to explain variation in trial performance, are relevant to LMICs. This theory includes understanding the innovation, adopters, communication, organizational context, and implementation process, which are vital for successful health interventions in LMICs. |
| Are all concepts of usefulness to LMICs included in the theory?  i.e. Is it Complete? | 1 | The model addresses key concepts like innovation adoption, organizational readiness, and the importance of leadership and managerial relations. These concepts are essential for understanding the successful implementation of health interventions in LMICs, although additional context-specific factors may need to be included. |
| 1. ***Is theory clearly articulated?*** | | |
| Are all the included concepts understandable/defined? | 1 | The concepts and model used in the trial are well-defined and could be understandable in diverse LMIC settings. However, the complexity of the model may require additional explanation or adaptation for different cultural and organizational contexts in LMICs. |
| Are the linkages between concepts clearly articulated or displayed? | 1 | The study provides clear linkages between different components of the model, such as how organizational readiness, leadership, and staff perceptions affect the implementation of innovations. Understanding these linkages is crucial for applying this model in the varied contexts of LMICs. |
| Does the theory clearly present propositions that could be tested/used in an LMIC context? | 0 | While the study offers a structured approach for understanding and implementing complex health interventions, specific propositions tailored for LMIC contexts are not explicitly detailed. Applying this model in LMICs would likely require contextual adaptation. |
| 1. ***Is there evidence of operational and empirical adequacy?*** | | |
| Have any studies used the theory in an LMIC context? | 0 | Does not specifically discuss application of model in LMICs. Study findings are based on a trial conducted in a high-prevalence HIV area in London, suggesting need for further research and application in LMICs. |
| Have any studies that have used the theory supported their findings with data that relates to the theory or propositions? | 0 | Study is based on empirical data from a trial in a specific context. While it provides a structured approach, the support from empirical data in LMIC contexts remains to be established, as it would need further research and application in these settings. |
| Does the empirical evidence from LMICs support the theory or propositions? | 0 | The effectiveness of the model in LMICs is not explicitly addressed. More empirical evidence and application studies in LMICs would be beneficial to understand its effectiveness and limitations. Study emphasizes importance of understanding organizational culture and practices, which is crucial for its application in diverse LMIC settings. |

| **32.WICID framework version 1.0: criteria and considerations to guide evidence-­informed decision-­making on non-­pharmacological interventions targeting COVID-19** | **Y=1  N=0** | **Justification of Decision** (If “yes”, How? If “no”, Why?) |
| --- | --- | --- |
| 1. ***Is there pragmatic adequacy?*** | | |
| Are there clear concrete, feasible suggestions for how the theory proposed can actually be used in LMICs?  Does the author of the theory make a clear explicit link to use in LMICs? | 0 | The WICID framework, adapted from the WHO-INTEGRATE framework, is intended for decision-making on non-pharmacological interventions for COVID-19. While it provides a comprehensive structure, its feasibility in LMICs would depend on the ability to adapt it to local contexts, considering resource limitations, cultural differences, and varying health infrastructure. |
| Do ALL concepts in the theory possess relevance for LMICs?  i.e. Is it Relevant? | 1 | Concepts are relevant to LMICs, addressing a range of factors critical in managing the COVID-19 pandemic, such as health implications, human rights, societal impacts, and resource constraints. These aspects are crucial in the context of LMICs, where COVID-19 poses significant challenges. |
| Are all concepts of usefulness to LMICs included in the theory?  i.e. Is it Complete? | 1 | Includes essential concepts for managing the pandemic in LMICs, covering health, social, economic, and rights-related aspects. However, unique challenges of LMICs, such as disparities in healthcare access and economic constraints, may necessitate further tailoring. |
| ***If answer “yes” to question 1 then proceed to question 2. If answer “no” to question 1 then consider exclusion from review*** | | |
| **All papers that answer “yes” to question 1:** | **Y=1 N=0** | If “yes”, How? If “no”, Why? |
| 1. ***Is theory clearly articulated?*** | | |
| Are all the included concepts understandable/defined? | 1 | Concepts should be understandable in LMIC settings, given its grounding in the WHO-INTEGRATE framework and focus on practical decision-making criteria. However, LMICs might require additional guidance for applying these concepts in their specific contexts. |
| Are the linkages between concepts clearly articulated or displayed? | 1 | Outlines clear linkages between various decision-making criteria, which can aid in comprehensively addressing the multifaceted challenges of COVID-19. Understanding these linkages is essential for LMICs to effectively balance various factors in pandemic management. |
| Does the theory clearly present propositions that could be tested/used in an LMIC context? | 0 | While the WICID framework provides a structured approach for decision-making, it does not offer specific propositions tailored for LMIC contexts. Its application in LMICs would benefit from incorporating local data and experiences to form relevant propositions. |
| 1. ***Is there evidence of operational and empirical adequacy?*** | | |
| Have any studies used the theory in an LMIC context? | 0 | Does not specify application to LMICs. Developed based on comprehensive strategy documents from Germany, indicating need for adaptation and application in the diverse contexts of LMICs. |
| Have any studies that have used the theory supported their findings with data that relates to the theory or propositions? | 0 | Development involved analyzing comprehensive strategy documents, but does not provide specific empirical data from LMICs. Therefore, support from empirical data in LMIC contexts remains to be established. |
| Does the empirical evidence from LMICs support the theory or propositions? DO authors of empirical studies identify any limitations of the theory? If so, what are they? | 0 | Effectiveness of the WICID framework in LMICs not explicitly addressed. More empirical evidence and application studies in LMICs would be beneficial to understand its effectiveness and limitations, particularly considering the diverse challenges faced by these countries in managing COVID-19. |

| **33.Translating Policies Into Practice: A Framework to Prevent Childhood Obesity in Afterschool Programs** | **Y=1  N=0** | **Justification of Decision** (If “yes”, How? If “no”, Why?) |
| --- | --- | --- |
| 1. ***Is there pragmatic adequacy?*** | | |
| Are there clear concrete, feasible suggestions for how the theory proposed can actually be used in LMICs?  Does the author of the theory make a clear explicit link to use in LMICs? | 0 | Primarily focuses on U.S. context, with emphasis on policies and practices relevant to afterschool programs in this setting. Its direct applicability to LMICs is not clearly outlined, and factors such as different resource availabilities, cultural norms, and economic conditions in LMICs are not specifically addressed. |
| Do ALL concepts in the theory possess relevance for LMICs?  i.e. Is it Relevant? | 0 | Concepts discussed are highly relevant to the context of childhood obesity and physical activity. Doesn't specifically address how these concepts might be relevant or adapted to the unique challenges and needs of LMICs. |
| Are all concepts of usefulness to LMICs included in the theory?  i.e. Is it Complete? | 0 | Includes essential concepts related to policy implementation, organizational change, and public health, which are crucial for understanding and addressing childhood obesity in afterschool settings. However, doesn’t explicitly include or exclude concepts specific to LMICs. |
| ***If answer “yes” to question 1 then proceed to question 2. If answer “no” to question 1 then consider exclusion from review*** | | |
| **All papers that answer “yes” to question 1:** | **Y=1 N=0** | If “yes”, How? If “no”, Why? |
| 1. ***Is theory clearly articulated?*** | | |
| Are all the included concepts understandable/defined? | 1 | Concepts are clearly defined and presented in a manner that is understandable to those familiar with public health, policy implementation, and organizational change. |
| Are the linkages between concepts clearly articulated or displayed? | 1 | Effectively articulates clear linkages between its concepts, especially how different elements like policy environment, individual characteristics, and organizational partnerships interact and impact each other in the context of afterschool programs. |
| Does the theory clearly present propositions that could be tested/used in an LMIC context? | 0 | The study doesn't provide specific testable propositions or hypotheses relevant to LMIC settings, as its focus is more on the U.S. context. |
| 1. ***Is there evidence of operational and empirical adequacy?*** | | |
| Have any studies used the theory in an LMIC context? | 0 | No direct evidence or discussion about the application of this framework in empirical studies within LMIC contexts. |
| Have any studies that have used the theory supported their findings with data that relates to the theory or propositions? | 0 | Grounded in empirical data relevant to the U.S. context, but it does not provide data specifically related to LMICs. |
| Does the empirical evidence from LMICs support the theory or propositions? DO authors of empirical studies identify any limitations of the theory? If so, what are they? | 0 | Acknowledges limitations in the current policy implementation and the need for a more systematic approach. However, doesn’t specifically address empirical evidence or limitations in the context of LMICs. |

| **34.Moving alcohol prevention research forward—Part I: Introducing a complex systems paradigm** | **Y=1  N=0** | **Justification of Decision** (If “yes”, How? If “no”, Why?) |
| --- | --- | --- |
| 1. ***Is there pragmatic adequacy?*** | | |
| Are there clear concrete, feasible suggestions for how the theory proposed can actually be used in LMICs?  Does the author of the theory make a clear explicit link to use in LMICs? | 0 | Does not specifically address the feasibility of the complex systems paradigm in low- and middle-income countries (LMICs). The focus is primarily on the context of college drinking in the United States, without direct mention of resources, cultural differences, or economic conditions in LMICs. |
| Do ALL concepts in the theory possess relevance for LMICs?  i.e. Is it Relevant? | 0 | While concepts of complex systems and computational modeling are universally relevant, does not explicitly discuss their relevance in the context of LMICs. Research is centered on alcohol misuse among college students in the U.S., with a specific emphasis on socio-ecological factors, which might not directly align with the unique challenges in LMICs. |
| Are all concepts of usefulness to LMICs included in the theory?  i.e. Is it Complete? | 0 | The theory incorporates a comprehensive range of concepts relevant to understanding alcohol misuse in a college environment, including socio-ecological factors, system dynamics, and computational modeling. However, it doesn't explicitly cover concepts that might be unique to LMICs. |
| 1. ***Is theory clearly articulated?*** | | |
| Are all the included concepts understandable/defined? | 1 | Presents concepts with sufficient clarity for an audience familiar with public health research and computational modeling. However, complexity of concepts might pose challenges in terms of understandability, particularly in diverse LMIC settings. |
| Are the linkages between concepts clearly articulated or displayed? | 1 | Effectively articulates the linkages between various factors influencing alcohol misuse in college environments, demonstrating the interplay of individual, group-level, and environmental factors. However, these linkages are not explicitly extended to or examined within LMIC contexts. |
| Does the theory clearly present propositions that could be tested/used in an LMIC context? | 0 | Does not offer specific testable propositions or hypotheses relevant to LMIC settings. Focus remains on U.S. context, limiting direct applicability of its propositions to LMICs. |
| 1. ***Is there evidence of operational and empirical adequacy?*** | | |
| Have any studies used the theory in an LMIC context? | 0 | No mention of empirical studies applying this complex systems paradigm in LMIC contexts. The discussion and examples are centered on the U.S., specifically regarding college student alcohol misuse. |
| Have any studies that have used the theory supported their findings with data that relates to the theory or propositions? | 0 | Supports arguments with data and examples pertinent to the U.S. context. While this underscores the theory's applicability in that setting, it does not provide direct evidence or data relevant to LMICs. |
| Does the empirical evidence from LMICs support the theory or propositions? DO authors of empirical studies identify any limitations of the theory? If so, what are they? | 0 | Acknowledges limitations of traditional approaches in alcohol prevention and advocates for a paradigm shift. However, does not specifically address empirical evidence or limitations in the context of LMICs. Instead, it focuses on potential and challenges of implementing complex systems approaches more generally. |

| **35.Organizational theory for dissemination and implementation research** | **Y=1  N=0** | **Justification of Decision** (If “yes”, How? If “no”, Why?) |
| --- | --- | --- |
| 1. ***Is there pragmatic adequacy?*** | | |
| Are there clear concrete, feasible suggestions for how the theory proposed can actually be used in LMICs?  Does the author of the theory make a clear explicit link to use in LMICs? | 0 | Article doesn't provide explicit, concrete suggestions for the application of the theory in LMICs. The focus is predominantly on the theoretical understanding of SafeCare's implementation in various settings, primarily in developed contexts. |
| Do ALL concepts in the theory possess relevance for LMICs?  i.e. Is it Relevant? | 0 | Not all concepts might be directly relevant to LMICs, as the study is largely contextualized within developed countries' settings. The applicability might vary depending on specific LMIC contexts. |
| Are all concepts of usefulness to LMICs included in the theory?  i.e. Is it Complete? | 0 | Article does not explicitly address whether all relevant concepts for LMICs are included in the theory. It focuses more on the general applicability of organizational theories. |
| ***If answer “yes” to question 1 then proceed to question 2. If answer “no” to question 1 then consider exclusion from review*** | | |
| **All papers that answer “yes” to question 1:** | **Y=1 N=0** | If “yes”, How? If “no”, Why? |
| 1. ***Is theory clearly articulated?*** | | |
| Are all the included concepts understandable/defined? | 1 | The concepts in the theory are well-defined and explained with regards to their application in the context of SafeCare’s implementation. |
| Are the linkages between concepts clearly articulated or displayed? | 1 | Articulates linkages between different organizational theories and their application to the implementation process, although this is done within the context of developed countries. |
| Does the theory clearly present propositions that could be tested/used in an LMIC context? | 0 | Does not provide clear, distinct propositions for testing or using the theory specifically in an LMIC context. |
| 1. ***Is there evidence of operational and empirical adequacy?*** | | |
| Have any studies used the theory in an LMIC context? | 0 | No specific mention of studies using this theory in an LMIC context. |
| Have any studies that have used the theory supported their findings with data that relates to the theory or propositions? | 0 | Document does not provide evidence of empirical studies in LMICs that support the theory or propositions related to LMICs. |
| Does the empirical evidence from LMICs support the theory or propositions? DO authors of empirical studies identify any limitations of the theory? If so, what are they? | 0 | Paper does not address empirical evidence from LMICs in relation to the theory or propositions, nor does it discuss the limitations of the theory as identified in empirical studies from LMICs. |

| **36. Development and application of a hybrid implementation research framework to understand success in reducing under-5 mortality in Rwanda** | **Y=1  N=0** | **Justification of Decision** (If “yes”, How? If “no”, Why?) |
| --- | --- | --- |
| 1. ***Is there pragmatic adequacy?*** | | |
| Are there clear concrete, feasible suggestions for how the theory proposed can actually be used in LMICs?  Does the author of the theory make a clear explicit link to use in LMICs? | 1 | Provides comprehensive framework specifically designed for understanding and implementing strategies to reduce under-5 mortality in LMICs like Rwanda. Details adaptation of existing frameworks to suit LMIC contexts, offering practical suggestions for implementation. |
| Do ALL concepts in the theory possess relevance for LMICs?  i.e. Is it Relevant? | 1 | Concepts and strategies are tailored for LMICs, focusing on contextual factors like health system design, national leadership, community involvement, and equity in healthcare access. |
| Are all concepts of usefulness to LMICs included in the theory?  i.e. Is it Complete? | 1 | Incorporates relevant concepts essential for LMICs. It includes strategies for implementation, adaptation, and sustainment of health interventions, emphasizing the importance of contextual factors specific to LMICs. |
| 1. ***Is theory clearly articulated?*** | | |
| Are all the included concepts understandable/defined? | 1 | Clearly defines and explains concepts. Provides detailed descriptions of the implementation steps, strategies, and contextual factors involved. |
| Are the linkages between concepts clearly articulated or displayed? | 1 | Demonstrates clear linkages between various concepts. Outlines systematic approach from the identification of EBIs to their implementation and sustainment, considering interplay of contextual factors at different levels. |
| Does the theory clearly present propositions that could be tested/used in an LMIC context? | 0 | Offers clear propositions for the LMIC context, focusing on implementation steps, strategies, and contextual factors relevant to health interventions in these regions. |
| 1. ***Is there evidence of operational and empirical adequacy?*** | | |
| Have any studies used the theory in an LMIC context? | 1 | Applies framework in LMIC context of Rwanda, illustrating its practical use in understanding and improving health outcomes. |
| Have any studies that have used the theory supported their findings with data that relates to the theory or propositions? | 1 | Supported with empirical data from Rwanda's experience in reducing under-5 mortality, showcasing successful application and outcomes. |
| Does the empirical evidence from LMICs support the theory or propositions? DO authors of empirical studies identify any limitations of the theory? If so, what are they? | 1 | Case study of Rwanda provides empirical evidence supporting effectiveness in LMICs. Also identifies limitations such as reliance on available data and potential biases in key informant interviews. |

| **37.A tale of ‘politics and stars aligning’: analysing the sustainability of scaled up digital tools for front-line health workers in India** | **Y=1  N=0** | **Justification of Decision** (If “yes”, How? If “no”, Why?) |
| --- | --- | --- |
| 1. ***Is there pragmatic adequacy?*** | | |
| Are there clear concrete, feasible suggestions for how the theory proposed can actually be used in LMICs?  Does the author of the theory make a clear explicit link to use in LMICs? | 1 | The study provides insights into the scalability and sustainability of digital health tools in India, which can be relevant for LMICs. It emphasizes the importance of strong government leadership, stakeholder collaboration, and a supportive ecosystem. |
| Do ALL concepts in the theory possess relevance for LMICs?  i.e. Is it Relevant? | 1 | The concepts discussed are relevant for LMICs, especially in the context of scaling digital health solutions. The study highlights factors like government support, stakeholder networks, and adaptability of digital solutions, all crucial for LMICs. |
| Are all concepts of usefulness to LMICs included in the theory?  i.e. Is it Complete? | 1 | The study includes relevant concepts for LMICs, such as the need for adaptability of digital solutions to local contexts, importance of stakeholder engagement, and challenges in data governance and sustainability. |
| 1. ***Is theory clearly articulated?*** | | |
| Are all the included concepts understandable/defined? | 1 | Clearly defines and explains the concepts, focusing on the sustainability and scalability of digital health solutions, the roles of various stakeholders, and the importance of adaptability and government support. |
| Are the linkages between concepts clearly articulated or displayed? | 1 | Effectively articulates linkages between the digital solution characteristics, actor roles, implementation processes, and context, emphasizing how these factors interact to influence sustainability. |
| Does the theory clearly present propositions that could be tested/used in an LMIC context? | 0 | Study presents clear propositions for LMICs, highlighting the importance of adaptability, stakeholder engagement, government support, and the need for a supportive ecosystem for digital health solutions. |
| 1. ***Is there evidence of operational and empirical adequacy?*** | | |
| Have any studies used the theory in an LMIC context? | 1 | Study is example of applying theoretical concepts in the context of LMICs, specifically India. |
| Have any studies that have used the theory supported their findings with data that relates to the theory or propositions? | 1 | Supports its theoretical propositions with empirical data from the Indian context, detailing the experiences with scaling up digital health tools. |
| Does the empirical evidence from LMICs support the theory or propositions? DO authors of empirical studies identify any limitations of the theory? If so, what are they? | 1 | Empirical evidence from the Indian context supports study propositions about the importance of government support, stakeholder engagement, and adaptability for sustainability. Does not explicitly discuss limitations of the theory based on empirical evidence. |
